# Multiple‐Porphyrin Functionalized Hexabenzocoronenes

**DOI:** 10.1002/chem.201903113

**Published:** 2019-10-22

**Authors:** Max M. Martin, Dominik Lungerich, Frank Hampel, Jens Langer, Tanya K. Ronson, Norbert Jux

**Affiliations:** ^1^ Department of Chemistry and Pharmacy & Interdisciplinary Center for, Molecular Materials (ICMM), Organic Chemistry II Friedrich-Alexander-University Erlangen-Nürnberg Nikolaus-Fiebiger-Strasse 10 91058 Erlangen Germany; ^2^ Inorganic and Organometallic Chemistry Egerlandstrasse 1 91058 Erlangen Germany; ^3^ Department of Chemistry University of Cambridge Lensfield Road Cambridge CB2 1EW UK

**Keywords:** hexabenzocoronene, nanographene, nanostructures, porphyrinoids, Scholl oxidation

## Abstract

Porphyrin–hexabenzocoronene architectures serve as good model compounds to study light‐harvesting systems. Herein, the synthesis of porphyrin functionalized hexa‐*peri*‐hexabenzocoronenes (HBCs), in which one or more porphyrins are covalently linked to a central HBC core, is presented. A series of hexaphenylbenzenes (HPBs) was prepared and reacted under oxidative coupling conditions. The transformation to the respective HBC derivatives worked well with mono‐ and tri‐porphyrin‐substituted HPBs. However, if more porphyrins are attached to the HPB core, Scholl oxidations are hampered or completely suppressed. Hence, a change of the synthetic strategy was necessary to first preform the HBC core, followed by the introduction of the porphyrins. All products were fully characterized, including, if possible, single‐crystal XRD. UV/Vis absorption spectra of porphyrin‐HBCs showed, depending on the number of porphyrins as well as with respect to the substitution pattern, variations in their spectral features with strong distortions of the porphyrins’ B‐band.

## Graphical abstract

**Hexabenzocoronenes** (HBCs), functionalized with up to six porphyrins, were prepared and studied. UV/Vis absorption spectroscopy showed variations in the spectral characteristics depending on the number of porphyrins as well as their respective substitution pattern. Insight into solid‐state behavior was obtained by X‐ray crystal structures.

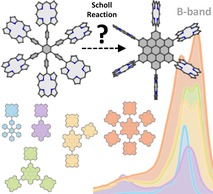

## Introduction

**Figure 1:**
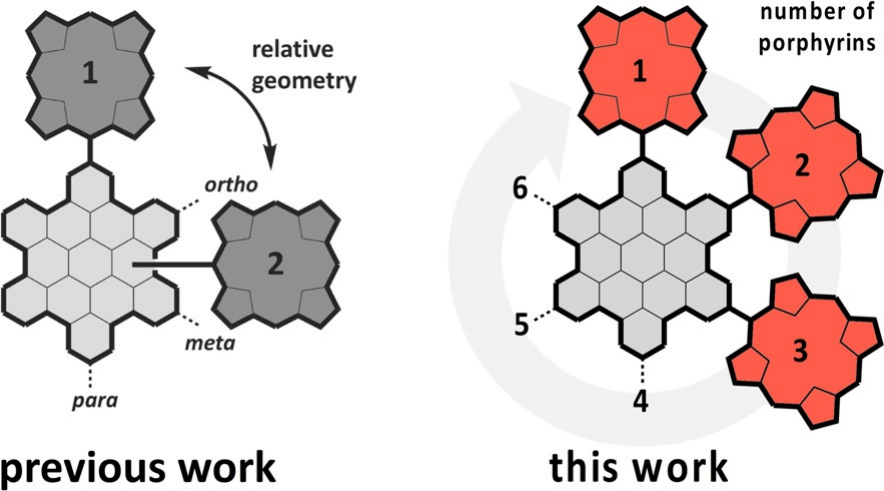
HBC as a model system for nanographenes with porphyrins directly attached to the periphery. Influence of the geometrical alignment (previous work, left side) and the amount (this work, right side) of porphyrins on the HBC core.

Hexaphenylbenzenes (HPBs) and their oxidized derivatives hexa‐*peri*‐hexabenzocoronenes (HBCs) have received tremendous attention during the past 20 years in materials chemistry, for example in molecular electronics and nonlinear optics.^1^, ^2^, ^3^, ^4^, ^5^ They have proven to be a suitable scaffold for catalytic applications,^6^, ^7^ the design of supramolecular architectures,^8^, ^9^ as well as a perfectly sized template for the preparation of nano‐ring^10^, ^11^ and nano‐ball^12^ structures. In contrast, porphyrins and related macrocycles, which are found in nature in different appearances such as heme, chlorophyll, or bacteriochlorophyll, have attracted the attention from the scientific community ever since their discovery. Due to their photophysical characteristics, featuring high extinction coefficients in the visible‐light region, porphyrins have proven to be an ideal chromophore for light‐harvesting architectures.^13^, ^14^, ^15^, ^16^, ^17^, ^18^, ^19^, ^20^, ^21^ The combination of both building blocks, porphyrins and HPBs, was initiated in 2002 by the synthesis of a HPB core, surrounded by six directly *meso*‐attached porphyrins.^22^ Subsequently, the compound class of porphyrin‐HPBs expanded to a library of extraordinary light‐harvesting arrays.^22^, ^23^, ^24^, ^25^, ^26^, ^27^, ^28^, ^29^, ^30^, ^31^ Their oxidized derivatives, porphyrin‐HBCs,^32^, ^33^, ^34^, ^35^, ^36^, ^37^, ^38^, ^39^, ^40^, ^41^ however, did not evolve parallel to the discoveries of novel porphyrin HPBs, although their preparation seems to be the logical continuation of the HPB architectures. The first directly linked porphyrin‐HBC was reported in 2013, it was synthesized in an intramolecular oxidative cyclodehydrogenation reaction of the respective porphyrin‐HPB.^33^ The transformation, commonly known as “Scholl reaction”, turns a flexible, nonconjugated HPB moiety into a planarized, aromatic HBC unit, which significantly influences the porphyrins’ characteristics. For example, due to an electronic coupling between the two π‐systems, a broadening and bathochromic shift of the UV/Vis absorption bands of the porphyrin was observed. In the recent years, compounds, in which one porphyrin is *meso*‐connected to several HBCs (up to four),^34^, ^35^, ^38^ to graphene nanoribbons^36^ or even fused to HBC units,^39^ were developed. However, systems in which one HBC is connected to several porphyrins have remained unknown until very recently. This year, our group reported the synthesis of a series of bis‐porphyrin‐HBC conjugates (Figure 1), which was utilized to study the effects of the HBCs’ substitution geometry.^40^ Furthermore, bis‐porphyrin‐substituted HBCs and extended HBC derivatives, were very recently prepared to investigate the influence of the nanographenes’ length.^41^ Given that these two studies^40^, ^41^ showed that both the substitution geometry and the size of the nanographene have a clear impact on the photophysical properties, we decided to further elucidate the characteristics of porphyrin–nanographene hybrid materials. Herein, we describe the synthesis of multiple‐porphyrin HBC conjugates with a functionalization degree of up to six porphyrins per HBC and study the effect of the number of porphyrins on the characteristics (Figure 1). For that purpose, HBCs substituted by several porphyrins were prepared by either Scholl transformations^33^, ^35^, ^38^ of respective porphyrin‐HPBs,^22^, ^24^, ^31^ or by Suzuki type cross‐coupling reactions between iodo‐HBCs and boronic ester porphyrins.^40^

## Results and Discussion

**Scheme 1:**
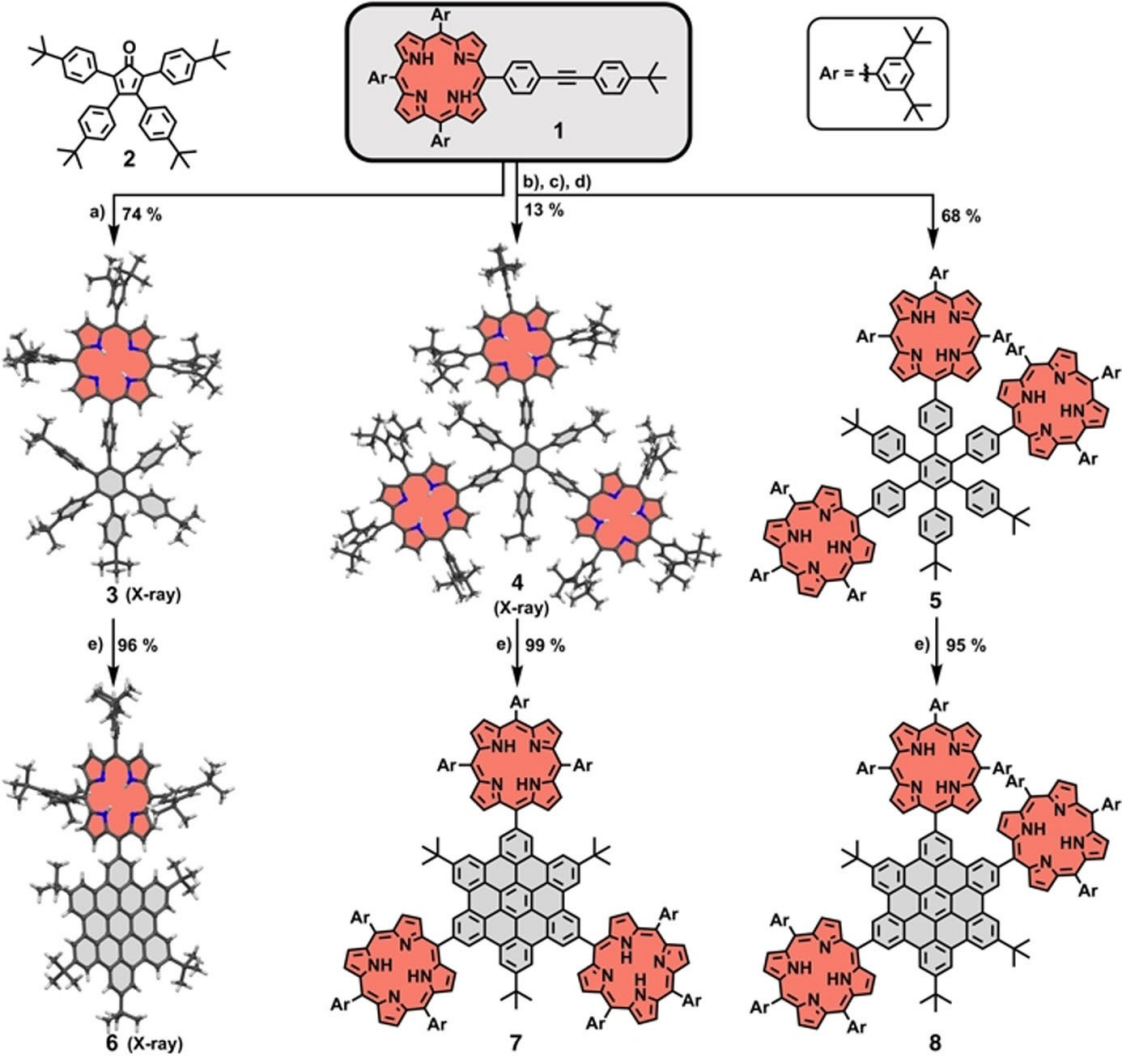
Synthesis of mono‐ and tri‐porphyrin‐HBCs **6**, **7**, and **8**. Molecules **3**, **4**, and **6** are depicted as X‐ray crystal structures (for details see the Supporting Information).^45^ a) Ph_2_O, 260 °C, μW; b) Ni(acac)_2_, toluene, 110 °C; c) Co_2_(CO)_8_, toluene, 110 °C; d) H_2_SO_4_, CH_2_Cl_2_, 0 °C; e) FeCl_3_, CH_3_NO_2_, CH_2_Cl_2_, 0 °C. Excess amounts of FeCl_3_ (29–41 equiv) were used for the Scholl oxidation as these conditions form the respective HBC derivatives efficiently within a relatively short amount of time (<24 h).

First, mono‐porphyrin‐HBC **6**,^33^, ^38^ previously made by us through a different route, was prepared as a reference compound (Scheme 1). For this purpose, mono‐porphyrin‐tolane **1** 31 was synthesized in a literature‐known statistical porphyrin condensation and reacted with tetracyclone **2** 40, 42 to the mono‐porphyrin‐HPB **3**.^31^, ^38^ The transformation to the respective HBC derivative **6** worked almost quantitatively using FeCl_3_ as the oxidant. Tolane‐porphyrin **1** is further suitable for the preparation of tri‐porphyrin‐substituted HPBs. Therefore, **1** was metallated with nickel and used in a cobalt‐catalyzed cyclotrimerization reaction, yielding the two isomers **4**⋅**Ni_3_**, and **5**⋅**Ni_3_**.^31^ After demetallation with conc. H_2_SO_4_, which facilitates purification and separation, **4** and **5** were obtained in 13 and 68 % yield, respectively. Isomers like **4**, with a 1,3,5‐substitution pattern, often form as the minor product during trimerization reactions due to statistical and electronic effects.^43^, ^44^ The Scholl oxidation to the respective tri‐porphyrin‐HBCs proceeded smoothly and both products **7** and **8** were obtained in excellent yields. Clearly, the presence of multiple porphyrins does not hamper Scholl oxidations in this case, regardless of the substitution pattern. All synthetic details are described in the Supporting Information.

**Scheme 2:**
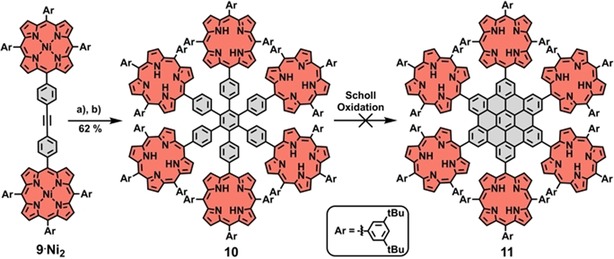
Planned synthetic route towards hexa‐porphyrin‐HBC **11** through cyclotrimerization and Scholl reaction. a) Co_2_(CO)_8_, toluene, 110 °C; b) H_2_SO_4_, CH_2_Cl_2_, 0 °C.

Next, we aimed to prepare a porphyrin‐saturated HBC compound, which is substituted by six porphyrins. For that, we planned to transform a hexa‐porphyrin‐substituted HPB **10**, which is well accessible and already known in the literature,^22^, ^29^, ^31^ to the respective HBC derivative **11** (Scheme 2). First, di‐porphyrin‐tolane **9**⋅**Ni_2_** 31 was prepared in a Sonogashira cross‐coupling reaction and subjected to a cobalt‐catalyzed cyclotrimerization reaction. Hexa‐porphyrin‐substituted HPB **10** was obtained, after demetallation, in 62 % yield. The final transformation to the target molecule hexa‐porphyrin‐HBC **11**, however, did not proceed as expected. The conditions we typically apply for Scholl oxidation (FeCl_3_/CH_3_NO_2_ in CH_2_Cl_2_), failed to deliver the central hexabenzocoronene. Instead, precursor **10** was recovered quantitatively. Variations of the reaction conditions (reaction time, amount of FeCl_3_, change of oxidant/Lewis acid) did not lead to any successful formation of the desired HBC product **11**.

**Scheme 3:**
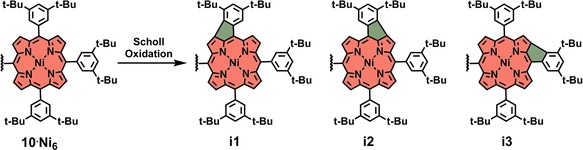
Suggested reaction products of Scholl oxidation with hexa‐nickel‐porphyrin‐HPB **10**⋅**Ni_6_**. The reaction is simplified by showing only one porphyrin of **10**⋅**Ni_6_**. For a single‐bond formation, up to three different isomers per porphyrin are feasible.

Although many different oxidant/Lewis acid combinations for Scholl oxidations exist^46^, ^47^ and the mechanism is still not fully understood,^48^, ^49^, ^50^, ^51^, ^52^ we suspect that the reason behind the failure of HBC formation of **10** stems from its structure. On the one hand, the HPB core is sterically well shielded due to the arrangement of the bulky porphyrins around it. Additionally, given that the porphyrins are in their free‐base form, protonation occurs under the acidic reaction conditions, leading to positively charged porphyrin‐HPB conjugates. Coulombic repulsion between the charged porphyrins and the oxidant Fe^3+^ further hampers the reactivity. To avoid electrostatic effects, we introduced metals to the porphyrin core. First, zinc was tested and hexa‐zinc‐porphyrin‐HPB **10**⋅**Zn_6_** was prepared and reacted in a Scholl oxidation. However, zinc did not endure the acidic reaction conditions and therefore, demetallation occurred as the only reaction,^35^, ^40^ yielding free‐base porphyrin‐HPB **10** as the product. Given that more robust metal complexes were required, nickel was inserted into the porphyrins. Hexa‐nickel‐porphyrin‐HPB **10**⋅**Ni_6_** was subjected to the conditions used for Scholl reactions and in contrast to all previous attempts, reactivity was observed and new product spots were detected by TLC. However, rather than forming the desired HBC product, it seemed that the peripheral porphyrins have reacted with their *meso*‐3,5‐di‐*tert*‐butylphenyl substituents (Scheme 3), as it has already been shown for other 3,5‐di‐*tert*‐butylphenyl‐substituted nickel porphyrins.^53^, ^54^, ^55^ Owing to the possibility of forming up to three different isomers **i1**, **i2**, **i3** per porphyrin, an inseparable mixture of products was obtained, which showed neither in NMR nor in UV/Vis spectroscopy indications of an HBC formation (Figures S40 and S41, Supporting Information). All reaction conditions, which were tested to initiate the Scholl oxidation, are summarized in Table 1. To get a better understanding about the failure of HBC formation of **10**, calculations were performed (see Figure S6). From a theoretical perspective, HBC formation seems to be unlikely with hexa‐porphyrin‐HPB **10**, because most of the highest‐occupied molecular orbital (HOMO) electron density is located on the porphyrins and only little on the HPB unit. Thus, the preferred point of oxidation is rather at the porphyrins than at the HPB.

**Table 1:** Scholl reaction conditions, which were tested to prepare hexa‐porphyrin‐HBC **11**.

Molecule	Reaction conditions	Reaction type	Product
**10**	FeCl_3_ (118 equiv)	/	**10** (recovered)
**10**	DDQ (9 equiv) triflic acid (24 equiv)	/	**10** (recovered)
**10⋅Zn_6_**	FeCl_3_ (119 equiv)	demetallation	**10**
**10⋅Ni_6_**	FeCl_3_ (125 equiv)	fusion of *meso* aryl substituents to porphyrin	mixture of isomers

**Scheme 4:**
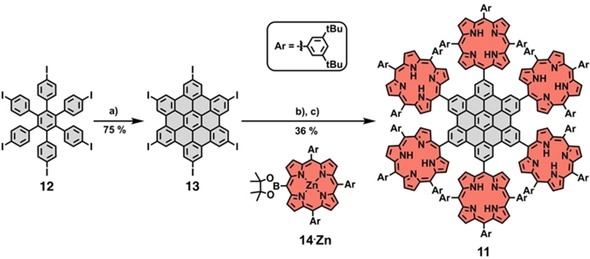
Successful synthesis of hexa‐porphyrin‐HBC **11** through Suzuki reaction. a) FeCl_3_, CH_3_NO_2_, CH_2_Cl_2_, 0 °C; b) Pd(PPh_3_)_4_, Cs_2_CO_3_, toluene, DMF, 80 °C; c) trifluoroacetic acid, CHCl_3_.

Given that transformations of hexa‐porphyrin‐HPB **10** to the corresponding HBC could not be achieved, a new strategy was developed in which the HBC was preformed before the attachment of the porphyrins (Scheme 4). Therefore, hexa‐iodo‐HPB **12** 56, 57 was prepared and oxidized to the HBC derivative **13**.^58^, ^59^ Then, HBC **13** was reacted with the boronic ester porphyrin^40^, ^60^ **14**⋅**Zn** in a six‐fold Suzuki reaction to the desired product **11**. In spite of the spatially restricted situation of the product and the very low solubility of hexa‐iodo‐HBC **13**, the six‐fold Suzuki reaction worked sufficiently and produced hexa‐porphyrin‐HBC **11**, after demetallation, in 36 % yield.

**Figure 2:**
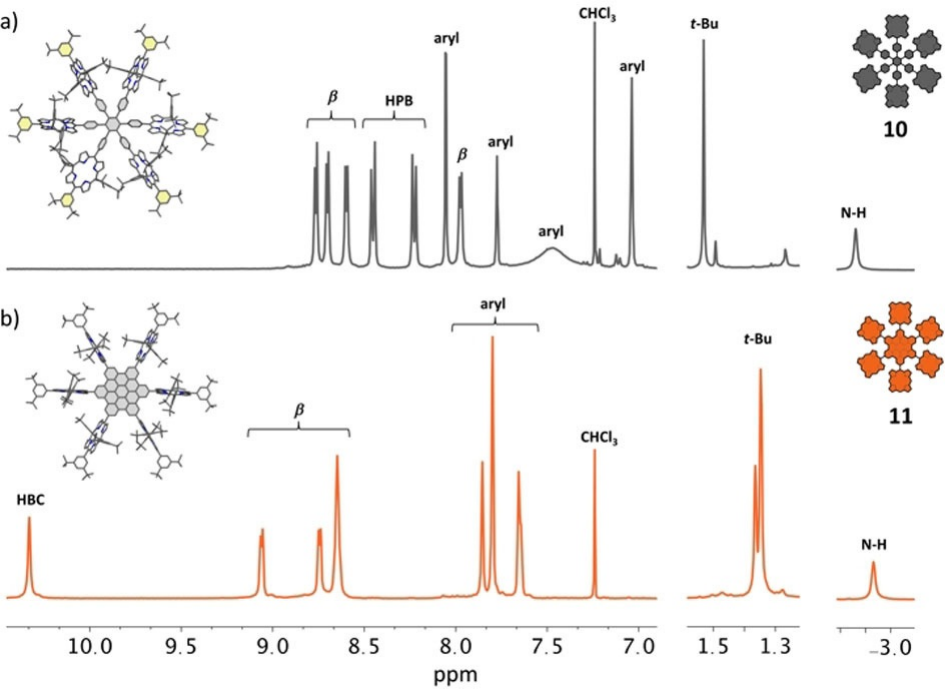
^1^H NMR (400 MHz, CDCl_3_, rt) of hexa‐porphyrin‐HPB **10** (top) and hexa‐porphyrin‐HBC **11** (bottom). Calculated structures (semi empirical, PM6) are depicted; hydrogen atoms are omitted for clarity.

The successful formation of hexa‐porphyrin‐HBC **11** was verified by NMR spectroscopy, which shows clear differences between the HPB and HBC species (see ^1^H NMR, Figure 2). Characteristic for directly linked porphyrin‐HBC compounds^33^, ^34^, ^35^, ^38^, ^40^ are the most downfield‐shifted signals around 10 ppm, which originate from the HBC protons close to the porphyrin. Due to the high symmetry of **11** (*D*_6*h*_) the NMR spectrum is rather simple for a molecule with a molar mass *M*=5762 Da. The proton signal for the HBC core for example, is only noticeable as one singlet at 10.33 ppm. A comparison between the spectra of **10** and **11** shows that the resonances of the protons close to the HPB/HBC core experience the strongest chemical shifts. For example, signals of the β‐pyrrolic protons of HPB **10** show up at 7.97 ppm whereas the most downfield‐shifted ones of the HBC derivative are found at 9.06 ppm. Furthermore, for HPB system **10**, the resonances of the porphyrins’ outwards pointing aryl rings (Figure 2, drawn in yellow) appear sharp whereas the signals of the other aryl rings are broadened.^22^ This is also true for the *tert*‐butyl groups, which almost vanish. The broadening of some signals is due to the sterically stressed situation in **10** resulting in restricted rotational freedom.^22^ For the HBC derivative **11**, on the other hand, the rigid HBC core results in sharp appearance of all signals.

**Figure 3:**
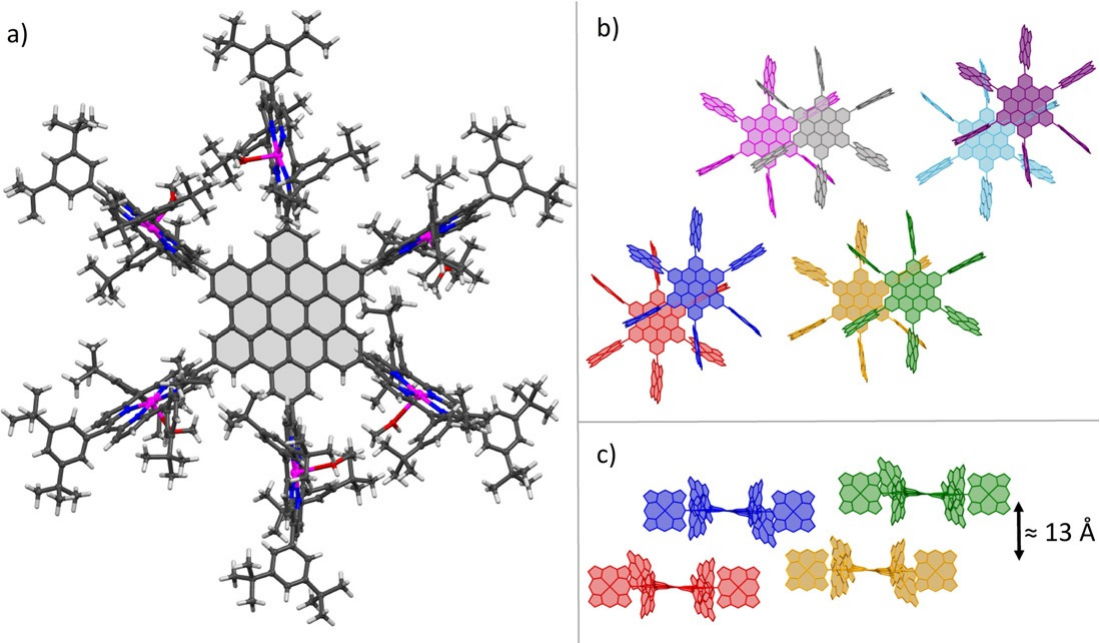
a) Structural motif of hexa‐zinc‐porphyrin‐HBC **11**⋅**Zn_6_** with MeOH coordinated to each central zinc atom; ^[32]^ b) top view of eight molecules of **11**⋅**Zn_6_**; c) side view of four molecules of **11**⋅**Zn_6_**; b), c) 3,5‐di‐*tert*‐butylphenyl groups and hydrogen atoms as well as disordered groups are omitted for clarity.

**Figure 4:**
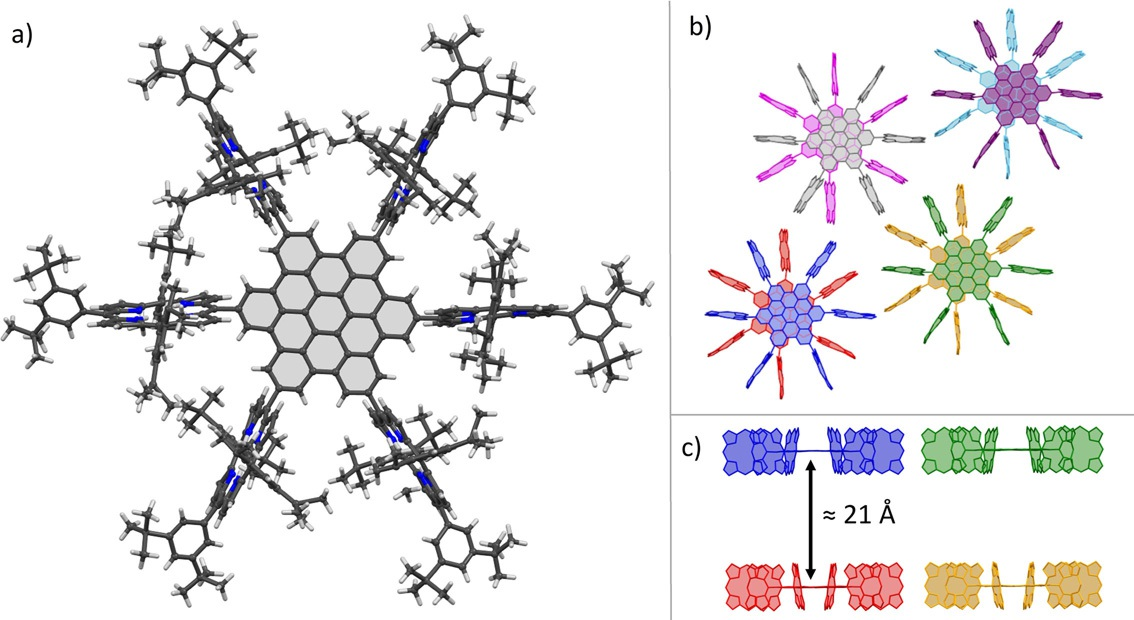
a) Structural motif of hexa‐free‐base‐porphyrin‐HBC **11**; b) top view of eight molecules of **11**; c) side view of four molecules of **11**; b), c) 3,5‐di‐*tert*‐butylphenyl groups and hydrogen atoms as well as disordered groups are omitted for clarity.

X‐ray diffraction analysis (XRD) with single crystals of **11** and **11**⋅**Zn_6_** turned out to be challenging. Due to the size of the molecules with the chemical composition of C_414_H_450_N_24_ (**11**) and C_414_H_438_N_24_Zn_6_ (**11**⋅**Zn_6_**) and the therefore expected large unit cells, only weak diffraction patterns were obtained. To get sufficient crystallographic data, either high X‐ray intensities or large crystals were required. Given that the crystallization attempts of zinc‐porphyrin‐HBC **11**⋅**Zn_6_** generated only small crystals, synchrotron X‐ray intensities^61^ were required to obtain suitable crystallographic data (Figure 3). Molecules **11**⋅**Zn_6_** are arranged in a monoclinic crystal system with a unit‐cell volume of approximately 53 000 Å^3^. Solvent molecules incorporated into the crystal were highly disordered and therefore removed during refinement. However, MeOH, which was used as the anti‐solvent, was properly modelled for each porphyrin because it is coordinated to the central zinc ion. Crystallization attempts of the free‐base form of **11**, in contrast, yielded large crystals (0.7×0.5×0.5 mm^3^), which were suitable for measurements on a standard X‐ray diffractometer (Figure 4).^62^ In this case, the molecules are oriented in a hexagonal crystal system with a unit‐cell volume of approximately 130 000 Å^3^. A comparison of the crystal packing shows a difference in solid‐state arrangement of molecules **11** and **11**⋅**Zn_6_**, respectively, with an interlocked packing motif for the zinc derivative (Figure 3 b, c) and a columnar arrangement for the free‐base form (Figure 4 b, c). Although the datasets of both structures are not suitable for detailed structural discussions, they unambiguously verify the successful formation of the desired hexa‐porphyrin‐substituted HBCs **11** and **11**⋅**Zn_6_**.

**Figure 5:**
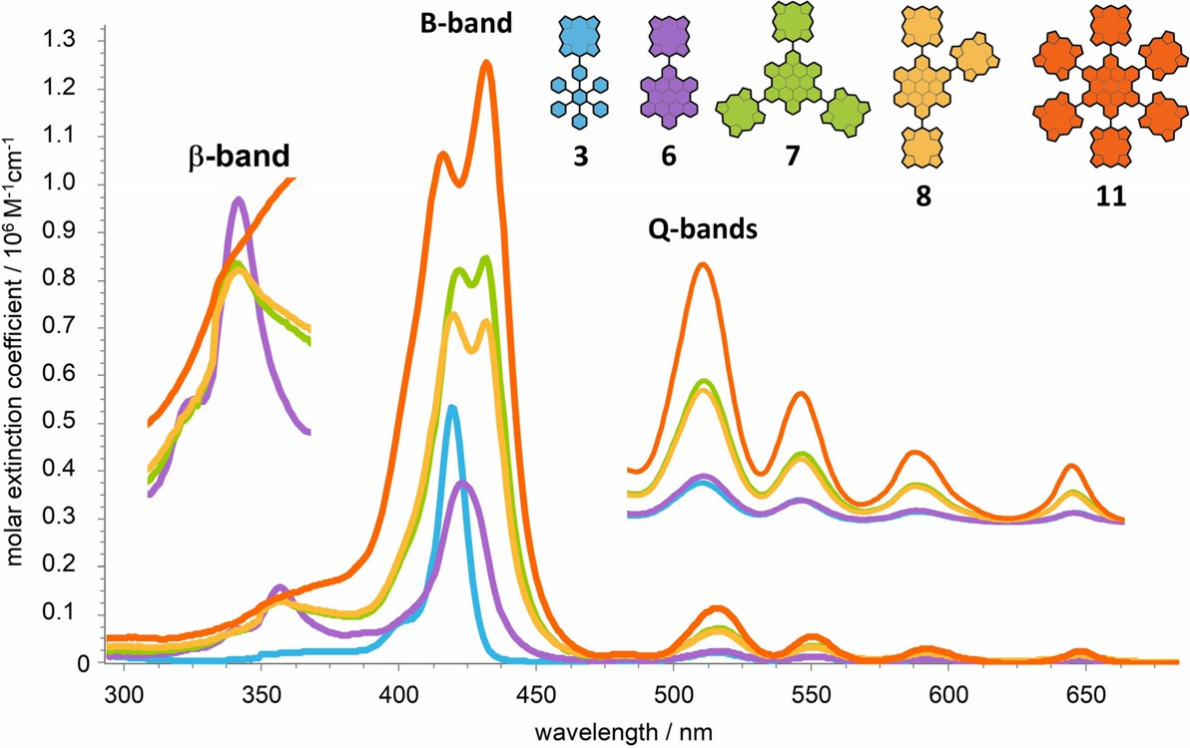
UV/Vis absorption spectra^63^ of molecules **3**, **6**, **7**, **8**, and **11** in THF. Inserts show magnifications of the β‐ (left side) and Q‐band (right side) absorptions.

UV/Vis absorption spectra were recorded for all porphyrin‐HPB and HBC compounds and showed variations in their spectral features. Depending on the number of porphyrins, their substitution pattern, and core unit (HPB or HBC), different photophysical characteristics were observed. For the hexaphenylbenzene linked conjugates **3**, **4**, **5**, and **10** the B‐band (Soret band) absorptions appear as sharp signals with full width at half maximum (FWHM) values of 11.6–13.8 nm and molar extinction coefficients per porphyrin *ϵ*_B‐band_/*n*(porphyrin) of 4.00–5.00⋅10^5^ m ^−1^ cm^−1^.^31^ The transformation to the corresponding porphyrin‐HBCs, significantly changes the photophysical properties.^33^, ^38^ As an example, the spectra of mono‐porphyrin‐HPB **3** and HBC **6** are depicted in Figure 5 (blue and purple lines). The B‐band of mono‐porphyrin‐HBC **6** is redshifted, decreased in intensity, and significantly broadened with respect to the HPB precursor **3** (see Table 2). Furthermore, an additional absorption band, originating from the newly formed HBCs’ π‐system, arises at 357 nm. A comparison of the UV/Vis absorption features of hexabenzocoronene centered compounds **6**, **7**, **8**, **11** (Figure 5) shows that the number of porphyrins and the substitution pattern conspicuously influence the spectral properties. Compared with the HPB analogues, the porphyrins’ B‐band is split and considerably broadened, if more than one porphyrin is attached to the HBC core. Similar characteristics were observed for recently reported bis‐porphyrin‐substituted HBCs,^40^ which showed, depending on the substitution geometry, split B‐band absorptions as well (Figure S11, Supporting Information). Interestingly, the right (lower‐energy) maximum is always located at the same position, 432 nm, for **7**, **8**, **11**, as well as for *para*‐bis‐porphyrin‐HBC^40^ and only the left (higher‐energy) maximum is shifted. The position of the Q‐bands, and therefore the optical band gap, remains unaffected by the number of porphyrins. The intensity of the HBCs’ β‐absorption, however, is strongly dependent on the number of porphyrins. The most pronounced β‐band was found for mono‐porphyrin‐HBC **6**, whereas for tri‐porphyrin‐HBCs **7** and **8** a decreased, and for hexa‐porphyrin‐HBC **11**, no distinct maximum, due the superposition with the porphyrins’ absorption, were observed.

**Table 2:** Spectroscopic data for porphyrin‐HPB/HBC conjugates.

Molecules A=*tert‐*butyl B=porph.	Soret band λ [nm] (*ϵ* [10^5^ m ^−1^ cm^−1^])	*ϵ*_Soret_/*n*(porph.) [10^5^ m ^−1^ cm^−1^]	FWHM Soret [nm]	Fluorescence *λ*_exc._: β‐band *λ*_emi._ [nm ] (rel. int.)	Fluorescence *λ*_exc._: Soret_max_ *λ*_emi._ [nm] (rel. int.)
A_5_B	419 (5.03)	5.03	11.6	/	653 (1.00)
HPB **3**					719 (0.21)
A_5_B	423 (3.64)	3.64	20.7	653 (0.43)	652 (1.00)
HBC **6**				718 (0.11)	717 (0.23)
(AB)_3_	422 (7.84)	2.61	28.0	653 (0.22)	652 (1.00)
HBC **7**	432 (8.08)	2.69		714 (0.05)	718 (0.23)
A_2_B_2_AB	420 (7.22)	2.41	30.7	652 (0.19)	652 (1.00)
HBC **8**	432 (7.11)	2.37		717 (0.05)	717 (0.23)
B_6_	416 (10.4)	1.73	37.4	650 (0.23)	650 (1.00)
HBC **11**	432 (12.2)	2.03		716 (0.05)	716 (0.23)

The spectral variations of porphyrin‐HBCs compared with the HPB‐linked derivatives are much more pronounced, due to the HBCs’ conjugated π‐system, which is in contrast to the flexible HPB bridge, more effective in elongating the π‐conjugation pathway. On the one hand, the large aromatic π‐system of the HBC itself influences the porphyrins’ electronic characteristics (compare mono‐porphyrin HPB **3** vs. HBC **6**) and on the other hand, the HBC unit facilitates electronic interaction between the porphyrins. In our study with bis‐porphyrin substituted HBCs^40^ we already investigated the communication ability across HBC bridges and suggested that the degree of the B‐bands’ distortion can be used as a qualitative tool to measure the electronic interaction between the porphyrins.^40^ The herein presented porphyrin‐HBCs follow this trend, for example, tri‐porphyrin substituted HBC **8** has a broader (FWHM 30.7 nm) and more split (12 nm) B‐band absorption as the isomer **7**. This is because two of the porphyrins in **8** are arranged in a *para* geometry, allowing the best interaction,^40^ whereas in **7** all of the porphyrins are aligned in a *meta* fashion. The number of porphyrins per HBC has also a significant influence on photophysical properties. A comparison between porphyrin‐HBCs **6**, **7**, **8**, **11** as well as bis‐porphyrin‐HBCs^40^ (Figure 5, Figure S11, Supporting Information), shows that an increasing number of porphyrins per HBC leads to a higher degree of B‐band distortion, hence enhanced communication between the porphyrins. Thus, the strongest electronic interaction was found for hexa‐porphyrin‐HBC **11** with a split in the Soret‐band of 16 nm and a FWHM value of 37.4 nm. Steady‐state fluorescence spectra were measured (Figure S12, Supporting Information) and showed upon excitation of the HBCs’ β‐band an efficient energy transfer to the porphyrins yielding only their fluorescence at 653 and 718 nm.^33^, ^34^, ^35^, ^38^ Generally, the influence of substitution pattern and number of porphyrins on the steady‐state fluorescence characteristics were less pronounced compared with the changes of the UV/Vis absorption properties. All spectroscopic data are summarized in Table 2.

## Conclusions

Porphyrin‐HBC conjugates bearing one and three porphyrins per HBC were prepared through Scholl oxidation reactions of the respective HPB precursors. With respect to previously reported bis‐porphyrin‐HBCs, we come to the conclusion that Scholl transformations are an excellent, high yielding choice for the preparation of porphyrin‐HBCs with up to three porphyrins per molecule. Clearly, effects that might hinder Scholl reactions, such as steric shielding or electrostatic repulsion, are less pronounced and therefore do not influence the reaction outcome if not more than three porphyrins are attached to a central HPB core. However, the synthesis of higher substituted porphyrin‐HBCs through this synthetic route is not advisable, because the reactivity of the respective precursors under typical Scholl reaction conditions is hampered. The lack of reactivity was clearly demonstrated by the attempts to transform hexa‐porphyrin‐HPB **10** into the HBC compound **11**. Several reaction conditions, as well as different metalation states of the porphyrins, were tested and all of them failed to form the HBC core of **11**. Preliminary results further suggest, that already tetra‐porphyrin substituted HPBs have a decreased reactivity, making the transformation to the respective HBC derivatives inefficient.^64^ Given that hexa‐porphyrin‐HBC **11** could not be obtained through a Scholl reaction‐based route, the synthetic strategy was changed to the preparation of the HBC core prior to the porphyrins’ introduction. The spectroscopic data of porphyrin‐HBCs within this project further complements the series of multiple‐porphyrin‐substituted HBCs.^40^ UV/Vis absorption spectra showed that an increased number of porphyrins per HBC leads to a higher degree of the B‐bands’ distortion, which was attributed to an enhanced electronic communication between the porphyrins. With respect to the design and the characteristics of light‐harvesting arrays, we conclude that unlike in nonconjugated HPB architectures, the electronic communication across a large π‐system (HBC) significantly changes with the substitution pattern and the number of chromophores, which is reflected in the FWHM values of the Soret band of the porphyrins. Although the broadened and split B‐band of multiple‐porphyrin‐HBCs can be ascribed to intramolecular electronic interactions, detailed understanding of the photophysical properties is still lacking and therefore a topic of current investigations. Additionally, compounds like hexa‐porphyrin‐HBC **11** are tested for the buildup of self‐assembled supramolecular architectures with guest molecules such as C_60_‐fullerene.

## Experimental Section

Experimental procedures, characterization data, X‐ray crystallographic data and copies of HRMS and NMR spectra can be found in the Supporting Information.

## Conflict of interest

The authors declare no conflict of interest.

## Supporting information

As a service to our authors and readers, this journal provides supporting information supplied by the authors. Such materials are peer reviewed and may be re‐organized for online delivery, but are not copy‐edited or typeset. Technical support issues arising from supporting information (other than missing files) should be addressed to the authors.

Supplementary
