# Supplementary material for: Multiple‐Porphyrin Functionalized Hexabenzocoronenes
Source: Chemistry. 2019 Oct 22;25(66):15083–90. doi: 10.1002/chem.201903113 (PMC6899994; doi:10.1002/chem.201903113)
Supplement: Supplementary file 1 — Supplementary [file CHEM-25-15083-s001.pdf]

CHEMISTRY
A European Journal

Supporting Information

Multiple-Porphyrin Functionalized Hexabenzocoronenes

Max M. Martin,[a] Dominik Lungerich,[a] Frank Hampel,[a] Jens Langer,*[b] Tanya K. Ronson,[c] and Norbert Jux*[a]

chem_201903113_sm_miscellaneous_information.pdf

Table of Content

1 General Information S2

2 Experimental Section S3

3 Crystallographic Data S21

4 Calculations S34

5 Spectral Appendix S37

6 References S58

1 General Information

All chemicals were purchased from Sigma-Aldrich and used without any further purification. Solvents were distilled prior to usage. Dichloromethane and chloroform were neutralized with K_2CO_3 before distillation. Thin layer chromatography (TLC) was performed on Merck silica gel 60 F524, detected by UV-light (254 nm, 366 nm). Column chromatography and flash column chromatography were performed on Macherey-Nagel silica gel 60 M (deactivated, 230-400 mesh, 0.04 –0.063 mm). NMR spectroscopy was performed on JEOL JNM EX 400 (^{1}H: 400 MHz, ^{13}C: 101 MHz), JEOL Alpha 500 (^{1}H: 500 MHz, ^{13}C: 126 MHz) and Bruker Avance 400 (^{1}H: 400 MHz, ^{13}C: 101 MHz). Deuterated solvents were purchased from Sigma Aldrich and used as received. Chemical shifts are referenced to residual protic impurities in the solvents (^{1}H: $CHCl_3$: 7.24 ppm, $CHDCl_2$: 5.32 ppm) or the deuterated solvent itself (^{13}C: $CDCl_3$: 77.0 ppm, CD_2Cl_2: 53.8 ppm). The resonance multiplicities are indicated as "s" (singlet), "d" (doublet), "t" (triplet), "q" (quartet) and "m" (multiplet). Signals referred to as "bs" (broad singlet) are not clearly resolved or significantly broadened. LDI/MALDI-ToF mass spectrometry was performed either on a Shimazu AXIMA Confidence or a Bruker Ultraflex Extreme machine. In case of MALDI, the following matrix were used: 2,5-dihydroxybenzoic acid (DHB), sinapic acid (SIN) or *trans*-2-[3-(4-*tert*-butylphenyl)-2-methyl-2-propenylidene]malononitrile (DCTB). High resolution mass spectrometry was performed on a ESI/APPI-ToF mass spectrometer Bruker maXis 4G UHR MS/MS spectrometer or a Bruker micrOTOF II focus TOF MS-spectrometer. Microwave reactions were carried out in a mono-mode microwave reactor Biotage Initiator^{+}. The microwave assisted reactions were carried out exclusively in the fixed hold time mode using an external IR temperature sensor. UV/Vis spectroscopy was carried out on a Varian Cary 5000 UV-Vis-NIR spectrometer. Unless otherwise noted, reactions were degassed by the following technique: The reaction mixture was sonicated at 25 °C for 1 min under vacuo, followed by a purge with N_2-gas. This cycle was repeated three times.

2 Experimental Section

4-((4-(*Tert*-butyl)phenyl)ethynyl)benzaldehyde[1] and 2,3,4,5-tetrakis(4-(*tert*-butyl)phenyl)-cyclopenta-2,4-dien-1-one[2-3] **2** were prepared according to literature procedures reported by our group. Dipyrromethane was prepared following a procedure of Anderson *et al.*[4] and 3,5-di-*tert*-butylphenyl-dipyrromethane was prepared according to Schuster *et al.*[5]

2.1 Synthesis of porphyrin precursors

The synthesis of porphyrins **1**, **1·Ni**, **15, 15·Ni**, **16**, **16·Ni**, **17·Ni**, **9·Ni$_2$** was performed according to the previously reported protocol.[1]

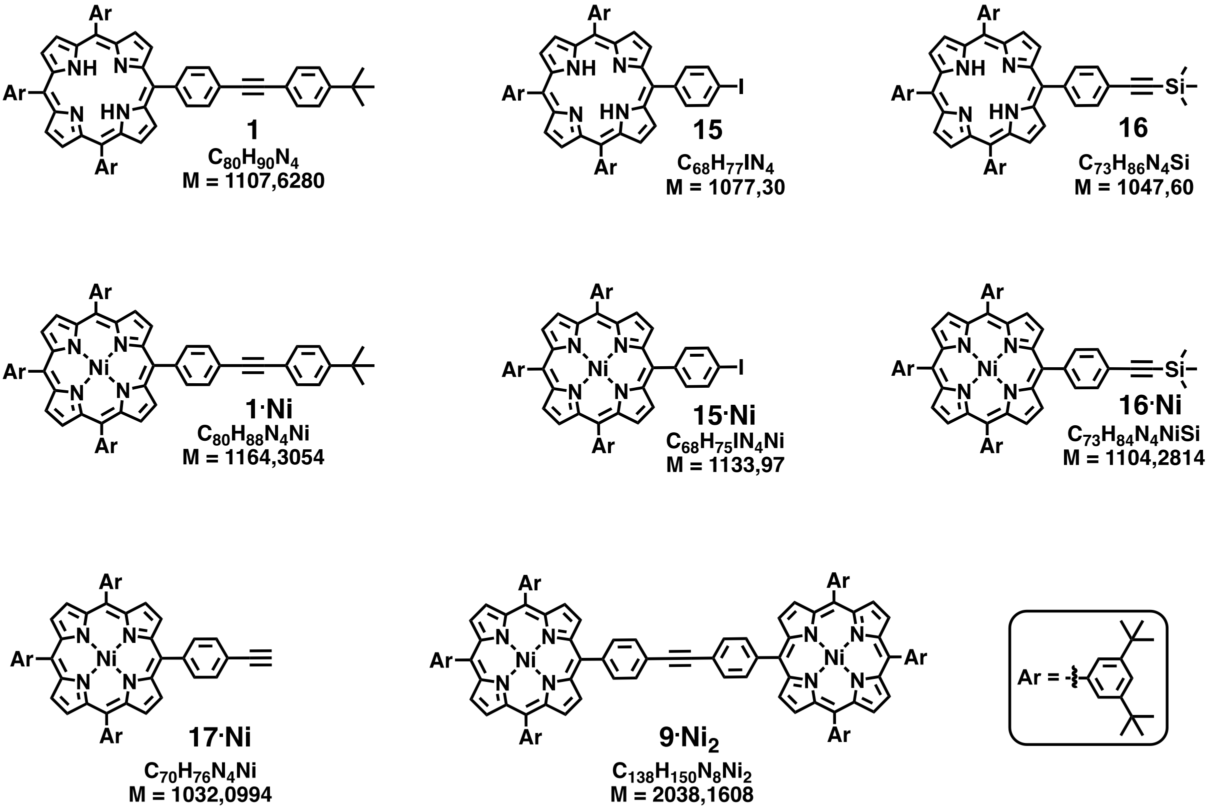

General procedure for the synthesis of A_3B porphyrins.[1] A 20 mL microwave vial was charged with CH_2Cl_2 (19 mL), **aldehyde A** (1.50 mmol), **aldehyde B** (0.50 mmol) and pyrrole (140 µl, 2.00 mmol). A solution of I_2 (25 mg, 0.10 mmol) in CH_2Cl_2 (1.0 mL) was added to the vial, it was sealed with a septum and heated in the microwave reactor (20 s pre stirring, 5 min at 40 °C, max. power 100 W). A flask charged with *para*-chloranil (368 mg, 1.50 mmol) in CH_2Cl_2 (10 mL) was prepared and heated to reflux. After the microwave reaction has finished, the reaction mixture was poured into the *para*-chloranil solution and stirred for 15 min at reflux. The reaction was performed two times. The combined product mixture was adsorbed on SiO_2 (approx. 10 g) and separated via silica column chromatography (hexanes/CH_2Cl_2 – 3:1, Ø 6 cm · 37 cm). The second fraction was identified as the desired A_3B product.
Yields: 13 – 15 %.

General procedure for the synthesis of nickel porphyrins.[1] Free base porphyrin (1 equiv), $Ni(acac)_2$ (5 equiv) were dissolved in toluene (25 – 50 mL) and heated to reflux for 90 min. The solvent was removed and the product purified by silica plug filtration (hexanes/CH_2Cl_2 – 1:1). The product was obtained as a red-orange solid.

Nickel-ethynylporphyrin 17·Ni.[1] TMS protected nickel-porphyrin **16·Ni** (137 mg, 124 µmol) was dissolved in THF (15 mL), a 1 M TBAF solution in THF (300 µL, 300 µmol) was added and the reaction was stirred light protected for 90 min at rt. The solvent was removed and the porphyrin was purified by silica plug filtration (hexanes/CH_2Cl_2 – 4:1). The product could be obtained in 78.1 % yield (100 mg, 96.9 µmol).

Nickel-porphyrin dimer 9·Ni₂.[1] A 20 mL vial was charged with nickel-iodo-porphyrin **15·Ni** (95.5 mg, 84.3 µmol, 1 equiv), $Pd(PPh_3)_2Cl_2$ (3.0 mg, 4.3 µmol, 0.05 equiv), CuI (1.6 mg, 8.4 µmol, 0.1 equiv), NEt_3 (2.5 mL) and THF (2.5 mL). The vial was sealed with a septum, the mixture degassed and stirred for 10 min at rt before a degassed solution of-nickel ethynyl-porphyrin **17·Ni** (100 mg, 96.9 µmol, 1.15 equiv) in THF (2.5 mL) was added via syringe through the septum. The reaction was heated to 50 °C for 20 h under the exclusion of light. The solvent was removed and the crude purified by column chromatography (SiO_2, hexanes/CH_2Cl_2 – 4:1). The product was obtained as a red solid in 95.5 % yield (164 mg, 80.5 µmol).

2.2 Synthesis of mono-porphyrin-HBC 6

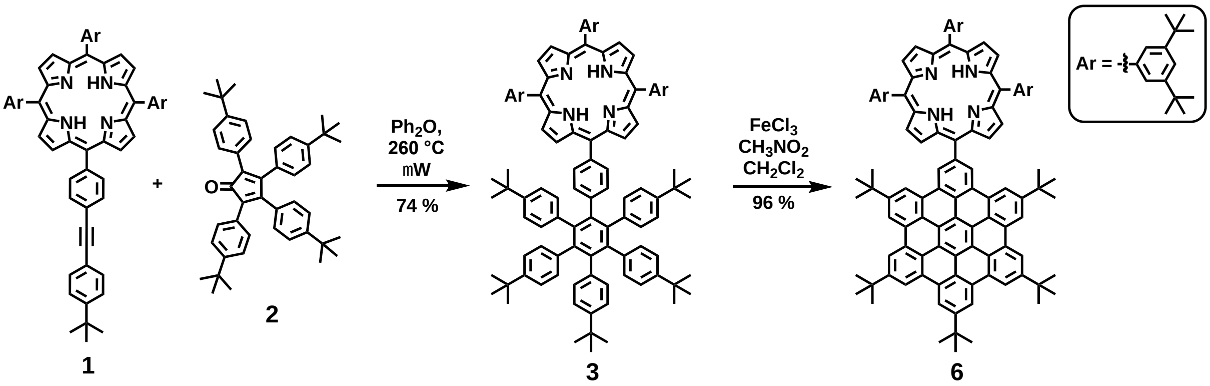

Scheme S1. Synthesis of mono-porphyrin-HBC **6**.

Diels Alder reaction: Mono-Porphyrin-HPB 3

Free base tolane-porphyrin **1** (55 mg, 49.3 μmol, 1 equiv) and *tert*-butyl substituted tetracyclone **2** (60 mg, 98.5 μmol, 2 equiv) were dissolved in Ph_2O (1.0 mL), purged with argon and heated to 260 °C for 12h in the microwave reactor. The crude was diluted with little CH_2Cl_2 and the product was precipitated via addition of MeOH (20 mL). The solid was filtered off and washed with MeOH (4 x 5 mL). The pure product could be obtained after column chromatography (SiO_2, hexanes/CH_2Cl_2 - 3:1, ∅ 4.5 cm · 33 cm) as a purple solid in 74.4 % yield (62 mg, 36.7 μmol).

^{1}H NMR (400 MHz, $CDCl_3$, rt): δ [ppm] = 8.91 (d, *J* = 4.8 Hz, 2H), 8.87 (d, *J* = 4.8 Hz, 2H), 8.71 (d, *J* = 4.8 Hz, 2H), 8.45 (d, *J* = 4.7 Hz, 2H), 8.05 (d, *J* = 1.7 Hz, 6H), 7.78 (t, *J* = 1.8 Hz, 2H), 7.77 (t, *J* = 1.8 Hz, 1H), 7.69 (d, *J* = 8.0 Hz, 2H), 7.19 (d, *J* = 8.1 Hz, 2H), 7.14 (d, *J* = 8.4 Hz, 4H), 7.01 (d, *J* = 8.3 Hz, 4H), 6.93 – 6.78 (m, 12H), 1.52 (s, 36H), 1.50 (s, 18H), 1.32 (s, 18H), 1.15 (s, 18H), 1.14 (s, 9H), -2.80 (s, 2H).

^{13}C NMR (101 MHz, $CDCl_3$, rt): δ [ppm] = 148.74, 148.68, 148.1, 147.7, 147.6, 141.5, 141.2, 141.1, 140.9, 140.6, 140.44, 140.39, 138.7, 138.2, 138.0, 137.9, 132.7, 131.6, 131.3, 131.2, 130.0, 129.8, 129.7, 123.5, 123.24, 123.21, 121.22, 121.20, 120.9, 120.1, 34.96, 34.95, 34.3, 34.06, 34.04, 31.7, 31.4, 31.2.

UV/Vis (THF): λ [nm] (ε [$M^{-1}cm^{-1}$]) = 419 (503000), 516 (18900), 551 (10900), 593 (5530), 648 (5130).

Fluorescence (THF): $\lambda_{exc.}$ [nm] = 419, **$\lambda_{emission}$ [nm] (rel. int.)** = 653 (1.00), 719 (0.21).

MS (LDI): m/z (rel. int.) = 1687.01 (M^+, 100 %).

HRMS (APPI, toluene) for $C_{124}H_{142}N_4$ (M^+), calc.: 1688.1262, found: 1688.1281.

Spectroscopic data is in good agreement with the literature (synthesis via mixed cyclotrimerization reaction).[1]

Scholl Oxidation: Mono-Porphyrin-HBC 6

Modified procedure from Jux *et al.*[6]

A 50 mL Schlenk tube, filled with porphyrin-HPB **3** (47 mg, 27.8 μmol, 1 equiv) and CH_2Cl_2 (30 mL), was cooled with an ice bath. The solution was degassed (N_2 bubbling through the solution for 15 min). The N_2 flow was increased and a solution of dry $FeCl_3$ (130 mg, 0.80 mmol, 30 equiv) in CH_3NO_2 (0.40 mL) was added. The N_2 flow was stopped 15 min after $FeCl_3$ was added and the solution was stirred under slow warming to rt for 8 h. The reaction was quenched via the addition of MeOH (10 mL). After removing the solvent, the crude was dissolved in little CH_2Cl_2. NEt_3 (1 mL) was added and the product was purified by silica plug filtration (SiO_2, CH_2Cl_2). The pure product was obtained in 96.4 % yield (45 mg, 26.8 μmol).

^{1}H NMR (400 MHz, $CDCl_3$, rt): δ [ppm] = 10.14 (s, 2H), 9.38 (s, 4H), 9.37 (s, 2H), 9.36 (s, 2H), 9.31 (s, 2H), 9.07 (d, *J* = 4.8 Hz, 2H), 8.97 (bs, 6H), 8.15 (bs, 6H), 7.82 (t, *J* = 1.8 Hz, 1H), 7.77 (t, *J* = 1.8 Hz, 2H), 1.87 (s, 9H), 1.85 (s, 18H), 1.60 (s, 18H), 1.55 (s, 18H), 1.51 (s, 36H), -2.45 (s, 2H).

^{13}C NMR (101 MHz, $CDCl_3$, rt): δ [ppm] = 149.5, 149.4, 149.3, 148.83, 148.79, 141.4, 141.3, 140.9, 130.74, 130.68, 130.65, 130.4, 130.0, 129.9, 129.2, 127.7, 125.54, 124.56, 124.14, 124.09, 121.84, 121.79, 121.12, 121.09, 120.9, 120.7, 119.9, 119.7, 119.4, 119.13, 119.07, 35.74, 35.72, 35.65, 35.02, 34.98, 32.00, 31.99, 31.8, 31.72, 31.67.

UV/Vis (THF): λ [nm] (ε [$M^{-1}cm^{-1}$]) = 357 (151000), 423 (364000), 516 (22800), 551 (11000), 591 (6380), 648 (5270).

Fluorescence (THF): **$\lambda_{exc.}$ [nm]** = 357, **$\lambda_{emission}$ [nm] (rel. int.)** = 653 (0.43), 718 (0.11).
$\lambda_{exc.}$ [nm] = 423, **$\lambda_{emission}$ [nm] (rel. int.)** = 652 (1.00), 717 (0.23).

MS (MALDI, DCTB): m/z (rel. int.) = 1676.11 (M^+, 100 %).

HRMS (APPI, toluene) for $C_{124}H_{130}N_4$ (M^+), calc.: 1676.0323, found: 1676.0377.

Spectroscopic data is in good agreement with the literature.[6]

2.3 Synthesis of tri porphyrin substituted HBCs 7, 8

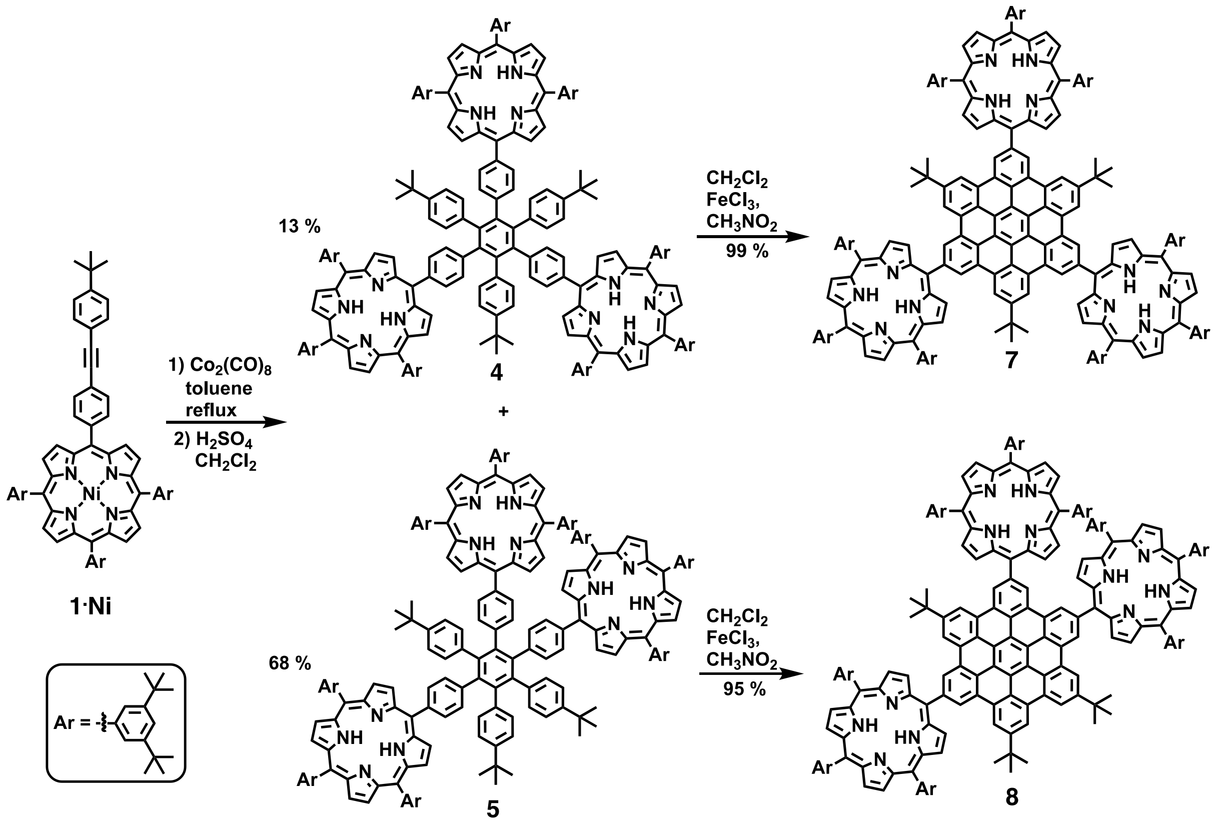

Scheme S2. Synthesis of tri porphyrin substituted HBCs **7**, **8**.

Cyclotrimerization

Nickel-tolane-porphyrin **1·Ni** (156 mg, 0.134 mmol, 1 equiv) and $Co_2(CO)_8$ (4.6 mg, 0.013 mmol, 0.1 equiv) were dissolved in toluene (2.5 mL), sealed with a septum and degassed (N_2 bubbling through the solution for 30 min). After degassing, approx. 1 mL of solvent remained. The reaction was heated (light protected) to 140 °C for 17 h. The solvent was removed and the crude pre-purified by silica plug filtration (hexanes/CH_2Cl_2 - 4:1). After removal of the solvent, the mixture of the two isomers was dissolved in CH_2Cl_2 (15 mL) and cooled with an ice bath. Conc. H_2SO_4 (1.5 mL) was added and the reaction was stirred for 30 min at 0 °C. The acid was slowly quenched with NEt_3 (10 mL) and the crude was poured on a plug of silica (hexanes/CH_2Cl_2 - 1:1). After removal of the solvent the two isomers could be separated by column chromatography (SiO_2, hexanes/CH_2Cl_2 - 2:1, ∅ 4.5 cm · 33 cm). The major isomer, A_2B_2AB **5**, was obtained in 68.0 % yield (101 mg, 30.4 μmol) and the minor isomer, $(AB)_3$ **4**, in 12.8 % yield (19 mg, 5.7 μmol).

$(AB)_3$ Free base porphyrin-HPB 4

^{1}H NMR (400 MHz, $CDCl_3$, rt): δ [ppm] = 8.98 (d, *J* = 4.7 Hz, 6H), 8.94 (d, *J* = 4.3 Hz, 6H), 8.85 (d, *J* = 4.5 Hz, 6H), 8.62 (d, *J* = 4.3 Hz, 6H), 8.15 (d, *J* = 1.9 Hz, 12H), 8.11 (d, *J* = 1.9 Hz, 6H),

7.92 (d, J = 8.0 Hz, 6H), 7.88 (d, J = 1.8 Hz, 6H), 7.82 (d, J = 1.8 Hz, 3H), 7.60 – 7.46 (m, 18H), 1.61 (s, 135H), 1.55 (s, 54H), -2.69 (s, 6H).

^{13}C NMR (101 MHz, $CDCl_3$, rt): δ [ppm] = 149.0, 148.84, 148.75, 141.5, 141.3, 141.1, 140.92, 140.86, 139.2, 138.4, 133.1, 132.1, 130.1, 130.0, 129.8, 124.1, 121.3, 121.0, 120.0, 35.04, 35.00, 34.7, 31.8, 31.7.

UV/Vis (THF): λ [nm] (ε [$M^{-1}cm^{-1}$]) = 421 (1350000), 516 (57200), 550 (33400), 593 (18500), 648 (16800).

Fluorescence (THF): $\lambda_{exc.}$ [nm] = 421, **$\lambda_{emission}$ [nm] (rel. int.)** = 653 (1.00), 718 (0.21).

MS (MALDI, DCTB): m/z (rel. int.) = 3322.84 (M^+, 100 %).

HRMS (ESI, CH_3CN) for $C_{240}H_{270}N_{12}$ (M^{2+}), calc.: 1662.0854, found: 1662.0841.

Spectroscopic data is in good agreement with the literature (synthesized via mixed cyclotrimerization reaction).[1]

A_2B_2AB Free base porphyrin-HPB 5

^{1}H NMR (400 MHz, $CDCl_3$, rt): δ [ppm] = δ 8.98 – 8.57 (m, 24H), 8.20 – 8.02 (m), 7.96 (s), 7.89 – 7.76 (m), 7.67 – 7.58 (m), 7.54 – 7.43 (m), 7.41 – 7.27 (m), 7.06 – 6.93 (m), 1.66 – 1.46 (m), 1.42 (s), 0.62 (s), -2.73 (s, 2H), -2.77 (s, 4H).

^{13}C NMR (101 MHz, $CDCl_3$, rt): δ [ppm] = 157.3, 148.74, 148.66, 148.6, 148.51, 148.45, 147.9, 141.6, 141.5, 141.2, 140.9, 140.6, 139.7, 139.1, 138.4, 138.1, 133.0, 132.8, 132.1, 131.7, 131.3, 131.1, 130.1, 129.9, 129.73, 129.73, 129.65, 129.0, 127.2, 124.0, 123.8, 123.2, 121.3, 121.11, 121.07, 120.9, 120.0, 119.7, 119.0, 35.11, 35.08, 35.05, 34.6, 34.5, 34.4, 34.1, 31.82, 31.77, 31.7, 31.6, 31.4, 30.9.

UV/Vis (THF): λ [nm] (ε [$M^{-1}cm^{-1}$]) = 420 (1310000), 515 (58200), 549 (30200), 591 (17400), 648 (15000).

Fluorescence (THF): $\lambda_{exc.}$ [nm] = 420, **$\lambda_{emission}$ [nm] (rel. int.)** = 652 (1.00), 717 (0.23).

MS (MALDI, DHB): m/z (rel. int.) = 3322.38 (M^+, 100 %).

HRMS (ESI, CH_3CN/toluene) for $C_{240}H_{270}N_{12}$ (M^{2+}), calc.: 1662.0854, found: 1662.0861.

Spectroscopic data is in good agreement with the literature (synthesized via mixed cyclotrimerization reaction).[1]

$(AB)_3$ Porphyrin-HBC 7

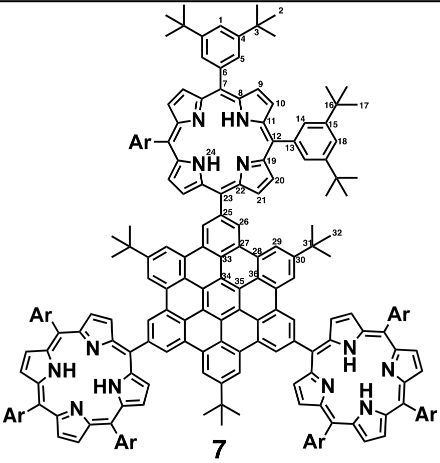

7

$C_{240}H_{258}N_{12}$
M = 3310,79

A 50 mL Schlenk tube was filled with $(AB)_3$ porphyrin-HPB **4** (30.2 mg, 9.09 µmol, 1 equiv), CH_2Cl_2 (20 mL) and cooled with an ice bath. The solution was degassed (N_2 bubbling through the solution for 15 min). The N_2 flow was increased and a solution of dry $FeCl_3$ (60 mg, 0.37 mmol, 41 equiv) in CH_3NO_2 (0.20 mL) was added. The N_2 flow was stopped 10 min after $FeCl_3$ addition and the reaction mixture was stirred under slow warming to rt for 19 h. The reaction was quenched via the addition of MeOH (10 mL) and neutralized using NEt_3 (1 mL). The solvent was removed and the crude purified by silica plug filtration (hexanes/CH_2Cl_2 - 2:1). The pure product was obtained in 99.0 % yield (29.8 mg, 23.0 µmol).

^{1}H NMR (400 MHz, $CDCl_3$, rt): δ [ppm] = 10.30 (s, 6H, H-26), 9.47 (s, 6H, H-29), 9.21 (d, *J* = 4.8 Hz, 6H, H-21), 9.07 (d, *J* = 4.9 Hz, 6H, H-20), 9.01 (bs, 12H, H-9, H-10), 8.21 (d, *J* = 1.8 Hz, 12H, H-14), 8.19 (d, *J* = 1.8 Hz, 6H, H-5), 7.85 (t, *J* = 1.7 Hz, 3H, H-1), 7.83 (t, *J* = 1.8 Hz, 6H, H-18), 1.58 (s, 54H, H-2), 1.56 (s, 108H, H-17), 1.47 (s, 27H, H-32), -2.39 (s, 6H, H-24).

^{13}C NMR (101 MHz, $CDCl_3$, rt): δ [ppm] = 150.3 (C-30), 148.9 (C-15), 148.8 (C-4), 141.5 (C-6), 141.31 (C-25 or C-34), 141.28 (C-13), 131.8 (bs, C-*β*-pyrr.), 131.6 (bs, C-*β*-pyrr.), 131.5 (bs, C-*β*-pyrr.), 130.8 (C-28), 130.0 (C-14), 129.9 (C-5), 129.4 (C-27), 128.0 (C-26), 125.7 (C-33), 125.1 (C-36), 122.3 (C-35), 121.93 (C-7), 121.86 (C-12), 121.3 (C-25 or C-34), 121.1 (C-1, C18), 120.3 (C-29), 119.8 (C-23), 35.7(C-31), 35.04 (C-3), 35.02 (C-16), 31.74 (C-32), 31.70 (C-20, C-17). NMR signals were assigned with the help of HSQC and HMBC.

UV/Vis (THF): λ [nm] (ε [$M^{-1}cm^{-1}$]) = 356 (121000), 422 (784000), 432 (808000), 516 (64400), 551 (31400), 591 (17600), 648 (14500).

Fluorescence (THF): $\lambda_{exc.}$ [nm] = 356, **$\lambda_{emission}$ [nm] (rel. int.)** = 653 (0.22), 714 (0.05).
$\lambda_{exc.}$ [nm] = 422, **$\lambda_{emission}$ [nm] (rel. int.)** = 652 (0.90), 718 (0.21).
$\lambda_{exc.}$ [nm] = 432, **$\lambda_{emission}$ [nm] (rel. int.)** = 652 (1.00), 717 (0.23).

MS (MALDI, DCTB): m/z (rel. int.) = 3310.35 (M^+, 100 %).

HRMS (ESI, CH_3CN) for $C_{240}H_{259}N_{12}$ (MH^+), calc.: 3311.0696, found: 3311.0641, $C_{240}H_{260}N_{12}$ (H_2M^{2+}), calc.: 1656.0385, found 1656.0372.

A_2B_2AB Porphyrin-HBC 8

A 50 mL Schlenk tube was filled with A_2B_2AB porphyrin-HPB **5** (80 mg, 24.1 μmol, 1 equiv), CH_2Cl_2 (25 mL) and cooled with an ice bath. The solution was degassed (N_2 bubbling through the solution for 20 min). The N_2 flow was increased and a solution of dry $FeCl_3$ (112 mg, 0.691 mmol, 29 equiv) in CH_3NO_2 (0.35 mL) was added. The N_2 bubbling was stopped 15 min after $FeCl_3$ addition and the reaction mixture was stirred under slow warming to rt for 17 h. The reaction was quenched via the addition of MeOH (10 mL) and neutralized using NEt_3 (1 mL). The solvent was removed and the crude purified by silica plug filtration (hexanes/CH_2Cl_2 - 1:1). The pure product was obtained in 95.4% yield (76 mg, 23.0 μmol).

^{1}H NMR (400 MHz, $CDCl_3/NEt_3$, rt): δ [ppm] = 10.32 (s, 1H), 10.29 (s, 1H), 10.27 (s, 1H), 10.25 (s, 1H), 10.23 (s, 2H), 9.51 (s, 2H), 9.48 (s, 1H), 9.46 (s, 1H), 9.44 (s, 1H), 9.42 (s, 1H), 9.21 (d, *J* = 4.6 Hz, 2H), 9.07 (d, *J* = 4.8 Hz, 6H), 9.02 (bs, 4H), 8.94 – 8.78 (m, 12H), 8.33 – 8.17 (m, 6H), 8.16 – 8.03 (m, 6H), 8.02 – 7.88 (m, 6H), 7.90 – 7.80 (m, 3H), 7.82 – 7.66 (m, 6H), 1.71 (s, 9H), 1.68 (s, 9H), 1.60 (s, 9H), 1.59 (s, 9H), 1.57 (s, 18H), 1.55 (s, 18H), 1.53 (s, 9H), 1.51 (s, 9H), 1.50 (s, 18H), 1.48 (s, 18H), 1.45 (s, 9H), 1.45 (s, 9H), 1.43 (s, 9H), 1.38 (s, 36H), -2.38 (s, 2H), -2.67 (s, 2H), -2.69 (s, 2H).

^{13}C NMR (101 MHz, $CDCl_3/NEt_3$, rt): δ [ppm] = δ 150.2, 150.0, 149.9, 148.9, 148.8, 148.6, 141.5, 141.4, 141.34, 141.28, 141.2, 131.3, 131.1, 130.93, 130.86, 130.7, 130.6, 130.0, 129.9, 129.8, 129.7, 129.6, 129.49, 129.45, 129.4, 129.2, 129.1, 128.1, 128.01, 127.95, 127.2, 126.6, 126.1, 126.0, 125.6, 125.1, 124.70, 124.65, 121.99, 121.95, 121.86, 121.86, 121.79, 121.72, 121.65, 121.61, 121.61, 121.55, 121.51, 121.1, 120.9, 120.3, 120.0, 119.8, 119.4, 118.9, 35.77, 35.75, 35.68, 35.0, 34.92, 34.90, 34.8, 31.9, 31.7, 31.63, 31.58.

UV/Vis (THF): λ [nm] (ε [$M^{-1}cm^{-1}$]) = 357 (127000), 420 (722000), 432 (711000), 516 (64900), 551 (31500), 591 (18700), 648 (15400).

Fluorescence (THF): $\lambda_{exc.}$ [nm] = 357, **$\lambda_{emission}$ [nm] (rel. int.)** = 652 (0.19), 717 (0.05).
$\lambda_{exc.}$ [nm] = 420, **$\lambda_{emission}$ [nm] (rel. int.)** = 652 (0.94), 717 (0.22).
$\lambda_{exc.}$ [nm] = 432, **$\lambda_{emission}$ [nm] (rel. int.)** = 652 (1.00), 717 (0.23).

MS (MALDI, DHB): m/z (rel. int.) = 3310.35 (M^+, 100 %).

HRMS (MALDI, DHB) for $C_{240}H_{258}N_{12}$ (M^+), calc.: 3310.0625, found: 3310.0814.

2.4 Attempt to synthesize hexa-porphyrin-HBC 11

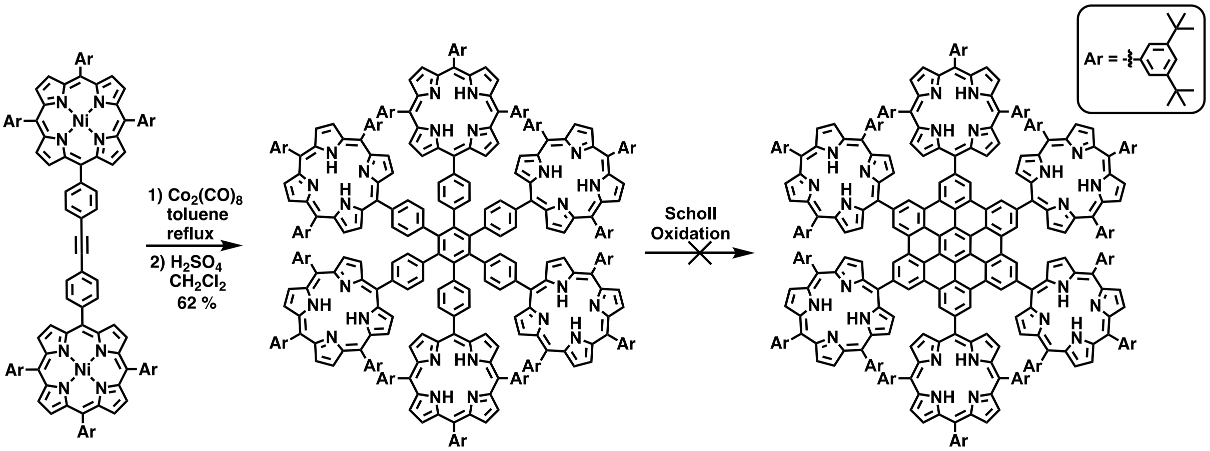

9·Ni2 10 11

Scheme S3. Attempt to synthesize hexa-porphyrin-HBC **11**.

Hexa-Porphyrin-HPB 10

Modified procedure from Osuka *et al.*[7], Gust *et al.*[8] and Jux *et al.*[1]

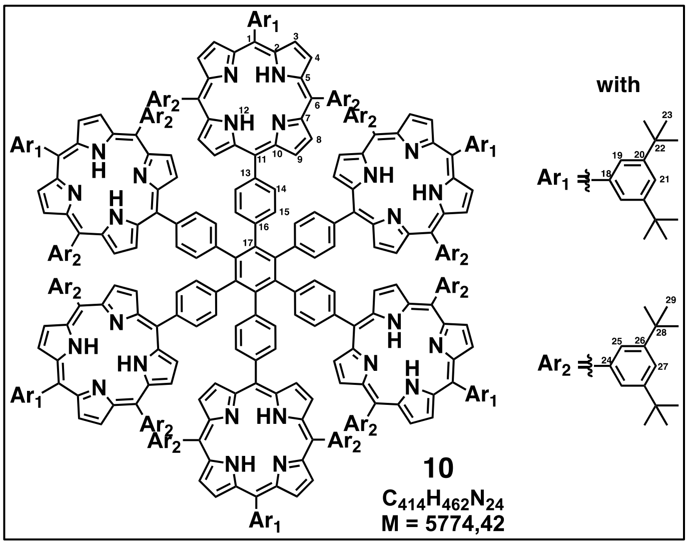

A 5 mL vial was charged with nickel-porphyrin dimer **9·Ni₂** (65 mg, 31.9 μmol, 1 equiv), $Co_2(CO)_8$ (8.2 mg, 23.9 μmol, 0.75 equiv), toluene (2.5 mL) and sealed with a septum. The solution was degassed (N_2 bubbling through the solution for 20 min) and heated with an oil bath to 140 °C for 19 h. The crude was pre-purified by silica plug filtration (hexanes/CH_2Cl_2 - 2:1). After removal of the solvent, the residue was dissolved in CH_2Cl_2 (10 mL) and cooled with an ice bath. Conc. H_2SO_4 (1 mL) was added and the reaction was stirred for 60 min at 0 °C. The acid was slowly quenched via the addition of NEt_3 (10 mL) and the inorganic salts were removed by silica plug filtration (hexanes/CH_2Cl_2 - 2:1). The product was purified by column chromatography (SiO_2, hexanes/CH_2Cl_2 - 2:1, ∅ 3.5 cm · 14 cm) and obtained as a purple solid in 61.9 % yield (38 mg, 6.58 μmol).

^{1}H NMR (500 MHz, $CDCl_3$, rt): δ [ppm] = 8.76 (d, *J* = 4.7 Hz, 12H, H-*β*) 8.70 (d, *J* = 4.7 Hz, 12H, H-*β*), 8.60 (d, *J* = 4.8 Hz, 12H, H-*β*), 8.45 (d, *J* = 7.5 Hz, 12H, H-14/15), 8.23 (d, *J* = 7.6 Hz, 12H, H-14/15), 8.06 (d, *J* = 1.6 Hz, 12H, Ar_1, H-19), 7.97 (d, *J* = 4.7 Hz, 12H, H-*β*), 7.78 (t, *J* = 1.7 Hz,

6H, Ar_1, H-21), 7.48 (very broad signal, 24H, Ar_2, H-25), 7.04 (s, 12H, Ar_2, H-27), 1.53 (s, 108H, Ar_1, H-23), 1.42 – -0.30 (very broad signal, 216H, Ar_2, H-29), -2.86 (s, 12H, N-H, H-12).

^{13}C NMR (126 MHz, $CDCl_3$, rt): δ [ppm] = 148.6 (Ar_1, C-20), 147.9 (Ar_2, C-26), 141.74, 141.69, 140.9, 140.8, 133.5 (C-14/15), 130.8 (C-14/15), 129.6 (Ar_1, C-19), 128.9 (bs, Ar_2, C-25), 128.7, 121.0 (Ar_1, Ar_2 C-21, C-27), 120.8, 120.3, 119.4, 35.0 (Ar_1, C-22), 34.1 (bs, Ar_2, C-28), 31.8 (Ar_1, C-23), 30.9 (bs, Ar_2, C-29). NMR signals were assigned with the help of COSY, HSQC and HMBC.

UV/Vis (THF): λ [nm] (ε [$M^{-1}cm^{-1}$]) = 420 (2700000), 515 (114000), 549 (52000), 591 (32000), 647 (24100).

MS (MALDI, DHB): m/z (rel. int.) = 5773.06 (M^+, 100 %).

HRMS (ESI, CH_3CN/toluene/formic acid) for $C_{414}H_{462}N_{24}$ (MH_2^{2+}), calc.: 2887.8583, found: 2887.8516.

Spectroscopic data is in good agreement with the literature (synthesized via mixed cyclotrimerization reaction).[1]

Scholl oxidation with Iron(III)chloride with 10

Free-base-hexa-porphyrin-HPB **10** (18.0 mg, 3.12 μmol, 1 equiv) was dissolved in CH_2Cl_2 (10 mL), cooled with an ice bath and degassed (bubbling N_2 through the solution for 15 min). A solution of dry $FeCl_3$ (60 mg, 370 μmol, 118 equiv) in CH_3NO_2 (0.20 mL) was added and the N_2 flow through the solution was maintained for further 10 min. The reaction was stirred for 20 h under slow warming to rt before it was quenched with MeOH (10 mL) and neutralized with NEt_3 (1 mL). The reaction mixture was purified by silica plug filtration (hexanes/CH_2Cl_2 - 2:1). TLC showed no difference to starting material and ^{1}H NMR confirmed that no reaction has occurred.

Scholl oxidation with DDQ and triflic acid with 10

Modified procedure from Jones *et al.*[9]

Free-base-hexa-porphyrin-HPB **10** (19.0 mg, 3.29 μmol, 1 equiv) and DDQ (6.9 mg, 30.4 μmol, 9.2 equiv) were dissolved in CH_2Cl_2 (3 mL), cooled with an ice bath and degassed (N_2 bubbling through the solution for 15 min). Triflic acid (7 μl, 11.9 mg, 79.0 μmol, 24 equiv) dissolved in CH_2Cl_2 (133 μl) was added and the reaction was stirred under slow warming to rt for 17 h. After 17 h TLC showed no difference to starting material. Therefore, further triflic

acid (7 μl, 11.9 mg, 79.0 μmol, 24 equiv) dissolved in CH_2Cl_2 (133 μl) was added and the reaction was stirred for 4.5 h at rt. The acid was quenched with NEt_3 (1 mL) and the reaction mixture purified by silica plug filtration (hexanes/CH_2Cl_2 – 2:1). The reaction has not worked and 18.1 mg of starting material were recovered.

Hexa-nickel-porphyrin-HPB 10·Ni$_6$

A 50 mL flask charged with free-base-hexa-porphyrin-HPB **10** (18.1 mg, 3.13 μmol, 1 equiv), $Ni(acac)_2$ (24 mg, 94 μmol, 30 equiv) and toluene (5 mL) was heated to reflux (heat on: 140 °C) for 90 min. The solvent was removed and the product purified by silica plug filtration (hexanes/CH_2Cl_2 – 2:1). **10·Ni$_6$** was obtained as a red solid in 94.2 % yield (18.0 mg, 2.95 μmol).

^{1}H NMR (400 MHz, CDCl$_3$, rt): δ [ppm] = 8.67 (d, *J* = 4.9 Hz, 12H, H-*β*), 8.57 (d, *J* = 5.0 Hz, 12H, H-*β*), 8.54 (d, *J* = 5.0 Hz, 12H, H-*β*), 8.10 (d, *J* = 7.9 Hz, 12H), 8.06 (d, *J* = 4.9 Hz, 12H, H-*β*), 7.95 (d, *J* = 7.8 Hz, 12H), 7.83 (d, *J* = 1.8 Hz, 12H), 7.69 (t, *J* = 2.0 Hz, 6H), 7.40 (bs, 24H), 7.15 (t, *J* = 2.0 Hz, 12H), 1.47 (s, 108H), 0.99 – 0.72 (m, 216H).

UV/Vis (CH$_2$Cl$_2$): λ [nm] = 417, 528.

MS (MALDI, DCTB): m/z (rel. int.) = 6114.08 (M^+, 100 %).

HRMS (ESI, CH$_3$CN/toluene/formic acid) for $C_{414}H_{450}N_{24}Ni_6$ (M^{2+}), calc.: 3056.6060, found: 3056.6084.

Scholl oxidation with Iron(III)chloride with 10·Ni$_6$

Hexa-nickel-porphyrin-HPB **10·Ni$_6$** (18.0 mg, 2.95 μmol, 1 equiv) was dissolved in CH_2Cl_2 (20 mL), cooled with an ice bath and degassed (N_2 bubbling through the solution for 15 min). The N_2 flow was increased and a solution of dry $FeCl_3$ (60 mg, 370 μmol, 125 equiv) in CH_3NO_2 (0.20 mL) was added. The N_2 flow was maintained for further 10 min and the reaction stirred under slow warming to rt for 66 h. The reaction was quenched with MeOH (10 mL) and neutralized with NEt_3 (1 mL). The solvent was removed and the crude was purified by silica plug filtration (hexanes/CH_2Cl_2 – 1:1). MALDI-ToF, ^{1}H NMR and UV/Vis showed clearly that a reaction has happened. However, the desired hexa-porphyrin-HBC was definitely not formed.

^{1}H NMR (400 MHz, CDCl$_3$, rt): No sharp signals. Only broad multiplets.

UV/Vis (CH$_2$Cl$_2$): Broad absorption from λ = 350 nm to λ = 600 nm.

MS (MALDI, DCTB): m/z (rel. int.) = 6097.17 (M^+, 100 %).

2.5 Synthesis of hexa-porphyrin-HBC 11 via Suzuki reaction

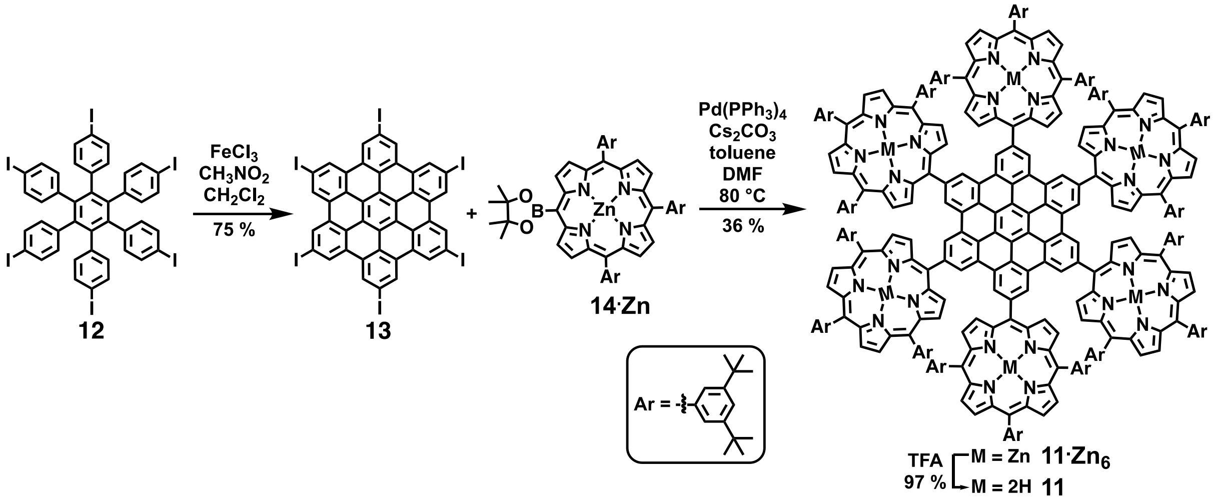

Scheme S4. Synthesis of hexa-porphyrin-HBC **11**.

Hexa(4-iodophenyl)benzene 12

Modified procedure from Mössinger *et al.* and Kobayashi *et al.*[10-11]

Hexaphenylbenzene (380 mg, 0.71 mmol, 1 equiv) was dissolved in CH_2Cl_2 (30 mL) and degassed. [Bis(trifluoroacetoxy)iodo]benzene (1.03 g, 2.40 mmol, 3.4 equiv) and I_2 (0.60 g, 2.63 mmol, 3.3 equiv) was added and the reaction was stirred light protected at rt for 68 h. The product was precipitated via the addition of hexanes (50 mL), filtered off and washed with hexanes. The solid was dissolved in $CHCl_3$, washed with aqueous 10 % $Na_2S_2O_3$ (1 x 200 mL), sat. NaCl solution (1 x 100 mL) and dried over Na_2SO_4. The pure product could be obtained after recrystallization from $CHCl_3$/hexanes (dissolve in 40 mL hot $CHCl_3$ and precipitate via the addition of 50 mL hexanes) as a white solid in 55.0 % yield (504 mg, 0.39 mmol).

^{1}H NMR (400 MHz, CD_2Cl_2, rt): δ [ppm] = 7.27 (d, *J* = 8.4 Hz, 12H), 6.54 (d, *J* = 8.3 Hz, 12H).

^{13}C NMR (101 MHz, CD_2Cl_2, rt): δ [ppm] = 139.9, 139.7, 136.6, 133.4, 92.1.

HRMS (APPI, toluene/CH_2Cl_2) for $C_{42}H_{24}I_6$ (M^+), calc.: 1289.6141, found: 1289.6150.

Hexa(4-iodo)-*peri*-hexabenzocoronene 13

Modified procedure from Müllen *et al.*[12]

Hexa(4-iodophenyl)benzene **12** (0.40 g, 0.31 mmol, 1 equiv) was dissolved in CH_2Cl_2 (150 mL), cooled with an ice bath and degassed (bubbling N_2 through the solution for 20 min). A solution of dry $FeCl_3$ (1.61 g, 9.93 mmol, 32 equiv) in CH_3NO_2 (4.0 mL) was added and the N_2 flow was maintained for further 20 min. The reaction was stirred under slow warming to rt for 17 h and quenched via the addition of MeOH (50 mL). The insoluble product was filtered off and washed with lots of MeOH and CH_2Cl_2 until the filtrate was

colourless. The product could be obtained as a brown insoluble solid in 75.0 % yield (297 mg, 0.23 mmol).

Hexa-Zinc-Porphyrin-HBC 11·Zn$_6$

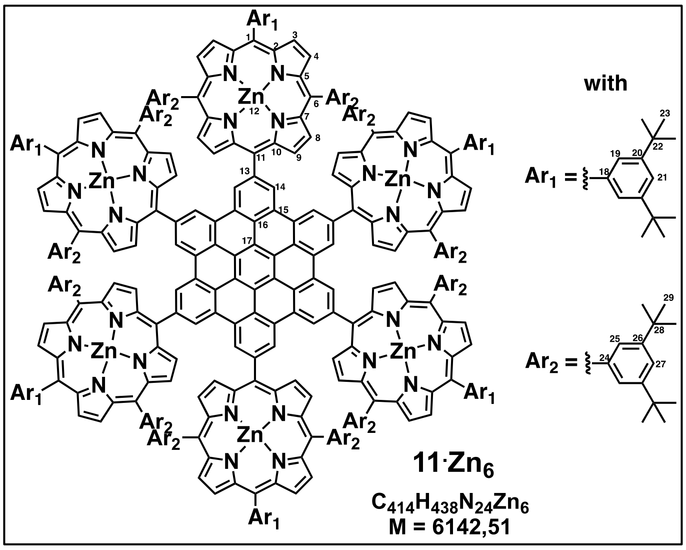

A Schlenk flask was charged with hexa(4-iodo)-HBC **12** (10.0 mg, 7.82 μmol, 1 equiv), boronic-ester-porphyrin **14·Zn** (50.0 mg, 47.0 μmol, 6 equiv), Cs_2CO_3 (23 mg, 70.4 μmol, 9 equiv), toluene (2 mL) and DMF (1 mL). The reaction mixture was degassed, $Pd(PPh_3)_4$ (5.4 mg, 4.7 μmol, 0.6 equiv) was added and the reaction was heated with an oil bath to 80 °C for 16 h. The solvent was removed and the crude purified by column chromatography (SiO_2, hexanes/CH_2Cl_2 – 2:1, ∅ 5 cm · 22 cm). The first band was identified as the product and was further purified by recrystallization from CH_2Cl_2/MeOH. The was obtained as a red-brownish solid in 35.9 % yield (17.2 mg, 2.81 μmol).

^{1}H NMR (500 MHz, $CDCl_3$, rt): δ [ppm] = 10.33 (s, 12H, H-14), 9.15 (d, *J* = 4.7 Hz, 12H, H-*β*), 8.83 (d, *J* = 4.5 Hz, 12H, H-*β*), 8.74 (d, *J* = 4.7 Hz, 12H, H-*β*), 8.73 (d, *J* = 4.7 Hz, 12H, H-*β*), 7.84 (d, *J* = 1.9 Hz, 12H, H-19), 7.80 (d, *J* = 1.8 Hz, 24H, H-25), 7.66 (t, *J* = 1.8 Hz, 12H, H-27), 7.63 (t, *J* = 1.9 Hz, 6H, H-21), 1.36 (s, 324H, H-23, H-29).

^{13}C NMR (126 MHz, $CDCl_3$, rt): δ [ppm] = 150.3 (C-*α*), 150.1 (C-*α*), 148.3 (C-20), 148.2 (C-26), 142.7, 141.83, 141.78, 132.5 (C-*β*), 131.94 (C-*β*), 131.85 (C-*β*), 129.4 (C-19, C-25), 128.3 (C-14), 126.6, 123.0, 122.4, 120.5 (C-21, C-27), 119.9, 34.8 (C-22, C-28), 31.6 (C-29), 31.5 (C-23). NMR signals were assigned with the help of COSY, HSQC and HMBC. With C-*α* = C-2, C-5, C-7, C-10 and C-*β* = C-3, C-4, C-8, C-9.

UV/Vis (THF): λ [nm] (ε [M^{-1}cm^{-1}]) = 420 (844000), 438 (1085000), 557 (97500), 597 (36800).

Fluorescence (THF): λ$_{exc.}$ [nm] = 355, λ$_{emission}$ [nm] (rel. int.) = 602 (0.11), 654 (0.09).

λ$_{exc.}$ [nm] = 420, λ$_{emission}$ [nm] (rel. int.) = 602 (0.80), 654 (0.68).

λ$_{exc.}$ [nm] = 437, λ$_{emission}$ [nm] (rel. int.) = 602 (1.00), 654 (0.85).

MS (MALDI, DHB): m/z (rel. int.) = 6145.57 (M$^+$, 100 %).

HRMS (ESI, CH_3CN/toluene) for $C_{414}H_{438}N_{24}Zn_6$ (M^{2+}), calc.: 3071.0395, found: 3071.0337.

Hexa free base porphyrin-HBC 11

Hexa-zinc-porphyrin-HBC **11·Zn$_6$** (22.8 mg, 3.71 μmol) was dissolved in $CHCl_3$ (10 mL) and trifluoroacetic acid (100 μl, 149 mg, 1.31 mmol) was added. The reaction was stirred for 60 min at rt. The green solution was neutralized with NEt_3 (1 mL) and purified by silica plug filtration (hexanes/CH_2Cl_2 – 2:1). After recrystallization from CH_2Cl_2/MeOH the pure product was obtained in 96.8 % yield (20.7 mg, 3.59 μmol).

^{1}H NMR (500 MHz, CDCl$_3$, rt): δ [ppm] = 10.33 (s, 12H, H-14), 9.06 (d, *J* = 4.6 Hz, 12H, H-*β*), 8.74 (d, *J* = 4.7 Hz, 12H, H-*β*), 8.70 – 8.56 (m, 24H, H-*β*), 7.85 (d, *J* = 1.9 Hz, 12H, H-19), 7.80 (d, *J* = 1.8 Hz, 24H, H-25), 7.69 – 7.61 (m, 18H, H-21, H-27), 1.36 (s, 108H, H-23), 1.34 (s, 216H, H-29), -2.94 (s, 12H, H-12).

^{13}C NMR (126 MHz, CDCl$_3$, rt): δ [ppm] = 148.5 (C-20, C-26), 142.2, 141.3, 141.2, 131.2 (bs, C-β), 129.5 (C-19, C-25), 128.6 (C-14), 126.7, 123.0, 121.4, 120.8 (C-21, C-27), 118.8, 34.8 (C-22, C-26), 31.59 (C-29), 31.55 (C-23). NMR signals were assigned with the help of HSQC and HMBC.

UV/Vis (THF): λ [nm] (ε [$M^{-1}cm^{-1}$]) = 416 (1040000), 432 (1220000), 516 (118000), 551 (59500), 591 (32700), 648 (26600).

Fluorescence (THF): **$\lambda_{exc.}$ [nm] = 356, $\lambda_{emission}$ [nm] (rel. int.)** = 650 (0.23), 716 (0.05).
$\lambda_{exc.}$ [nm] = 416, $\lambda_{emission}$ [nm] (rel. int.) = 650 (0.83), 716 (0.19).
$\lambda_{exc.}$ [nm] = 432, $\lambda_{emission}$ [nm] (rel. int.) = 650 (1.00), 716 (0.23).

MS (MALDI, DCTB): m/z (rel. int.) = 5766.72 (M^+, 100 %).

HRMS (ESI, CH_3CN/formic acid) for $C_{414}H_{450}N_{24}$ (MH^{2+}), calc.: 2881.8113, found: 2881.8151.

2.6 Synthesis of boronic-ester-porphyrin 14·Zn

*Boronic-ester-porphyrin **14·Zn** was prepared according to our recently reported synthetic procedure.*[3]

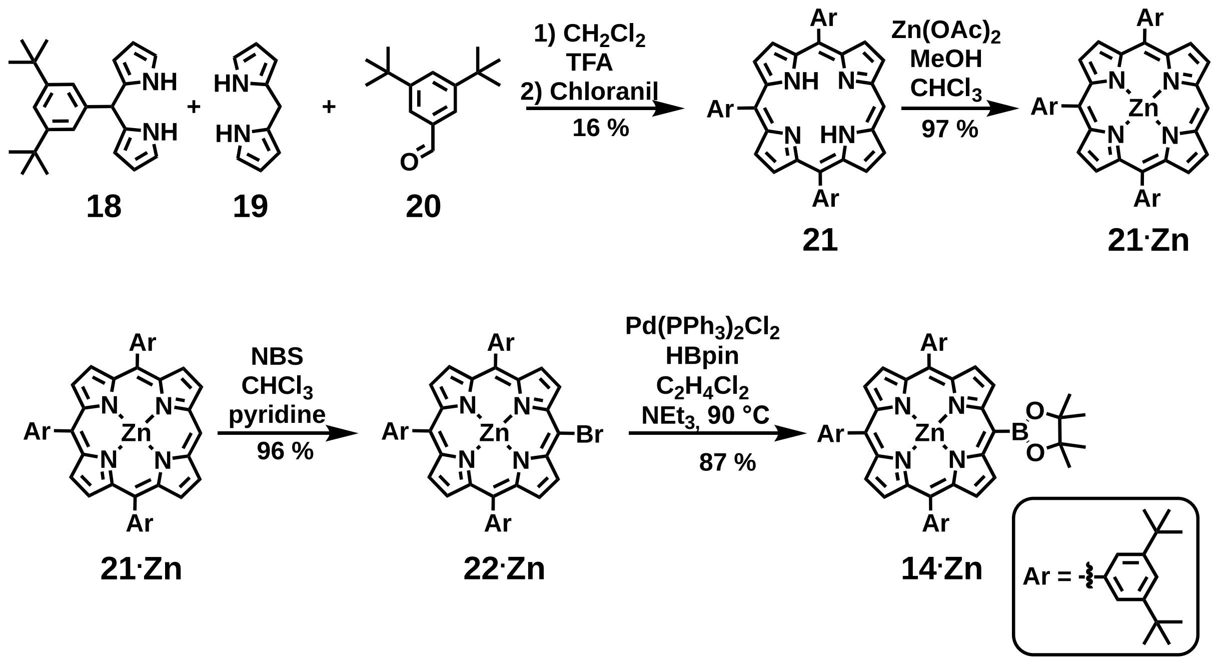

Scheme S5. Synthesis of boronic-ester-porphyrin **14·Zn**.[3]

Meso-free-porphyrin 21[3]

Dipyrromethane[4] (0.88 g, 6.00 mmol, 1 equiv), 3,5 di-*tert*-butylphenyl-dipyrromethane[5] (2.00 g, 6.0 mmol, 1 equiv) and 3,5 di-*tert*-butylbenzaldehyde (2.62 g, 12.00 mmol, 2 equiv) were dissolved in CH_2Cl_2 (2 L) and degassed for 15 min (N_2 bubbling through the solution). Trifluoroacetic acid (0.60 mL, 7.50 mmol, 1.25 equiv) was added and the reaction was stirred for 2 h at rt under the exclusion of light. *para*-Chloranil (4.43 g, 18 mmol, 3 equiv) was added and the mixture was stirred for further 1 h under air. The acid was quenched via the addition of NEt_3 (10 mL), the solvent was removed and the crude purified by silica plug filtration (hexanes/CH_2Cl_2 – 1:1, ∅ 13 cm). Three different porphyrins were separated by column chromatography (SiO_2, hexanes/CH_2Cl_2 – 3:1, ∅ 10 cm · 33 cm). The second fraction was identified as the desired product and obtained in 16.5 % yield (864 mg, 0.99 mmol). The byproducts 5,10,15,20-tetrakis(3,5-di-*tert*-butylphenyl)porphyrin and 5,15-bis(3,5-di-*tert*-butylphenyl)-porphyrin were obtained in 5.4 % (346 mg, 0.33 mmol) and 9.6 % (397 mg, 0.58 mmol) yield, respectively.

Meso-free-zinc-porphyrin 21·Zn[3]

Free-base porphyrin **21** (243 mg, 277 μmol) was dissolved in $CHCl_3$ (25 mL), a saturated solution of $Zn(OAc)_2 \cdot 2\,H_2O$ in MeOH (10 mL) was added and the mixture was stirred under

light exclusion for 18 h at rt. The solvent was removed under reduced pressure and the excess zinc salt removed by silica plug filtration (CH_2Cl_2). The product was obtained in 97.5 % yield (253 mg, 270 μmol).

Bromo-porphyrin 22·Zn[3]

N-bromosuccinimide (43 mg, 240 μmol, 1 equiv), dissolved in $CHCl_3$ (5 mL), was added slowly at rt to a solution of porphyrin **21·Zn** (225 mg, 240 μmol, 1 equiv) in $CHCl_3$ (15 mL) and pyridine (340 μl). The mixture was stirred for 15 min at rt before the reaction was quenched with acetone (5 mL). The solvent was removed and the residue purified by silica plug filtration (hexanes/CH_2Cl_2 - 2:1). The product was obtained in 95.8 % yield (234 mg, 230 μmol).

Boronic-ester-porphyrin 14·Zn[3]

Bromo-porphyrin **22·Zn** (204 mg, 200 μmol, 1 equiv), $Pd(PPh_3)_2Cl_2$ (7 mg, 10 μmol, 0.05 equiv) and NEt_3 (0.50 mL) were dissolved in dry 1,2 dichloroethane (25 mL) in a pressure vial. The reaction mixture was degassed. Pinacolborane (242 μl, 213 mg, 1.67 mmol, 8.35 equiv) was added and the pressure vial was sealed. The reaction mixture was stirred for 2.5 h at 90 °C under exclusion of light. The solvent was removed and the crude product purified by column chromatography (SiO_2, hexanes/CH_2Cl_2 - 2:1). The product could be obtained as a red solid in 87.0 % yield (185 mg, 174 μmol).

3 Crystallographic Data

Mono-Porphyrin-HPB 3

Single crystals of mono-porphyrin-HPB **3** were crystalized from benzene/MeOH. A suitable crystal was selected and mounted on a loop on a SuperNova, Dual, Cu at zero, Atlas diffractometer by Agilent Technologies GmbH. The crystal was kept at 153.00(10) K during data collection. Using Olex2[13] , the structure was solved with the ShelXT[14] structure solution program using Intrinsic Phasing and refined with the ShelXL[15] refinement package using Least Squares minimization

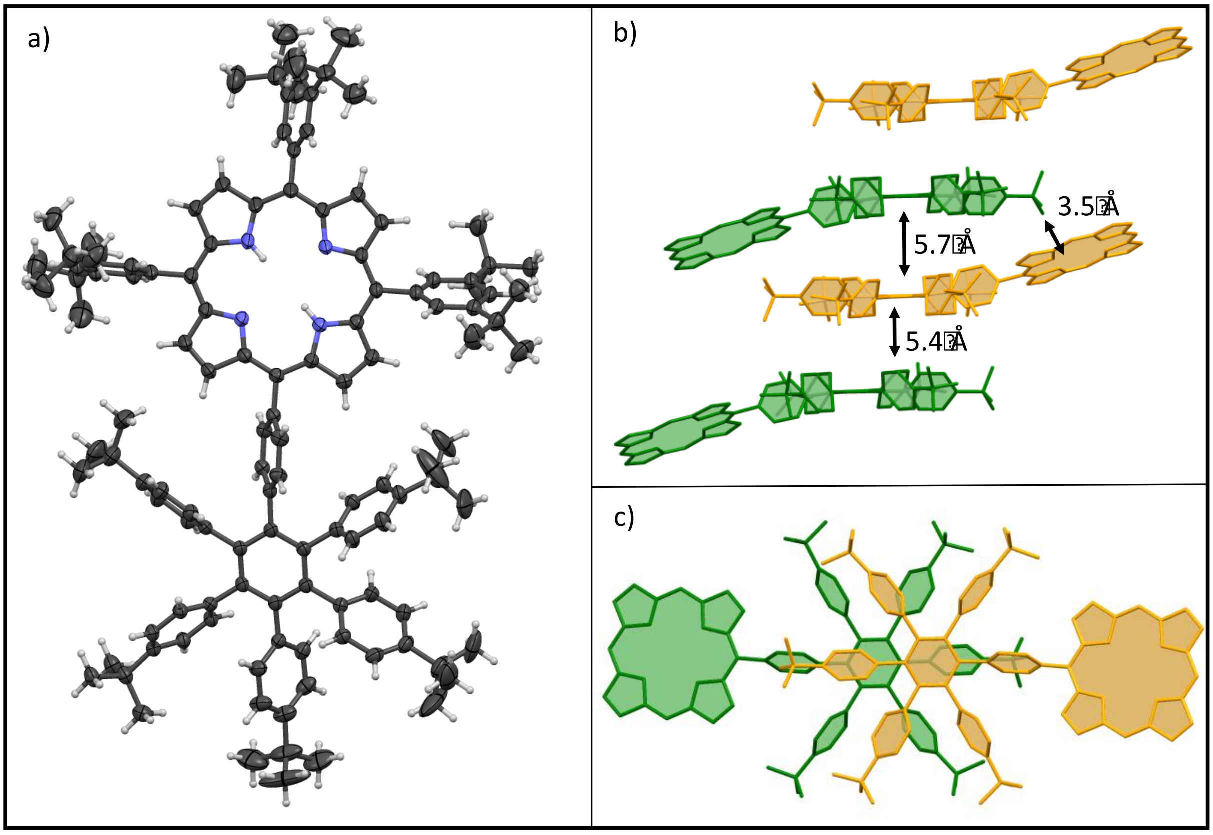

Figure S1. Crystal structure of mono-porphyrin-HPB **3**; ORTEP representation with thermal ellipsoids drawn at a 50 % probability; b) side view of four molecules of **3**; c) top view of two molecules of **3**; b), c) 3,5-di-tert-butylphenylene groups and hydrogens are omitted for clarity. Structure was measured by Frank Hampel, e-mail: frank.hampel@fau.de.

Table S1 Crystal data and structure refinement for 17Jux_MM01.

Identification code	17Jux_MM01
Empirical formula	$C_{148}H_{166}N_4$
Formula weight	2000.84
Temperature/K	153.00(10)
Crystal system	triclinic
Space group	P-1
a/Å	11.8274(7)
b/Å	19.4515(12)
c/Å	28.0585(15)
α/°	96.691(5)
β/°	91.170(4)
γ/°	93.281(5)
Volume/Å^3	6398.3(6)
Z	2
ρ_{calc}g/cm^3	1.039
μ/mm^{-1}	0.441
F(000)	2164.0
Crystal size/mm^3	0.309 × 0.251 × 0.16
Radiation	CuKα (λ = 1.54184)
2Θ range for data collection/°	7.49 to 146.102
Index ranges	-14 ≤ h ≤ 14, -23 ≤ k ≤ 23, -24 ≤ l ≤ 34
Reflections collected	38704
Independent reflections	24307 [R_{int} = 0.0412, R_{sigma} = 0.0580]
Data/restraints/parameters	24307/430/1390
Goodness-of-fit on F^2	1.046
Final R indexes [I>=2σ (I)]	R_1 = 0.0755, wR_2 = 0.2072
Final R indexes [all data]	R_1 = 0.1153, wR_2 = 0.2464
Largest diff. peak/hole / e Å^{-3}	0.52/-0.45
CCDC No.	1904188

Mono-Porphyrin-HBC 6

Single crystals of mono-porphyrin-HBC **6** were crystalized from $CHCl_3$/MeOH. A suitable crystal was selected and mounted on a loop on a SuperNova, Dual, Cu at zero, Atlas diffractometer by Agilent Technologies GmbH. The crystal was kept at 153.00(10) K during data collection. Using Olex2[13] , the structure was solved with the ShelXT[14] structure solution program using Intrinsic Phasing and refined with the ShelXL[15] refinement package using Least Squares minimization

A single crystal X-ray structure of mono-porphyrin-HBC **6** (CCDC-No.: 1818692), grown from toluene/MeOH, was recently reported by our group.[6] This structure shows, similar to the one reported here, the formation of dimers in the solid state. Therefore, π-π interactions between the HBC planes seem to be an important driving force for crystal packing, no matter if crystals were grown from aromatic (toluene) or chlorinated ($CHCl_3$) solvents.

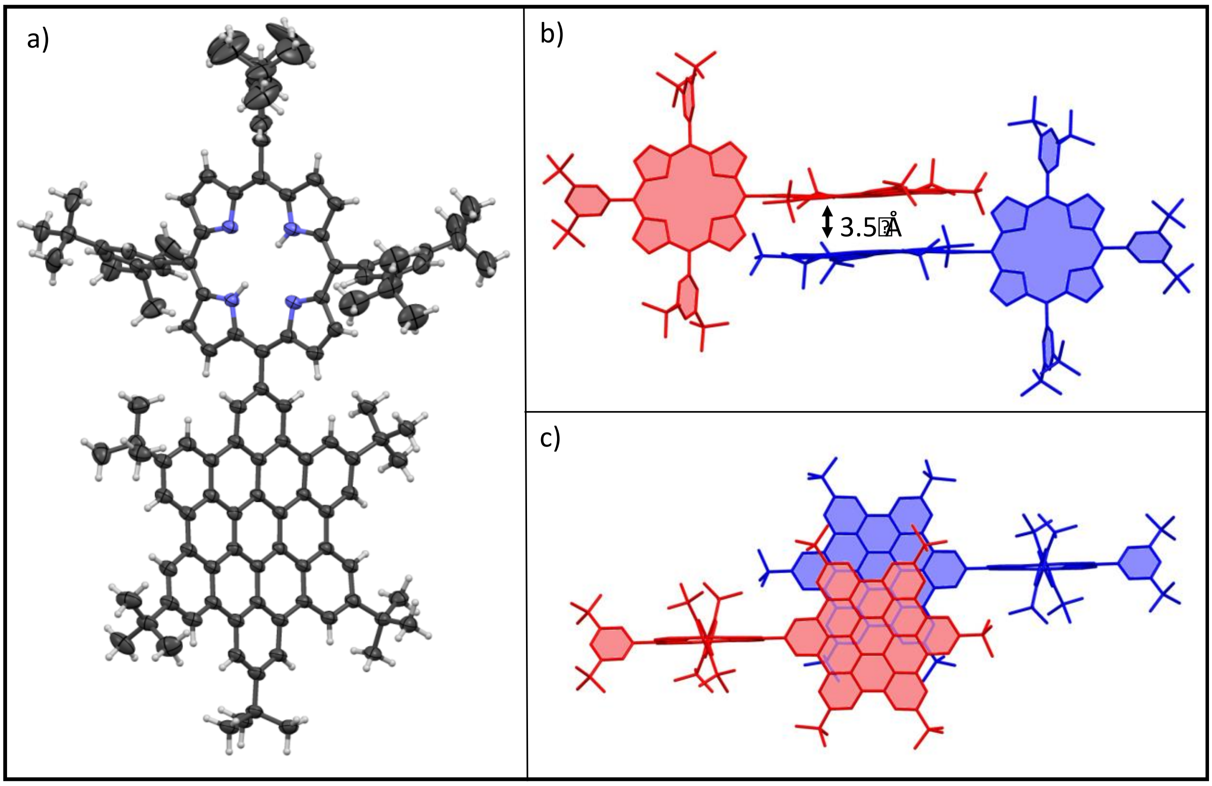

Figure S2. Crystal structure of mono-porphyrin-HBC **6**; ORTEP representation with thermal ellipsoids drawn at a 50 % probability; b) side view of two molecules of **6**; c) top view of two molecules of **6**; b), c) 3,5-di-tert-butylphenylene groups and hydrogens are omitted for clarity. Structure was measured by Frank Hampel, e-mail: frank.hampel@fau.de.

Table S2 Crystal data and structure refinement for 16Jux_MM01_2.

Identification code	16Jux_MM01_2
Empirical formula	$C_{124}H_{130}N_4$
Formula weight	1676.31
Temperature/K	153.00(10)
Crystal system	triclinic
Space group	P-1
a/Å	13.1561(5)
b/Å	20.0170(7)
c/Å	22.4974(8)
α/°	107.791(3)
β/°	100.771(3)
γ/°	102.788(3)
Volume/Å^3	5290.6(3)
Z	2
ρ_{calc}g/cm^3	1.052
μ/mm^{-1}	0.451
F(000)	1804.0
Crystal size/mm^3	0.179 × 0.101 × 0.081
Radiation	CuKα (λ = 1.54184)
2Θ range for data collection/°	7.272 to 123.216
Index ranges	-14 ≤ h ≤ 14, -22 ≤ k ≤ 19, -25 ≤ l ≤ 25
Reflections collected	23982
Independent reflections	15678 [R_{int} = 0.0541, R_{sigma} = 0.0589]
Data/restraints/parameters	15678/30/1188
Goodness-of-fit on F^2	1.071
Final R indexes [I>=2σ (I)]	R_1 = 0.0812, wR_2 = 0.2299
Final R indexes [all data]	R_1 = 0.1099, wR_2 = 0.2602
Largest diff. peak/hole / e Å^{-3}	0.41/-0.40
CCDC-No.	1904187

$(AB)_3$ Porphyrin-HPB 4

Dark brown crystals of compound **4** for X-ray diffraction analysis were grown in a mixture of toluene and MeOH. A suitable crystal of **4** was selected, embedded in inert perfluoropoly-alkylether (viscosity 1800 cSt; ABCR GmbH) and mounted using a Hampton Research CryoLoop. The selected crystal was then flash cooled to 100 K in a nitrogen gas stream and kept at this temperature during the experiment. The crystal structure of **4** was measured on a SuperNova, Dual, Cu at zero, AtlasS2 diffractometer. The selected crystal diffracted weakly and only a substandard dataset could be obtained (resolution 1.1 Å). Nevertheless, the data was sufficient to establish the connectivity of the atoms in **4** (see Figure S3a below) and the packing of the molecules within the crystal (see Figure S 3b), but precludes detailed discussion of bond lengths and angles. The data was processed with the CrysAlisPro (v38.46) software package.[16] Using Olex2,[13] the structure was solved with the ShelXT[14] structure solution program using Intrinsic Phasing and refined with the ShelXL[15] refinement package using Least Squares minimization. All non-hydrogen atoms were refined with anisotropic displacement parameters. All hydrogen atoms were placed in ideal positions and refined as riding atoms with relative isotropic displacement parameters. The disorder of 14 of the *t*-butyl groups was modelled with additional similarity restraints (SIMU, SADI) and rigid bond restraints (RIGU[17]).

The positions of the co-crystallized solvent molecules were only partially observed (2.8 toluene) and refined.

Most solvent molecules incorporated in the crystal of **4** were found to be disordered. A suitable disorder model for all other disordered solvent molecules (toluene + MeOH) could not be build. Therefore, their contribution to the structure factors was secured by back-Fourier transformation using the solvent mask routine[18-19] of the program Olex2.[13] The solvent accessible voids treated this way had a size of 2365.0 $Å^3$/unit cell (18.9%) and contained 484.8electrons/unit cell. Table S3 summarizes the crystallographic data and structure refinement details.

Due to insufficient quality, the data has not been deposited at the CCDC but can be obtained from the authors (jens.langer@fau.de).

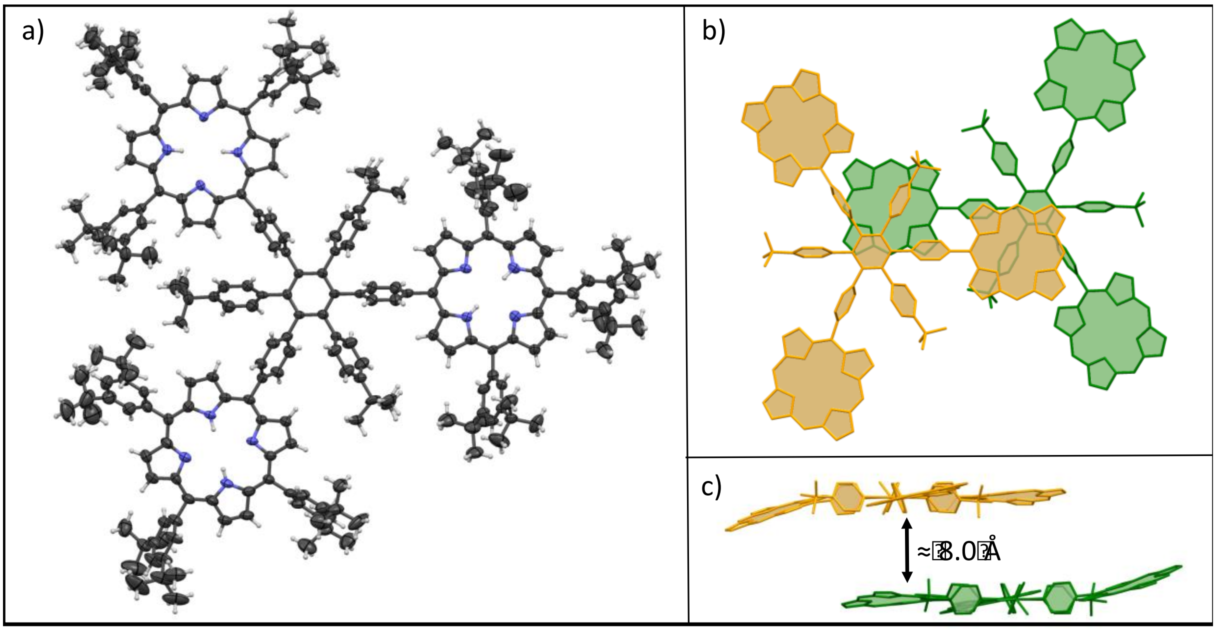

Figure S3. Structural motif of tri-porphyrin-HPB **4**; ORTEP representation with thermal ellipsoids drawn at a 50 % probability; b) top view of two molecules of **4**; c) side view of two molecules of **4**; b), c) 3,5-di-tert-butylphenylene groups and hydrogens are omitted for clarity. Structure was measured by Jens Langer, e-mail: jens.langer@fau.de.

Table S3 Crystal data and structure refinement for (AB)3 Porphyrin-HPB 4·2.8(Toluene)[+disordered solvent]

Identification code	hasj180126a_SB
Empirical formula	$C_{259.57}H_{292.37}N_{12}$ [+ disordered solvent]
Formula weight	3580.25
Temperature/K	100.0(2)
Crystal system	triclinic
Space group	P-1
a/Å	19.4226(8)
b/Å	23.1904(10)
c/Å	31.8346(10)
α/°	105.824(3)
β/°	92.113(3)
γ/°	113.192(4)
Volume/Å^3	12510.8(9)
Z	2
ρ_{calc}g/cm^3	0.950
μ/mm^{-1}	0.409
F(000)	3868.0
Crystal size/mm^3	0.664 × 0.092 × 0.053
Radiation	CuKα (λ = 1.54184)
2Θ range for data collection/°	7.06 to 89.952
Index ranges	-15 ≤ h ≤ 17, -21 ≤ k ≤ 20, -29 ≤ l ≤ 27
Reflections collected	33097

Independent reflections	19805 [R_{int} = 0.0290, R_{sigma} = 0.0525]
Data/restraints/parameters	19805/2437/3053
Goodness-of-fit on F^2	1.034
Final R indexes [I>=2σ (I)]	R_1 = 0.0802, wR_2 = 0.2185
Final R indexes [all data]	R_1 = 0.1011, wR_2 = 0.2422
Largest diff. peak/hole / e $Å^{-3}$	0.60/-0.29

Hexa-Zinc-Porphyrin-HBC 11·Zn$_6$

Crystals, suitable for XRD, were grown from benzene/MeOH solutions. Data on **11·Zn$_6$**·6MeOH·2C_6H_6 were collected at Beamline I19 of Diamond Light Source employing silicon double crystal monochromated synchrotron radiation (0.6889 Å) with ω scans at 100(2) K.[20] Data integration and reduction were undertaken with CrysalisPro.[21] Subsequent computations were carried out using the WinGX-32 graphical user interface.[22] Multi-scan empirical absorption corrections were applied to the data using SADABS.[23] The structure was solved by direct methods using SHELXT-2014[14] then refined and extended with SHELXL-2018.[15] In general, non-hydrogen atoms with occupancies greater than 0.5 were refined anisotropically. Carbon-bound hydrogen atoms were included in idealized positions and refined using a riding model. Disorder was modelled using standard crystallographic methods including constraints, restraints and rigid bodies where necessary.

The crystals employed were small and weakly diffracting and few reflections at greater than 1.2 Å resolution were observed despite the use of synchrotron radiation. Nevertheless, the quality of the data is far more than sufficient to establish the connectivity of the structure. The asymmetric unit was found to contain half of the molecule and associated solvent molecules.

Reflecting the weakly diffracting nature of the sample there is a very high level of disorder and thermal motion within the structure. One entire porphyrin group was modelled as disordered over two positions with occupancies of 0.569/0.431. The remaining 3,5-di-*tert*-butylphenylene substituents all displayed evidence of disorder and were each modelled as disordered over two locations. Due to the less than ideal resolution, extensive restraints were required to facilitate realistic modelling of these disordered groups. The GRADE program[24] was employed using the GRADE Web Server[25] to generate a full set of bond distance and angle restraints (DFIX, DANG, FLAT) for the disordered portions of the structure. Thermal parameter restraints (SIMU, RIGU) were applied to all atoms except for zinc to facilitate anisotropic refinement. Most of the disordered groups were modelled with isotropic thermal parameters. Even with these restraints the thermal parameters of the 3,5-di-*tert*-butylphenylene substituents are larger than ideal resulting in high U_{iso} min/max

ratios. The thermal motion results in part from the presence of dynamic disorder in these groups which was not modelled.

Further reflecting the poor diffraction properties there is a significant amount of void volume in the lattice containing smeared electron density from disordered solvent. Consequently the SQUEEZE[18] function of PLATON[26] was employed to remove the contribution of the electron density associated with highly disordered solvent which gave a potential solvent accessible void of 13939.9 Å^3 per unit cell (a total of approximately 2687 electrons). Since the diffuse solvent molecules could not be assigned conclusively to benzene or methanol they were not included in the formula. Consequently, the molecular weight and density given below are likely to be slightly underestimated.

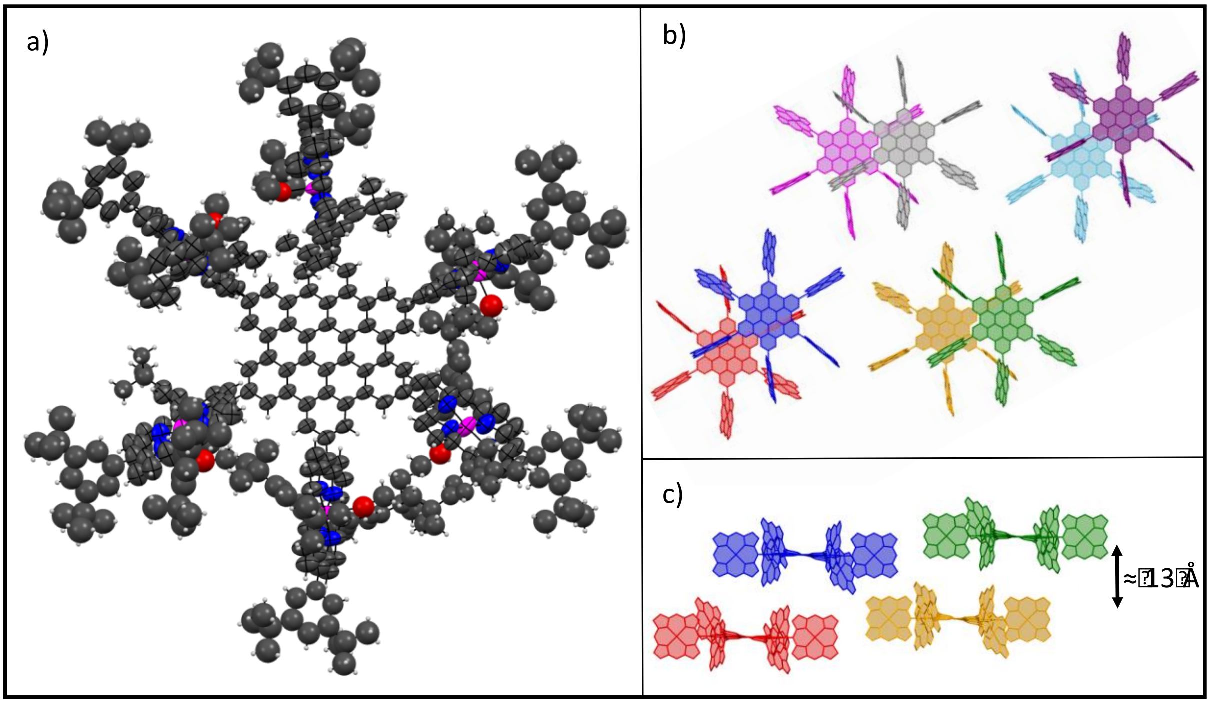

Figure S4. a) Structural motif of hexa-zinc-porphyrin-HBC **11·Zn$_6$** with MeOH coordinated to each central zinc atom; ORTEP representation with thermal ellipsoids drawn at a 40 % probability; disorder is omitted for clarity b) top view of eight molecules **11·Zn$_6$**; c) side view of four molecules **11·Zn$_6$**; b), c) 3,5-di-*tert*-butylphenylene groups and hydrogens are omitted for clarity. Structure was measured by Tanya K. Ronson, e-mail: tr352@cam.ac.uk.

Table S4 Crystal data and structure refinement for mm1_sq_without_MeOH.

Identification code	mm1_sq_without_MeOH
Empirical formula	$C_{432}H_{474}N_{24}O_6Zn_6$
Formula weight	6490.54
Temperature/K	100(2)
Crystal system	monoclinic
Space group	C2/c
a/Å	52.675(5)
b/Å	33.2176(14)
c/Å	30.9272(14)
α/°	90
β/°	102.838(7)
γ/°	90
Volume/$Å^3$	52762(6)
Z	4
$\rho_{calc}g/cm^3$	0.817
μ/mm^{-1}	0.287
F(000)	13848.0
Crystal size/mm^3	0.08 × 0.02 × 0.01
Radiation	Synchrotron (λ = 0.6889)
2Θ range for data collection/°	2.972 to 33.5
Index ranges	-44 ≤ h ≤ 44, -27 ≤ k ≤ 27, -25 ≤ l ≤ 25
Reflections collected	50471
Independent reflections	15956 [R_{int} = 0.0517, R_{sigma} = 0.0669]
Data/restraints/parameters	15956/5537/2348
Goodness-of-fit on F^2	1.050
Final R indexes [I>=2σ (I)]	R_1 = 0.1589, wR_2 = 0.3916
Final R indexes [all data]	R_1 = 0.2028, wR_2 = 0.4321
Largest diff. peak/hole / e $Å^{-3}$	0.58/-0.39
CCDC No.	1903856

Hexa-Free-Base-Porphyrin-HBC 11

Black crystals of compound **11** for X-ray diffraction analysis were grown by chance of an unknown solvent mixture. A crystal of **11** was selected, embedded in inert perfluoropoly-alkylether (viscosity 1800 cSt; ABCR GmbH) and mounted using a Hampton Research CryoLoop. The selected crystal was flash cooled to 100 K in a nitrogen gas stream and kept at this temperature during the experiment. The crystal structure of **11** was measured on a SuperNova, Dual, Cu at zero, AtlasS2 diffractometer. The selected crystal diffracted weakly and only a substandard dataset could be obtained (resolution 1.0 Å). Nevertheless, the data was sufficient to establish the connectivity of the atoms in **11** (see Figure S5a below) and the packing of the molecules within the crystal (see Figure S5b), but precludes detailed discussion of bond lengths and angles. The crystal under investigation was an inversion twin. The data was processed with the CrysAlisPro (v38.46) software package.[17] Using Olex2,[13] the structure was solved with the ShelXT[14] structure solution program using Intrinsic Phasing and refined with the ShelXL[15] refinement package using Least Squares minimization. All non-hydrogen atoms were refined with anisotropic displacement parameters. All hydrogen atoms were placed in ideal positions and refined as riding atoms with relative isotropic displacement parameters. Rigid bond restraints (RIGU[17]) and similarity restraints (SIMU) were placed on the whole structure to ensure a stable refinement. Almost all *t*-butyl groups showed signs of disorder and required additional similarity restraints (SADI, SIMU), distance restraints (DANG) and one ISOR restraint to maintain reasonable geometries and thermal ellipsoids. In two out of three cases, where a disorder model was introduced, the two alternative orientations of the *t*-Bu group were refined as rigid groups. The idealized geometry of the *t*-Bu group was taken from Guzei's 'Idealized Molecular Geometry Library'.[27] The two orientations of a disordered substituted phenyl group were modelled as rigid hexagons (AFIX 66).

The solvent molecules incorporated in the crystal of **11** were found to be heavily disordered. Therefore, their contribution to the structure factors was secured by back-Fourier transformation using the solvent mask routine[18-19] of the program Olex2.[13] The solvent accessible voids treated this way had a size of 75351.6 $Å^3$/unit cell (59.1%) and contained 10120.7 electrons/unit cell. Table S5 summarizes the crystallographic data and structure refinement details.

Due to insufficient quality, the data has not been deposited at the CCDC, but can be obtained from the authors (jens.langer@fau.de).

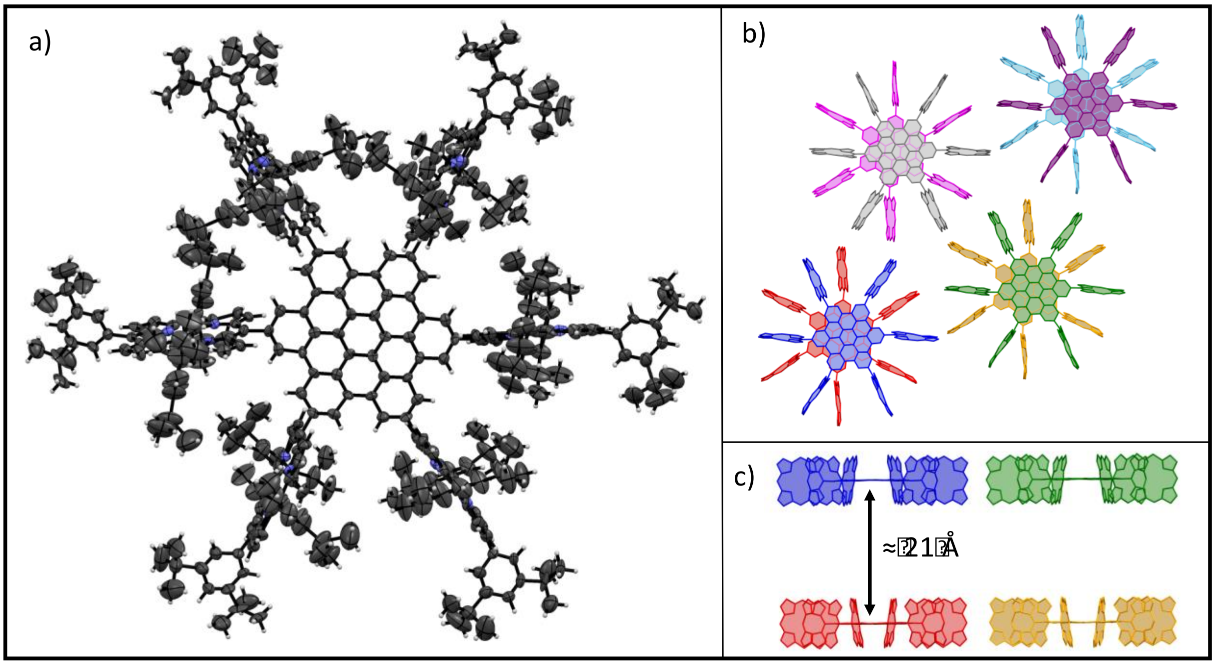

Figure S5. a) Structural motif of hexa-free-base-porphyrin-HBC **11**; ORTEP representation with thermal ellipsoids drawn at a 50 % probability; b) top view of eight molecules of **11**; c) side view of four molecules of **11**; b), c) 3,5-di-tert-butylphenylene groups and hydrogens are omitted for clarity. Structure was measured by Jens Langer, e-mail: jens.langer@fau.de.

Table S5 Crystal data and structure refinement for Hexa-Free-Base-Porphyrin-HBC 11 [+disordered solvent].

Identification code	hasj180413b_SB_1
Empirical formula	$C_{414}H_{450}N_{24}$ [+disordered solvent]
Formula weight	5761.92
Temperature/K	100.0(2)
Crystal system	hexagonal
Space group	P622
a/Å	60.1634(5)
b/Å	60.1634(5)
c/Å	40.6595(4)
α/°	90
β/°	90
γ/°	120
Volume/Å^3	127455(2)
Z	8.00004
ρ_{calc}g/cm^3	0.601
μ/mm^{-1}	0.262
F(000)	24816.0
Crystal size/mm^3	0.672 × 0.511 × 0.457
Radiation	CuKα (λ = 1.54184)

2Θ range for data collection/°	6.116 to 101.996
Index ranges	$-59 \leq h \leq 60$, $-60 \leq k \leq 37$, $-22 \leq l \leq 40$
Reflections collected	182559
Independent reflections	45376 [R_{int} = 0.0615, R_{sigma} = 0.0351]
Data/restraints/parameters	45376/14649/2699
Goodness-of-fit on F^2	1.062
Final R indexes [I>=2σ (I)]	R_1 = 0.1305, wR_2 = 0.3249
Final R indexes [all data]	R_1 = 0.1645, wR_2 = 0.3723
Largest diff. peak/hole / e Å^{-3}	0.75/-0.41
Flack parameter	0.6(5)

4 Calculations

Calculations on the semi-empirical (Figure S6) and DFT (Figure S7) level were performed and showed that protonation of the porphyrins seems to be an important part of successful HBC formation. A comparison between mono-porphyrin-HPB **3** and hexa-porphyrin-HPB **10** is given in Figure S6, with representations of the highest occupied molecular orbital (HOMO) distributions. The structures were calculated with the porphyrins in their free-base form **4** and **10** as well as in their mono-protonated form **4**·H^+ and (**10**·H_6)$^{6+}$. In the case of mono-porphyrin-HPB **3** protonation leads to a shift of the HOMO onto the HPB core. For the protonated form of hexa-porphyrin-HPB (**10**·H_6)$^{6+}$ on the other hand, this shift was not observed and only a small portion of the HOMO is located on the HPB core. Therefore, the HOMO location can be considered as the point of preferred oxidation, leading to a successful HBC formation only for mono-porphyrin-HPB **4**.

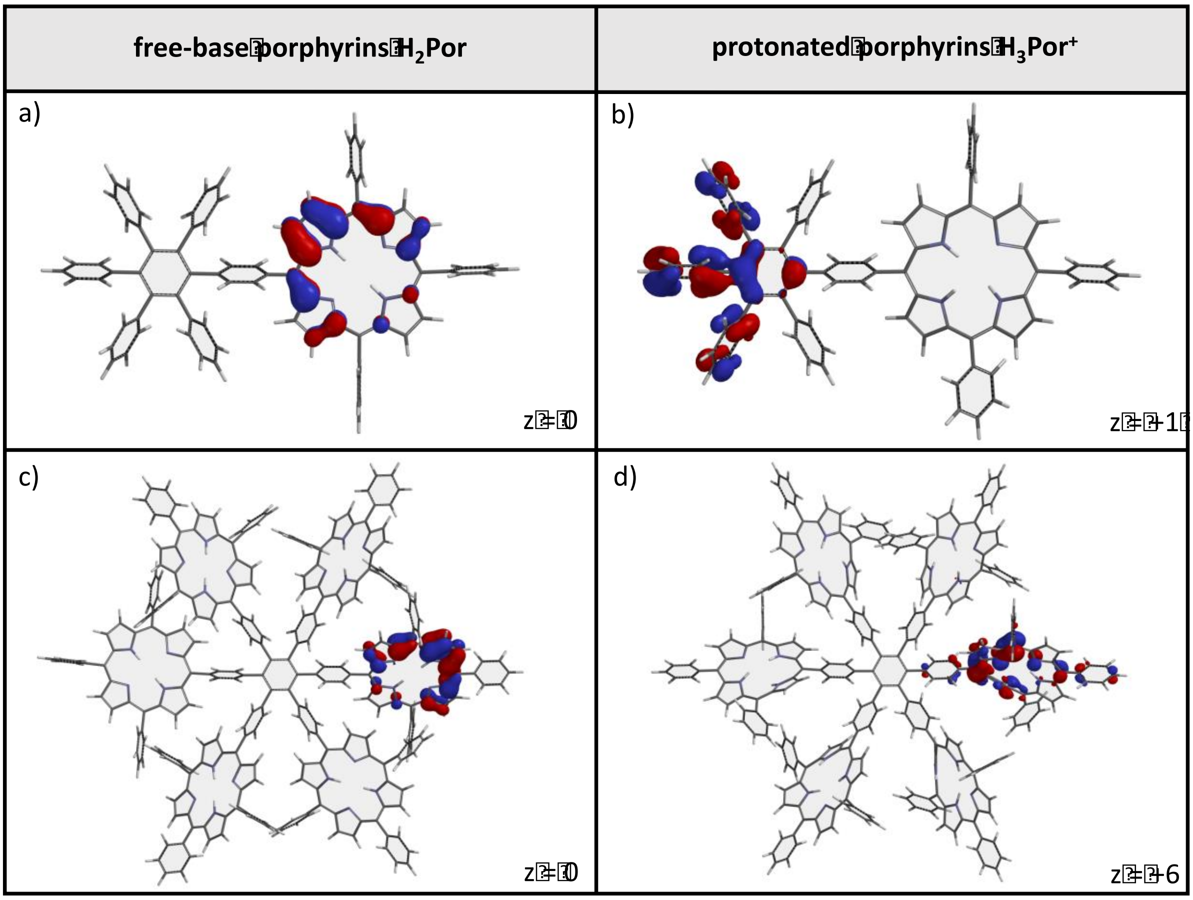

Figure S6. Geometry optimized structures (semi empirical, PM6)[28] of mono-porphyrin-HPB **3** and hexa-porphyrin-HPB **10** with representation of highest occupied molecular orbitals (HOMOs). a), c) Each porphyrin is in its free-base form (H_2Por); b), c) each porphyrin is mono-protonated (H_3Por^+). tBu groups were replaced by H-atoms to save calculation cost. DFT calculations on the level of B3LYP 631Gs were performed as well and showed similar behavior (Figure S7). Due to limited calculation performance, hexa-porphyrin-HPB **10** was not calculated using DFT methods.

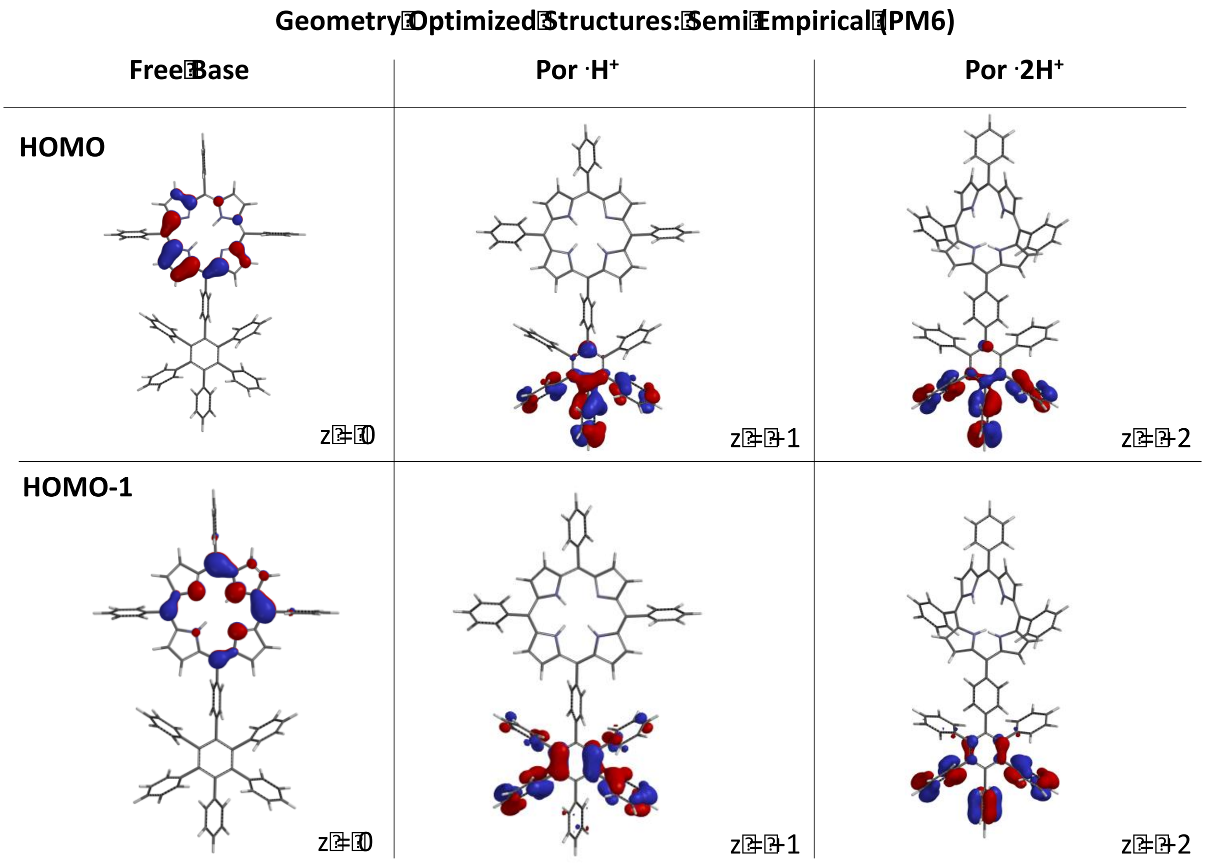

Figure S7. Representation of HOMO and HOMO-1 of mono-porphyrin-HPB **3** in its neutral, mono- and di-protonated form.

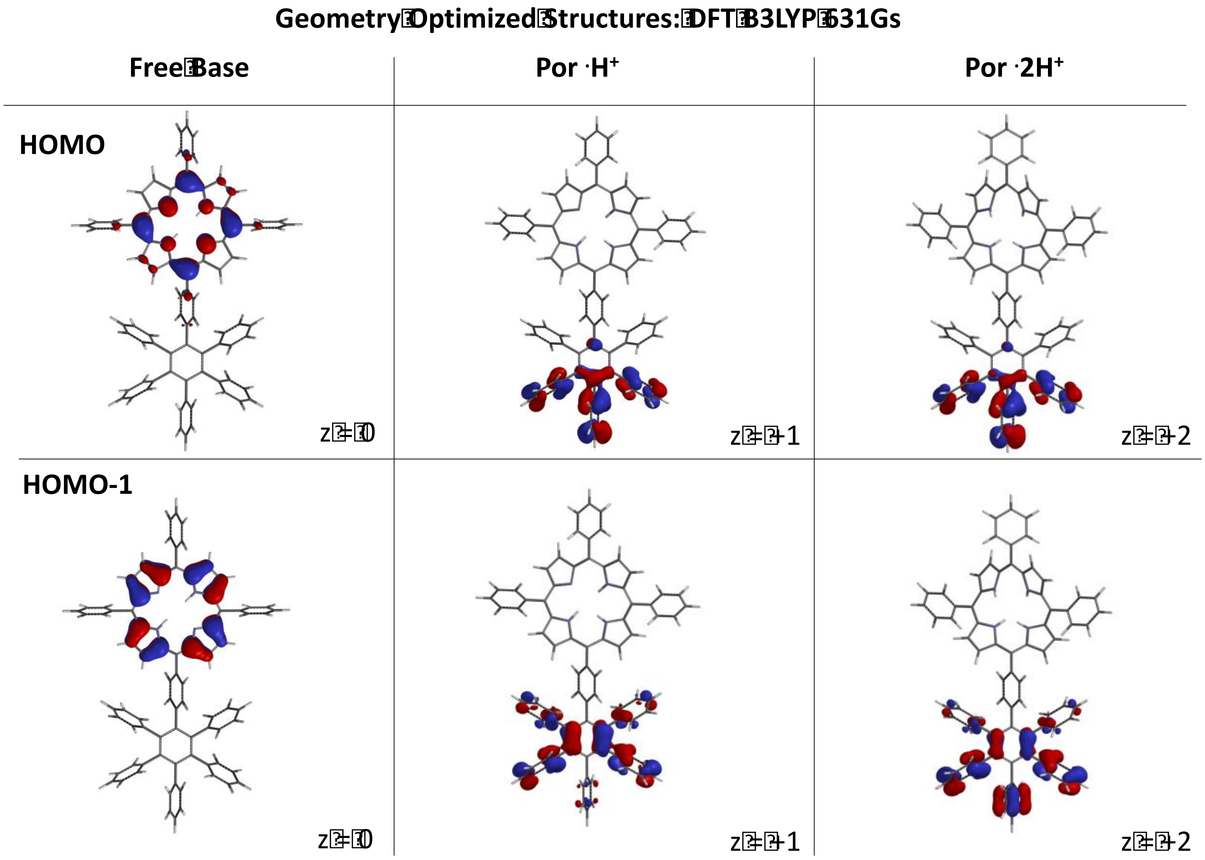

Figure S8. Representation of HOMO and HOMO-1 of mono-porphyrin-HPB **3** in its neutral, mono- and di-protonated form.

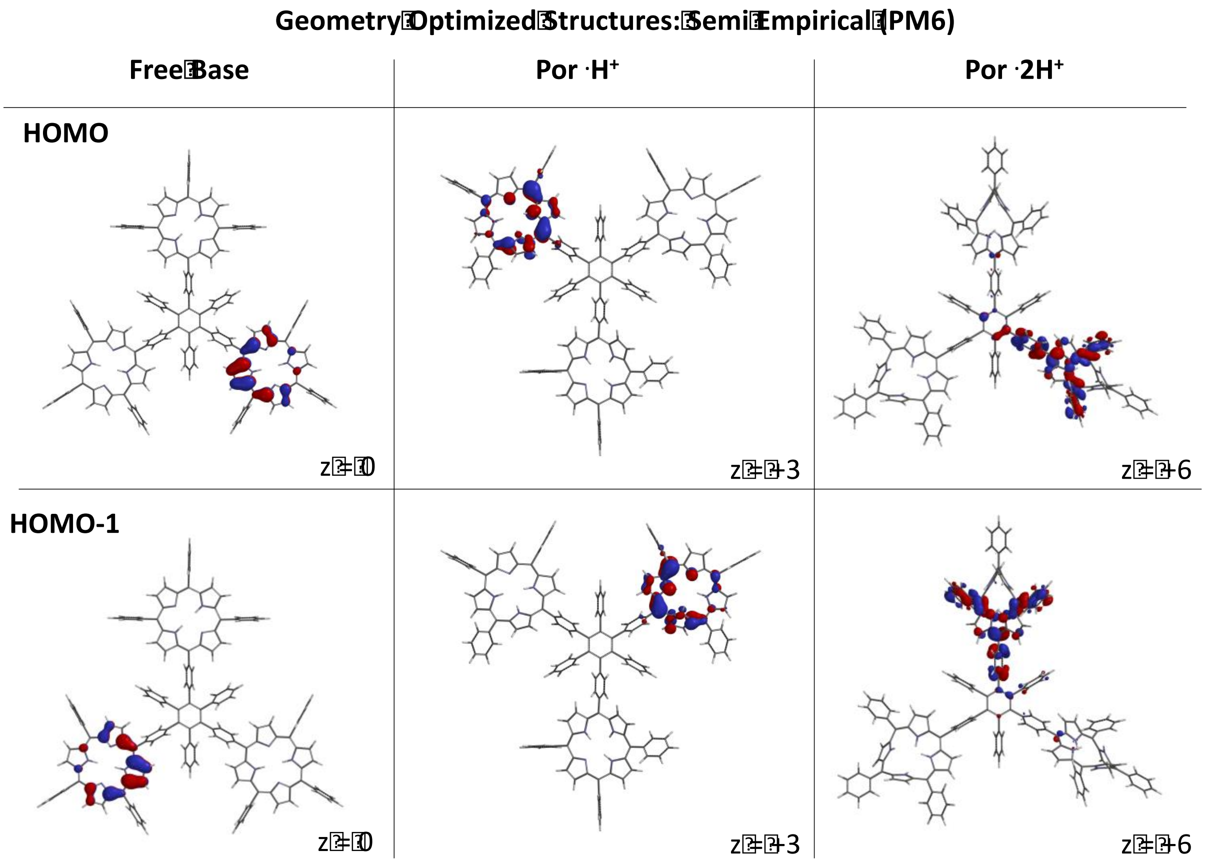

Figure S9. Representation of HOMO and HOMO-1 of $(AB)_3$-tri-porphyrin-HPB **4**. Each porphyrin is in its neutral, mono- and di-protonated form.

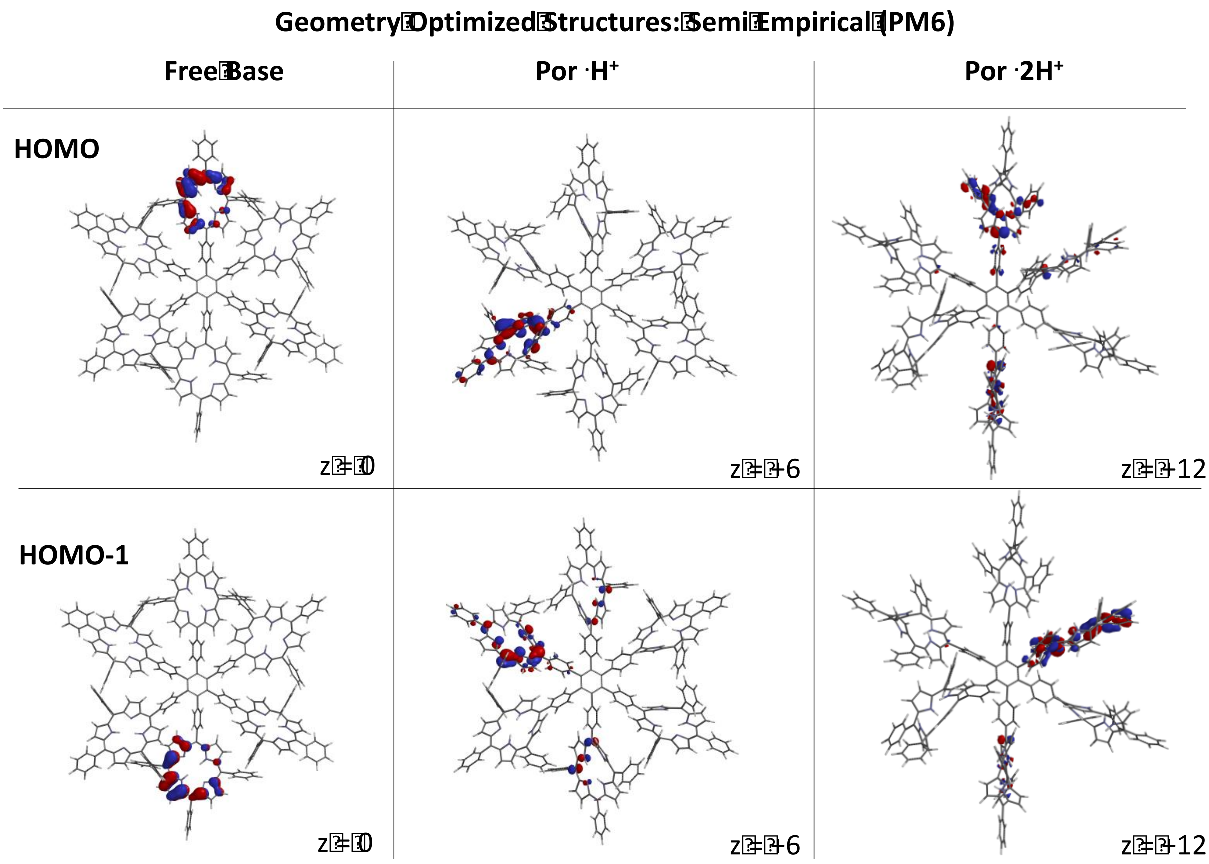

Figure S10. Representation of HOMO and HOMO-1 of hexa-porphyrin-HPB **10**. Each porphyrin is in its neutral, mono- and di-protonated form.

5 Spectral Appendix

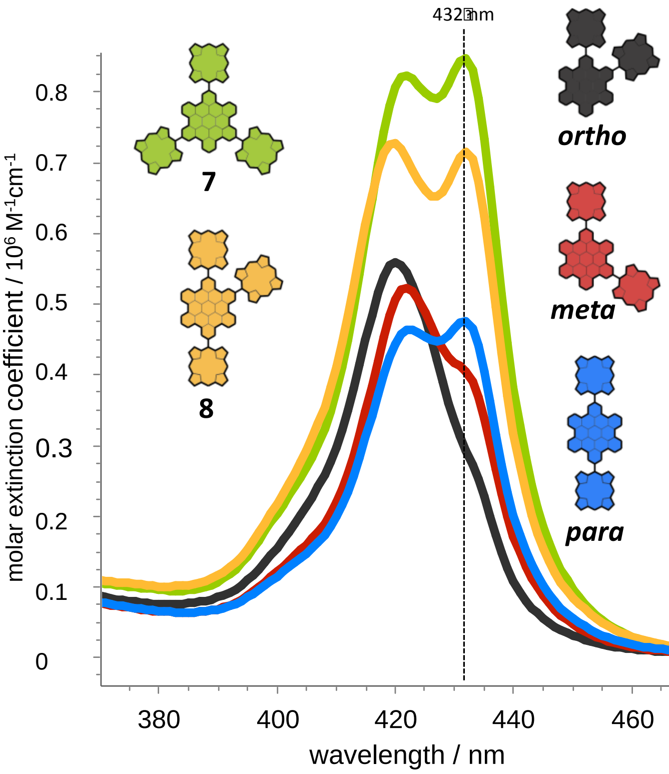

	B-band / nm	FWHM B-band	$e_{B\text{-band}}$ / $10^5 M^{-1}cm^{-1}$	$e_{B\text{-band}}$ per porphyrin / $10^5 M^{-1}cm^{-1}$
ortho	420	23.6	5.59	2.80
meta	422<br>(431)	25.1<br>/	5.23<br>/	2.62<br>/
para	422<br>432	/<br>26.8	4.63<br>4.76	2.32<br>2.38
7	422<br>432	/<br>28.0	7.84<br>8.08	2.61<br>2.69
8	420<br>432	30.7<br>/	7.22<br>7.11	2.41<br>2.37

Figure S11. Comparison of UV/Vis absorption spectra[29] of molecules **7**, **8** and literature known *ortho*-, *meta*-, *para*-bis-porphyrin-HBCs[3] in THF.

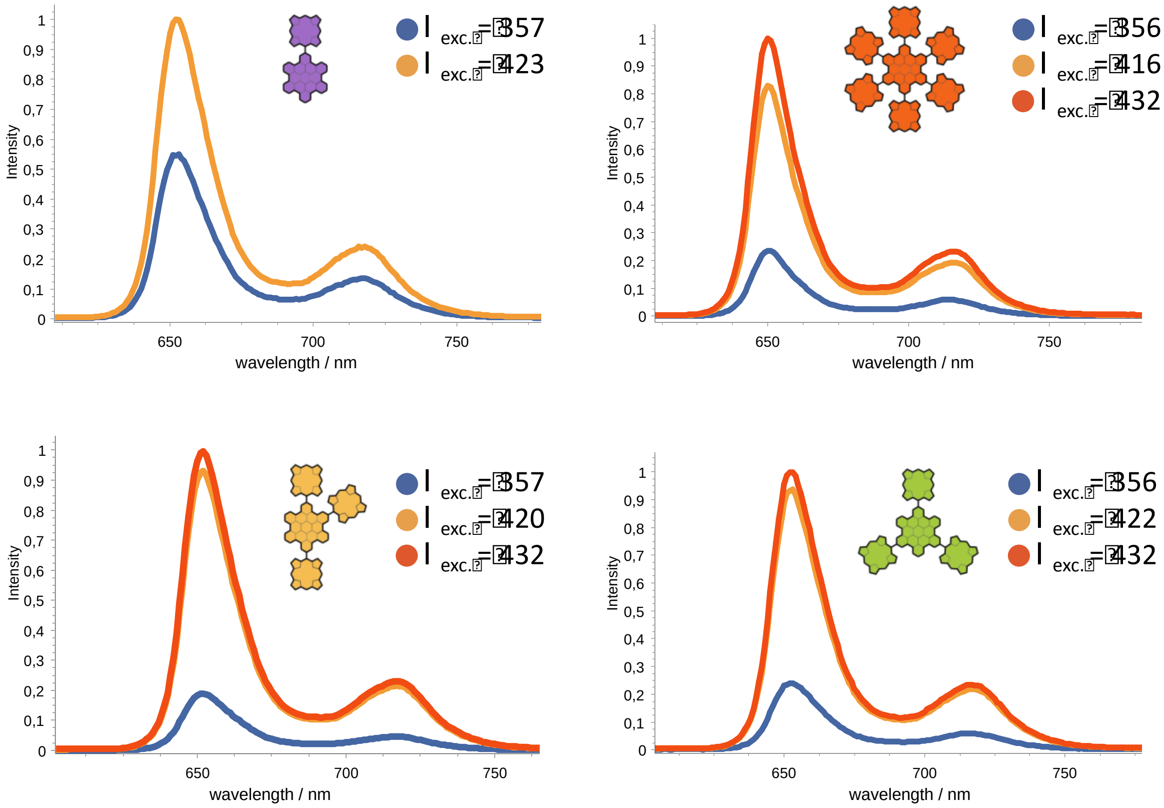

Figure S12. Fluorescence spectra[29] of porphyrin-HBCs **3**, **4**, **5** and **11** in THF.

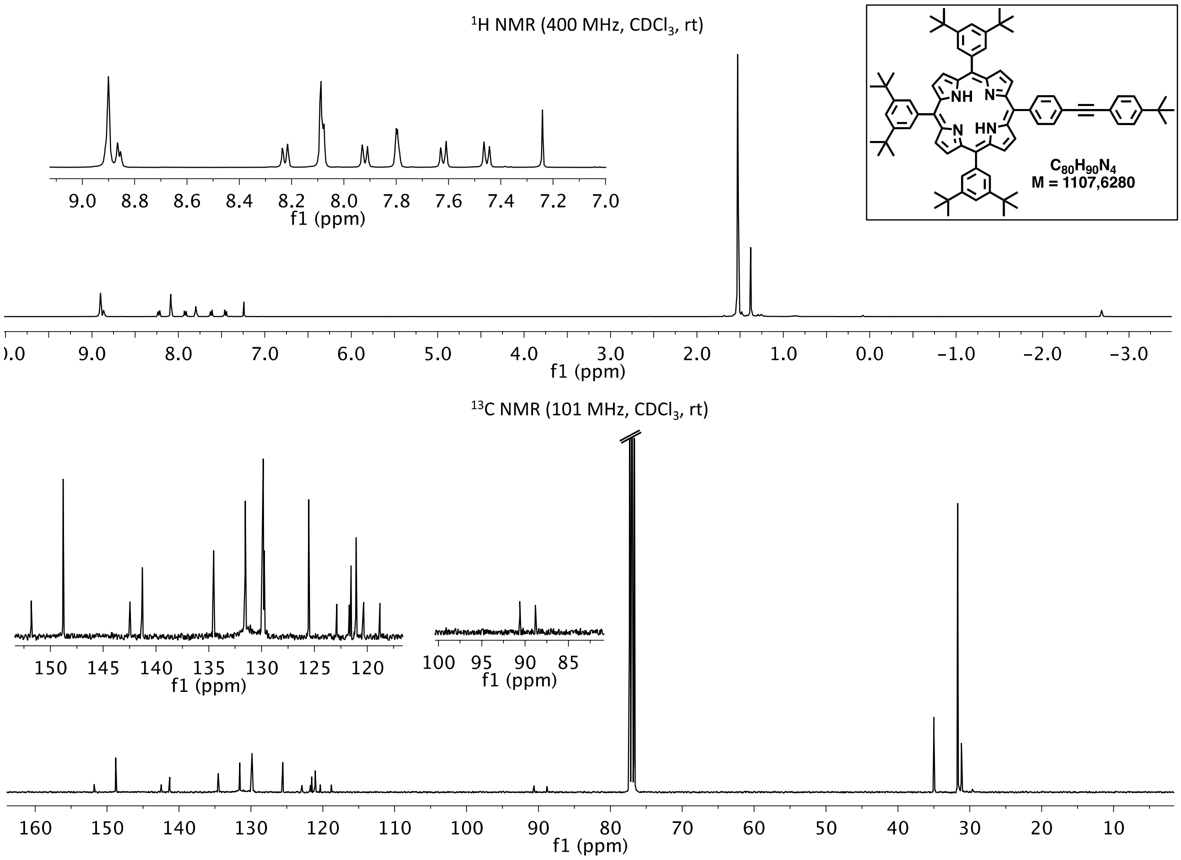

Figure S13. ^{1}H and ^{13}C NMR of free base tolane porphyrin **1**

HRMS (APPI)

Acquisition Parameter

Source Type	APPI	Ion Polarity	Positive	Set Nebulizer	2.5 Bar
Focus	Not active	Set Capillary	700 V	Set Dry Heater	200 °C
Scan Begin	300 m/z	Set End Plate Offset	-500 V	Set Dry Gas	1.5 l/min
Scan End	2900 m/z	Set Charging Voltage	0 V	Set Divert Valve	Waste
		Set Corona	0 nA	Set APCI Heater	450 °C

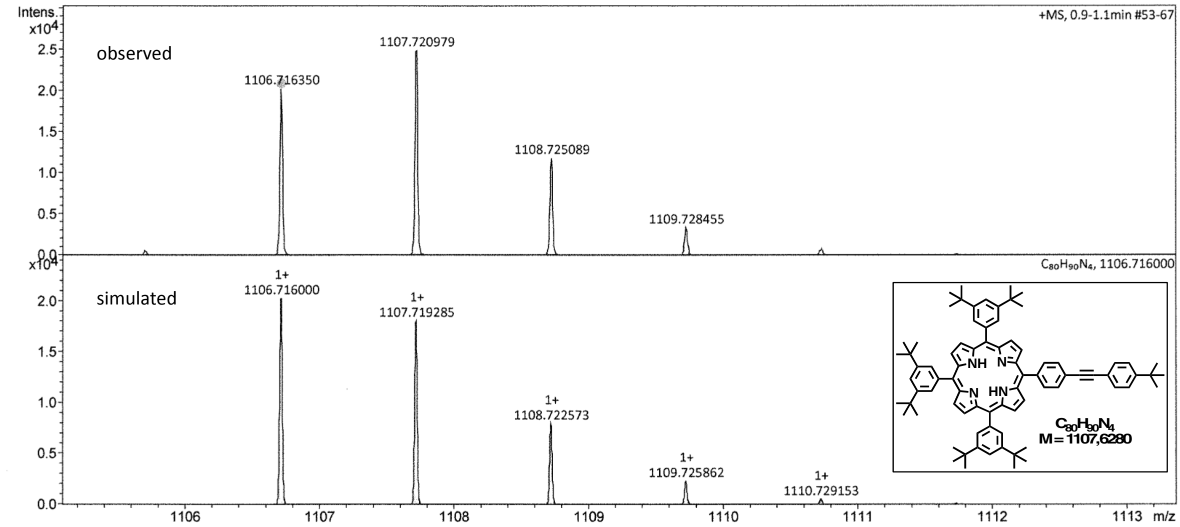

Meas. m/z	#	Ion Formula	m/z	err [ppm]	mSigma	# mSigma	Score	rdb	e⁻ Conf	N-Rule
1106.716350	1	C80H90N4	1106.716000	-0.316	104.4	1	100.00	38.0	odd	ok
	2	C79H94O4	1106.714663	-1.524	111.8	2	31.94	33.0	odd	ok
	3	C12H3O60	1106.717804	1.313	419.7	3	0.00	11.5	even	ok
	4	C10HN3O59	1106.716461	0.100	425.0	4	0.00	12.0	odd	ok

Figure S14. HRMS (APPI) of free base tolane porphyrin **1.**

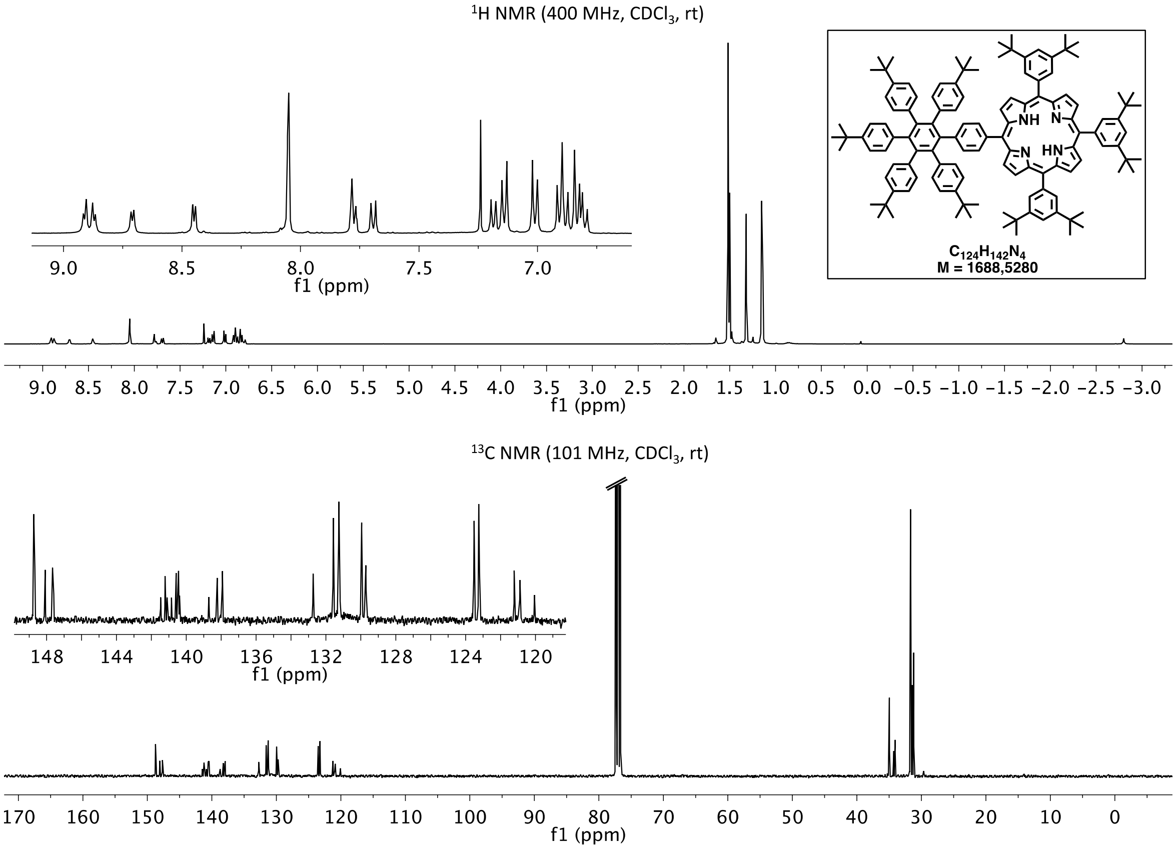

Figure S15. ^{1}H and ^{13}C NMR of mono-porphyrin-HPB **3**.

HRMS(APPI)

Acquisition Parameter

Source Type	APPI	Ion Polarity	Positive	Set Nebulizer	2.5 Bar
Focus	Not active	Set Capillary	700 V	Set Dry Heater	200 °C
Scan Begin	300 m/z	Set End Plate Offset	-500 V	Set Dry Gas	1.5 l/min
Scan End	2900 m/z	Set Charging Voltage	0 V	Set Divert Valve	Waste
		Set Corona	0 nA	Set APCI Heater	450 °C

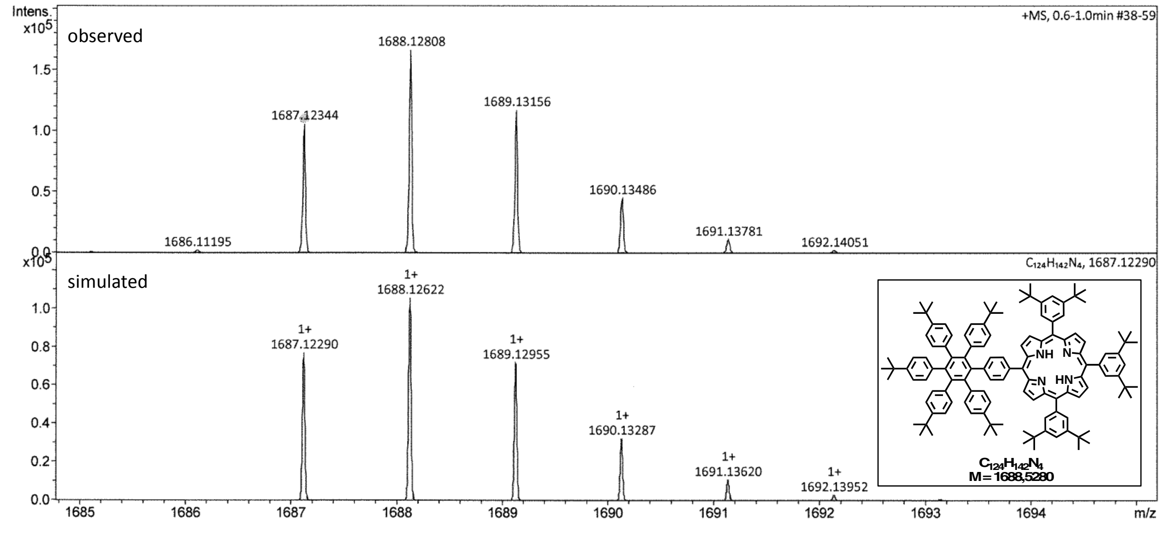

Meas. m/z	#	Ion Formula	m/z	err [ppm]	mSigma	# mSigma	Score	rdb	e⁻ Conf	N-Rule
1687.12344	1	C124H142N4	1687.12290	-0.32	45.0	1	100.00	56.0	odd	ok
	2	C125H19N4O7	1687.12483	0.82	48.4	2	56.14	118.5	even	ok
	3	C123H146O4	1687.12156	-1.11	49.8	3	38.42	51.0	odd	ok
	4	C124H23O11	1687.12349	0.03	52.5	4	99.22	113.5	even	ok
	5	C122H21N3O10	1687.12215	-0.77	53.5	5	50.74	114.0	odd	ok

Figure S16. HRMS (APPI) of mono-porphyrin-HPB **3**.

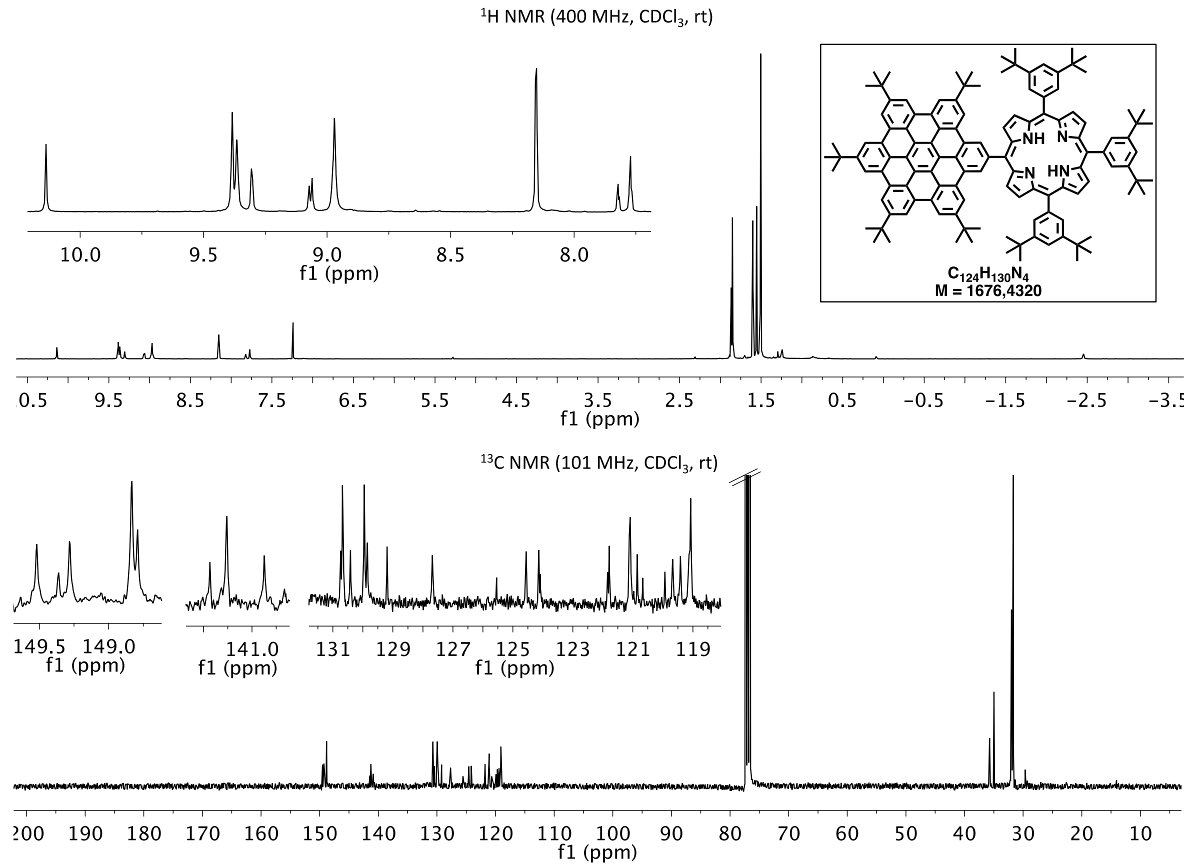

Figure S17. ^{1}H and ^{13}C NMR of mono-porphyrin-HBC **6**.

HRMS (APPI)

Acquisition Parameter

Source Type	APPI	Ion Polarity	Positive	Set Nebulizer	2.5 Bar
Focus	Not active	Set Capillary	700 V	Set Dry Heater	200 °C
Scan Begin	300 m/z	Set End Plate Offset	-500 V	Set Dry Gas	1.5 l/min
Scan End	2900 m/z	Set Charging Voltage	0 V	Set Divert Valve	Waste
		Set Corona	0 nA	Set APCI Heater	450 °C

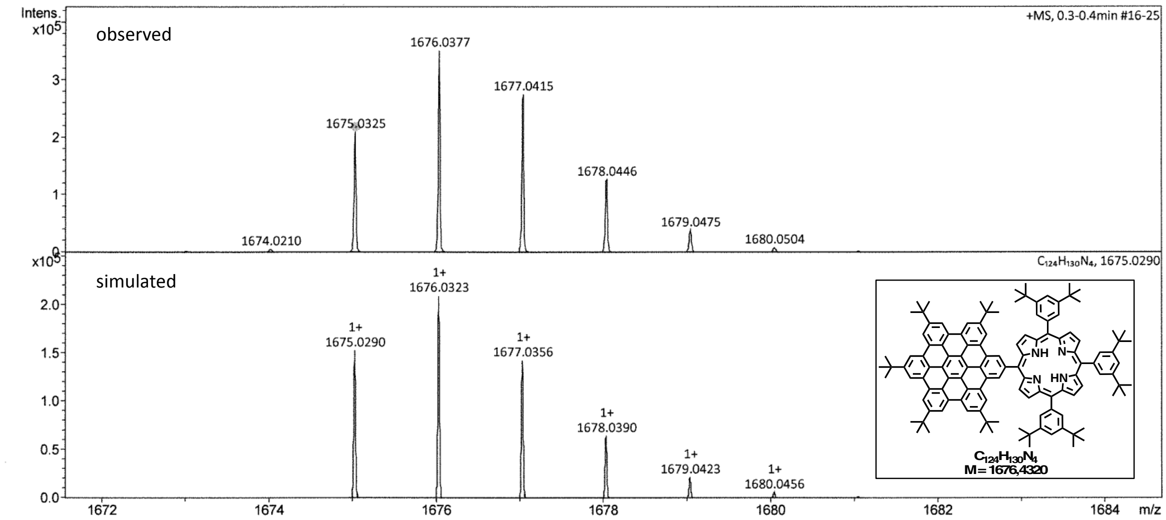

Meas. m/z	#	Ion Formula	m/z	err [ppm]	mSigma	# mSigma	Score	rdb	e⁻ Conf	N-Rule
1675.0325	1	C124H130N4	1675.0290	-2.1	72.1	1	59.66	62.0	odd	ok
	2	C124H11O11	1675.0296	-1.7	74.0	2	100.00	119.5	even	ok

Figure S18. HRMS (APPI) of mono-porphyrin-HBC **6**.

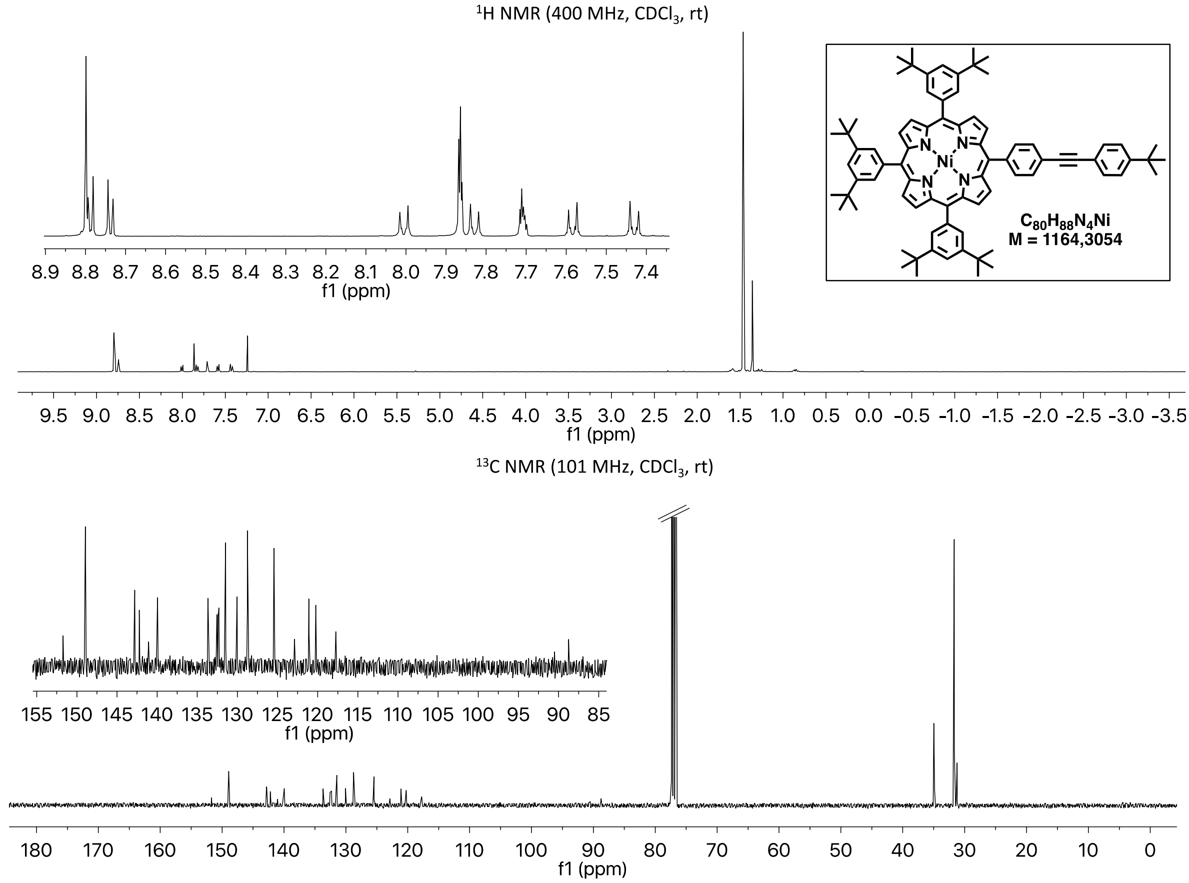

Figure S19. ^1H and ^{13}C NMR of nickel-tolane-porphyrin **1·Ni**.

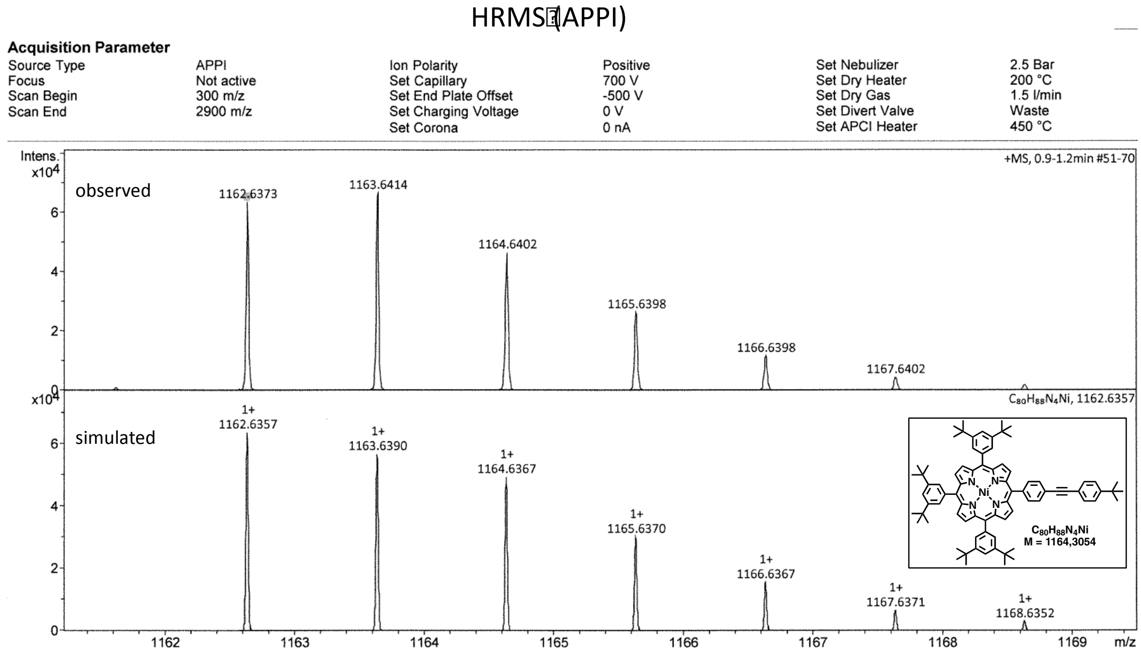

Meas. m/z	#	Ion Formula	m/z	err [ppm]	mSigma	# mSigma	Score	rdb	e⁻ Conf	N-Rule
1162.6373	1	C80H88N4Ni	1162.6357	-1.3	66.4	1	52.54	39.0	odd	ok
	2	C82H90NNiO	1162.6370	-0.2	69.1	2	100.00	38.5	even	ok

Figure S20. HRMS (APPI) of nickel-tolane-porphyrin **1·Ni**.

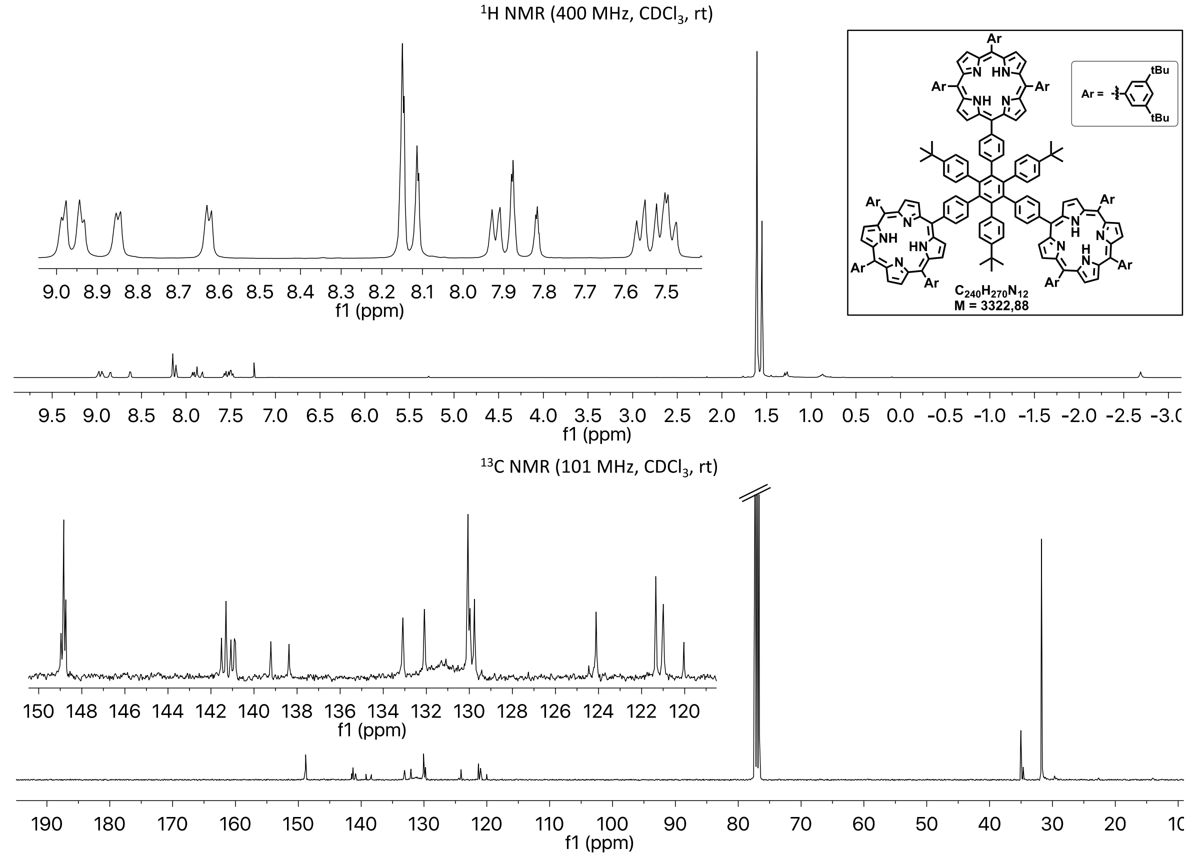

Figure S21. ^{1}H and ^{13}C NMR of (AB)$_3$-tri-porphyrin-HPB **4**.

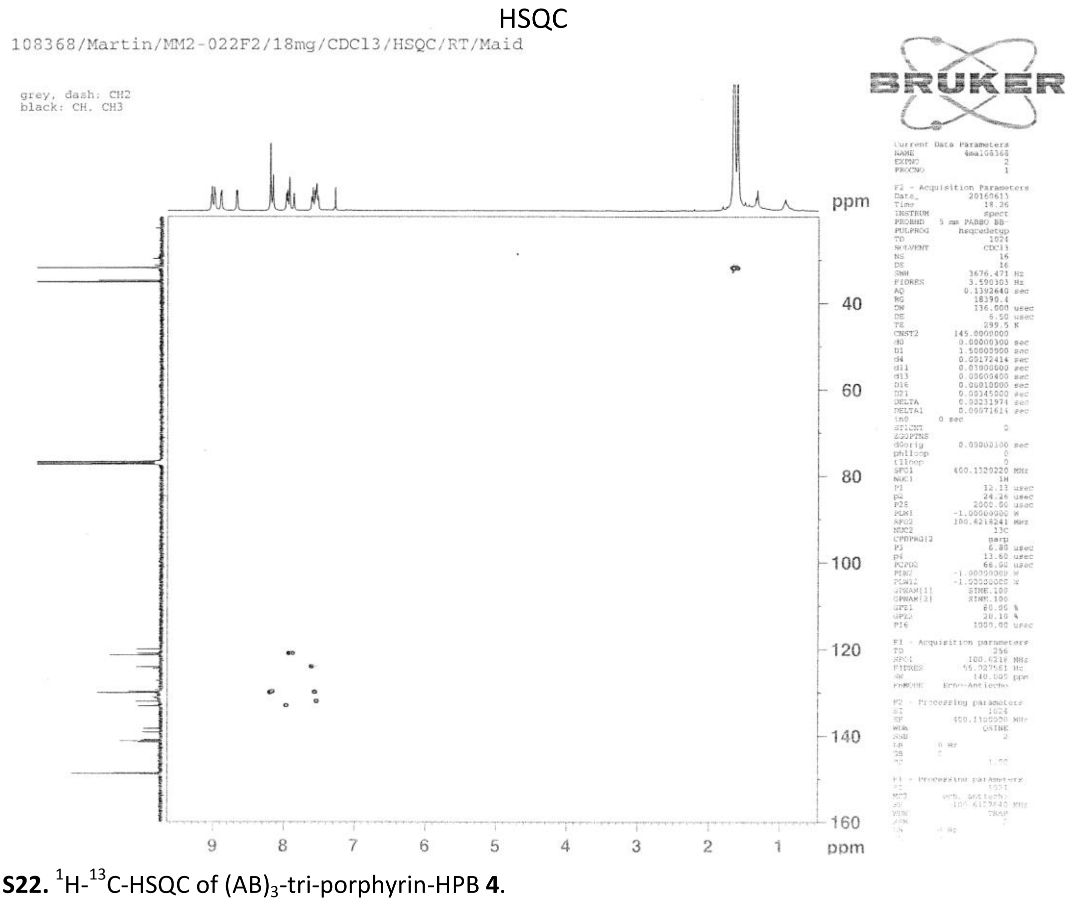

Figure S22. ^{1}H-^{13}C-HSQC of (AB)$_3$-tri-porphyrin-HPB **4**.

HMBC

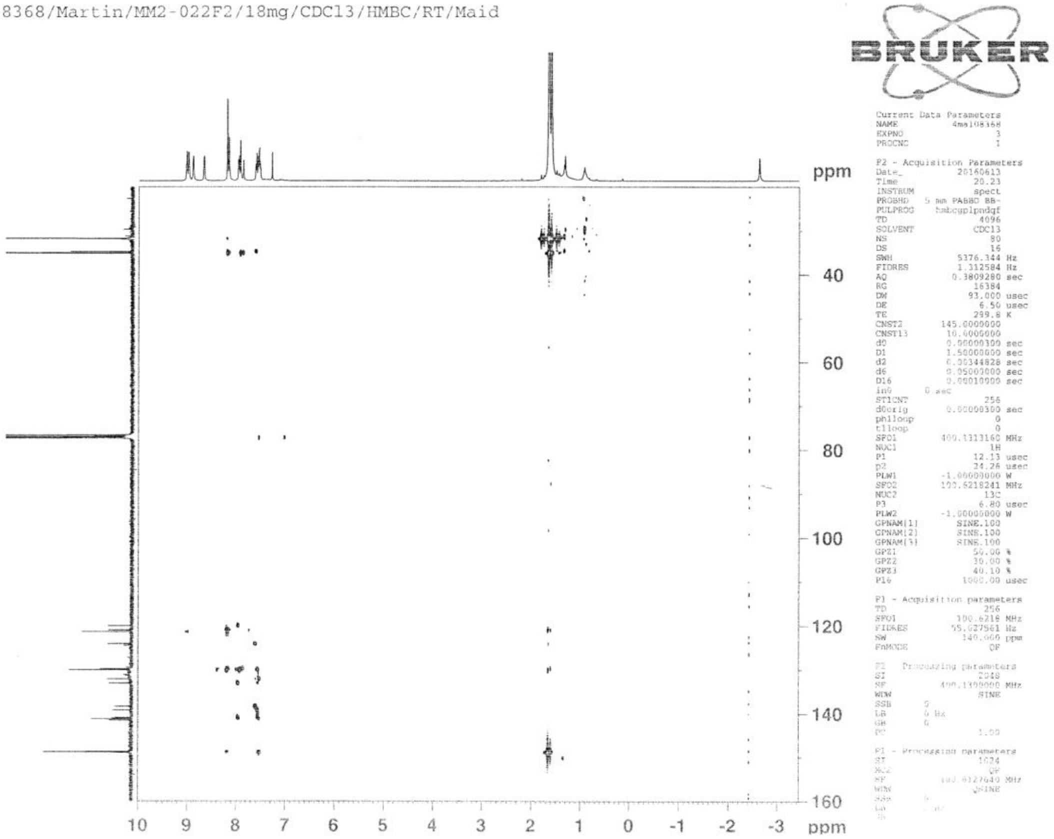

Figure S23. ^{1}H-^{13}C-HMBC of $(AB)_3$-tri-porphyrin-HPB **4**.

HRMS (ESI)

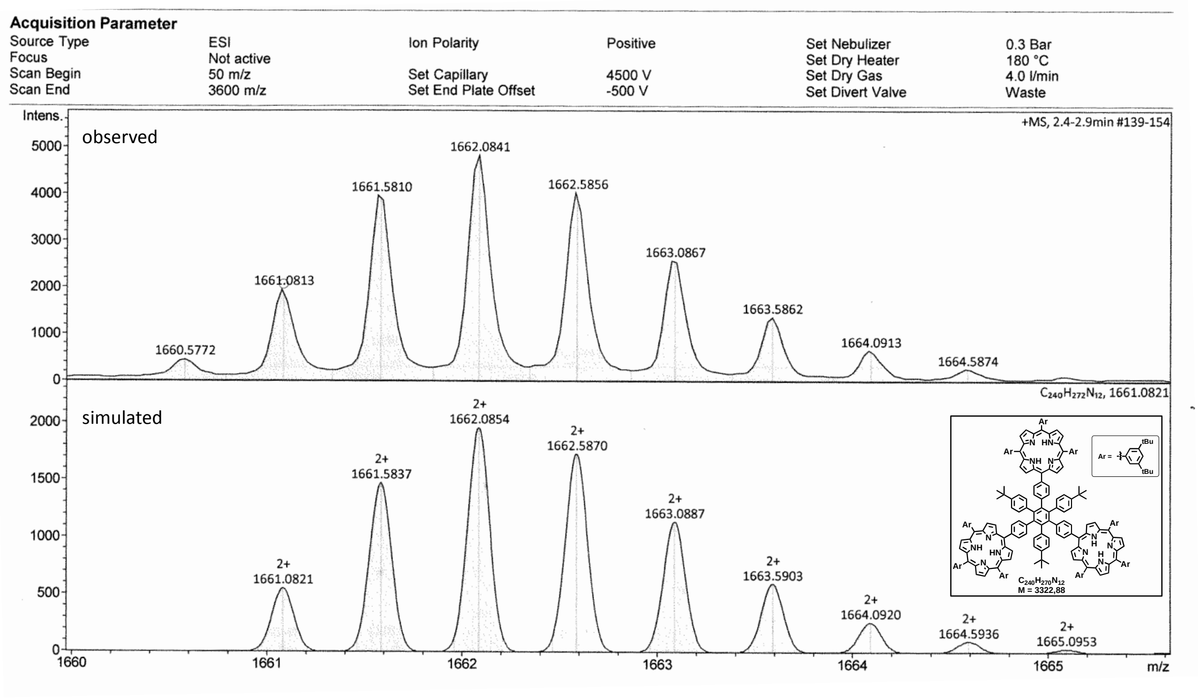

Meas. m/z	#	Ion Formula	m/z	err [ppm]	Mean err [ppm]	rdb	N-Rule	e⁻ Conf	mSigma	Std I	Std Mean m/z	Std I VarNorm	Std m/z Diff	Std Comb Dev
1661.081251	1	C240H272N12	1661.082100	0.5	328.8	111.0	ok	even	53.0	39.4	n.a.	n.a.	n.a.	n.a.

Figure S24. HRMS (ESI) of $(AB)_3$-tri-porphyrin-HPB **4**.

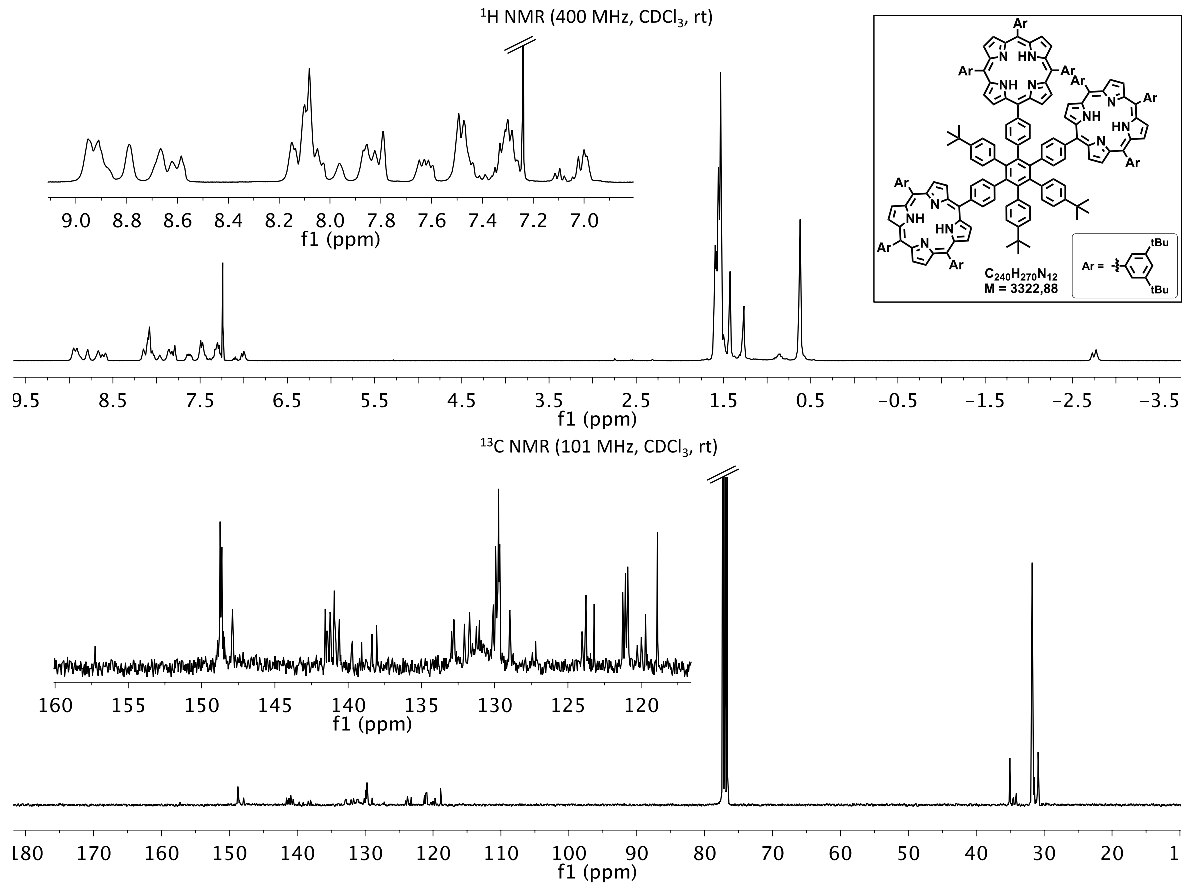

Figure S25. ^{1}H and ^{13}C NMR of A_2B_2AB-tri-porphyrin-HPB **5**.

HRMS (ESI)

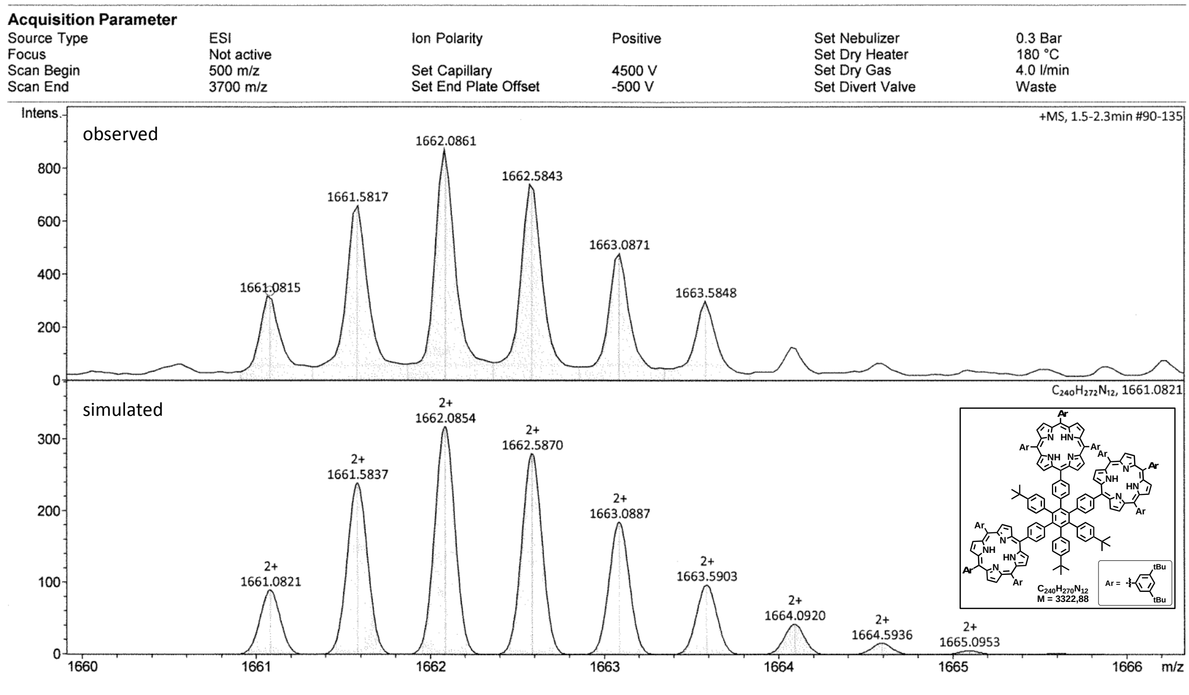

Meas. m/z	#	Ion Formula	m/z	err [ppm]	Mean err [ppm]	rdb	N-Rule	e⁻ Conf	mSigma	Std I	Std Mean m/z	Std I VarNorm	Std m/z Diff	Std Comb Dev
1661.081495	1	C240H272N12	1661.082100	0.4	901.1	111.0	ok	even	58.7	46.6	n.a.	n.a.	n.a.	n.a.

Figure S26. HRMS (ESI) of A_2B_2AB-tri-porphyrin-HPB **5**.

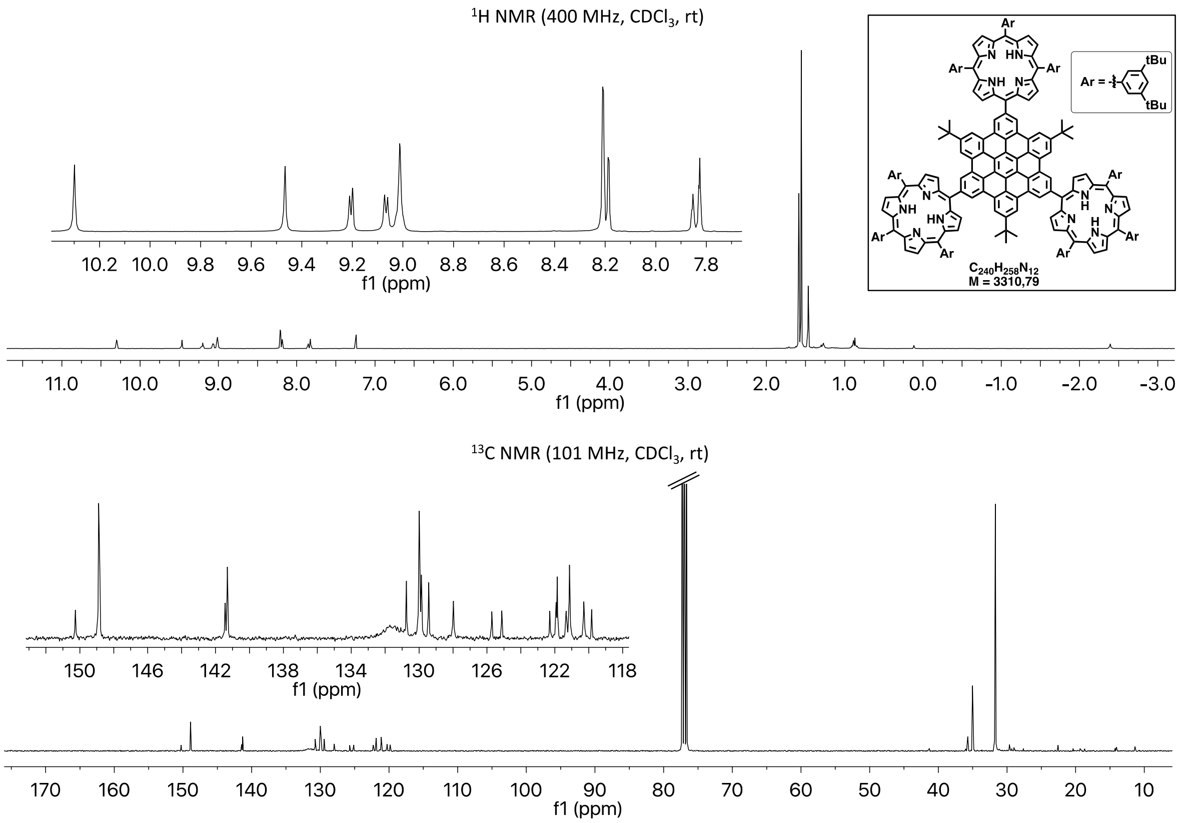

Figure S27. ^{1}H and ^{13}C NMR of (AB)$_3$-tri-porphyrin-HBC **7**.

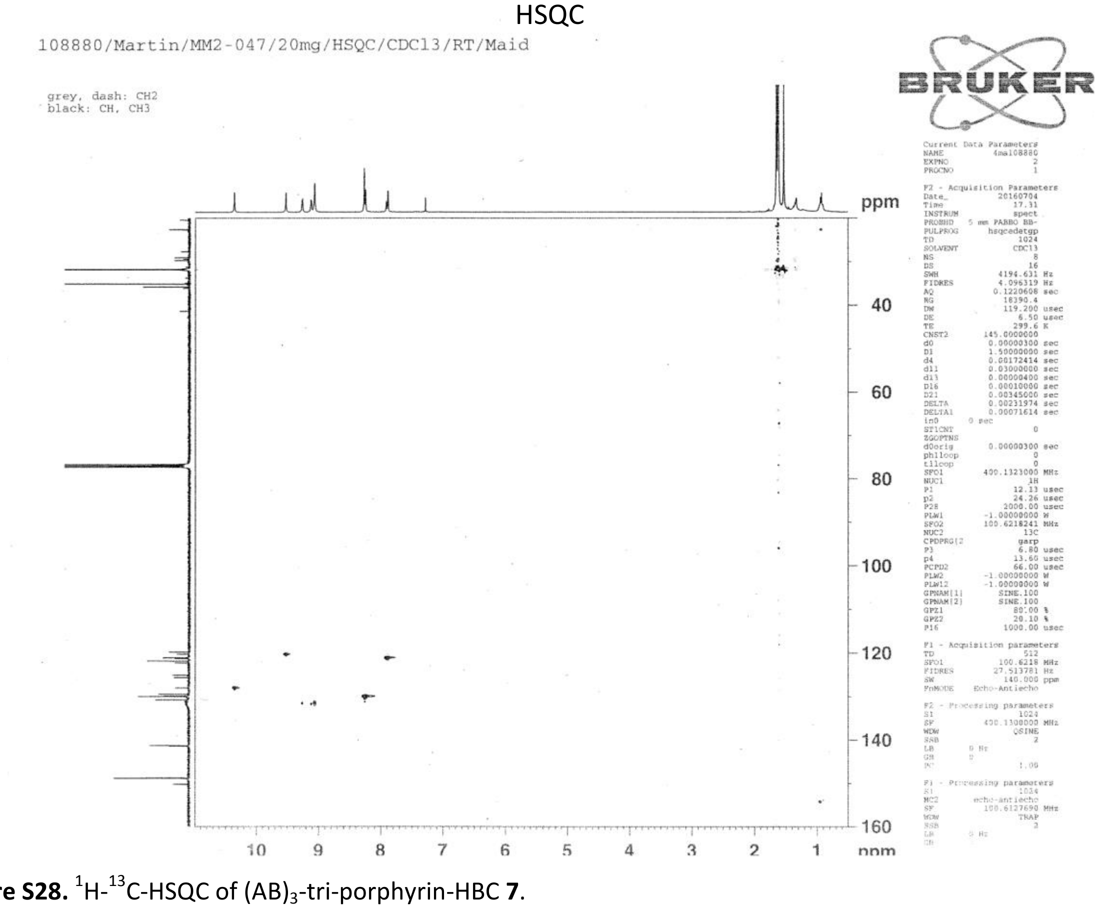

Figure S28. ^{1}H-^{13}C-HSQC of (AB)$_3$-tri-porphyrin-HBC **7**.

HMBC

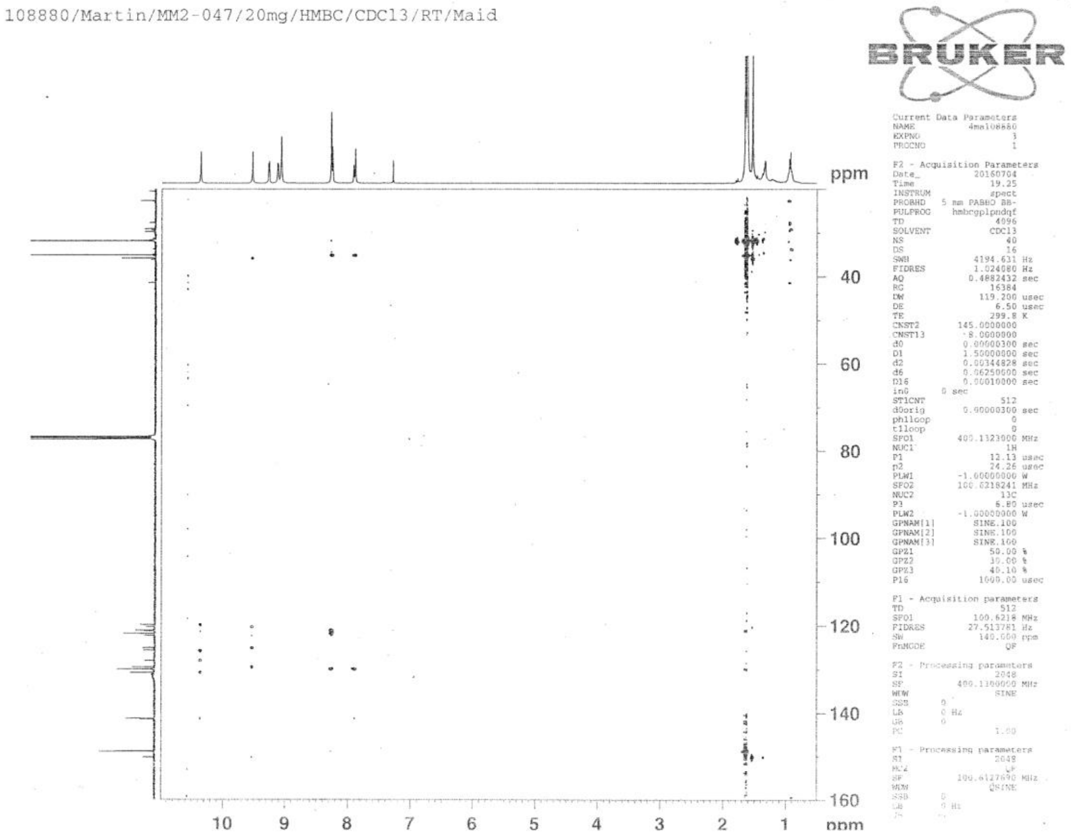

Figure S29. ^{1}H-^{13}C-HMBC of (AB)$_3$-tri-porphyrin-HBC **7**.

HRMS (ESI)

Acquisition Parameter

Source Type	ESI	Ion Polarity	Positive	Set Nebulizer	0.3 Bar
Focus	Not active			Set Dry Heater	180 °C
Scan Begin	500 m/z	Set Capillary	5800 V	Set Dry Gas	4.0 l/min
Scan End	3800 m/z	Set End Plate Offset	-500 V	Set Divert Valve	Waste

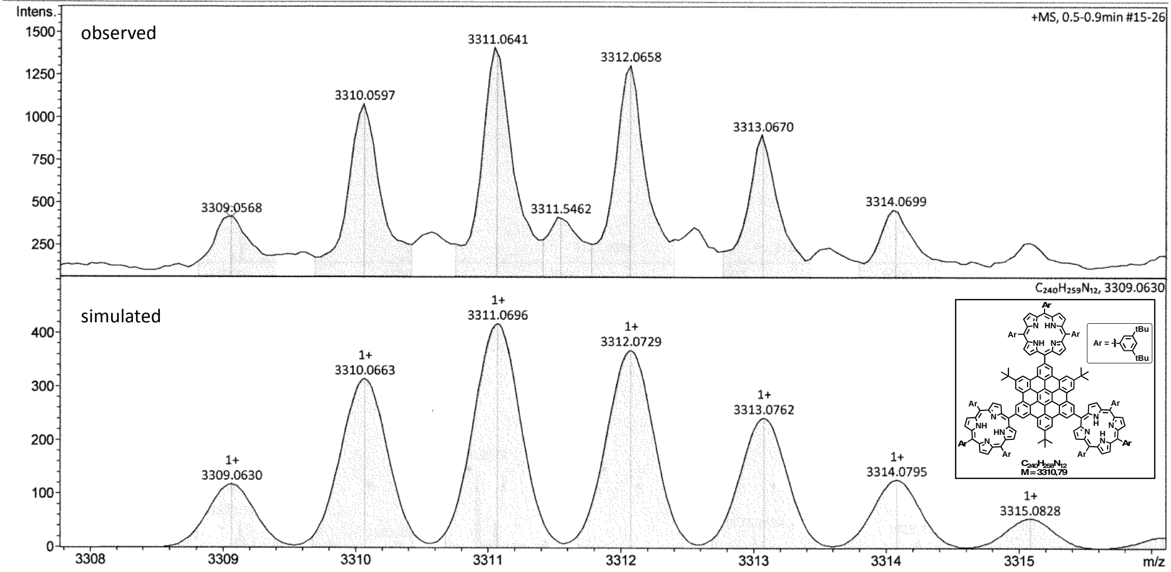

Meas. m/z	#	Ion Formula	m/z	err [ppm]	Mean err [ppm]	rdb	N-Rule	e⁻ Conf	mSigma	Std I	Std Mean m/z	Std I VarNorm	Std m/z Diff	Std Comb Dev
3309.056829	1	C240H259N12	3309.063023	1.9	453.6	117.5	ok	even	54.3	42.8	n.a.	n.a.	n.a.	n.a.

Figure S30. HRMS (ESI) of (AB)$_3$-tri-porphyrin-HBC **7**.

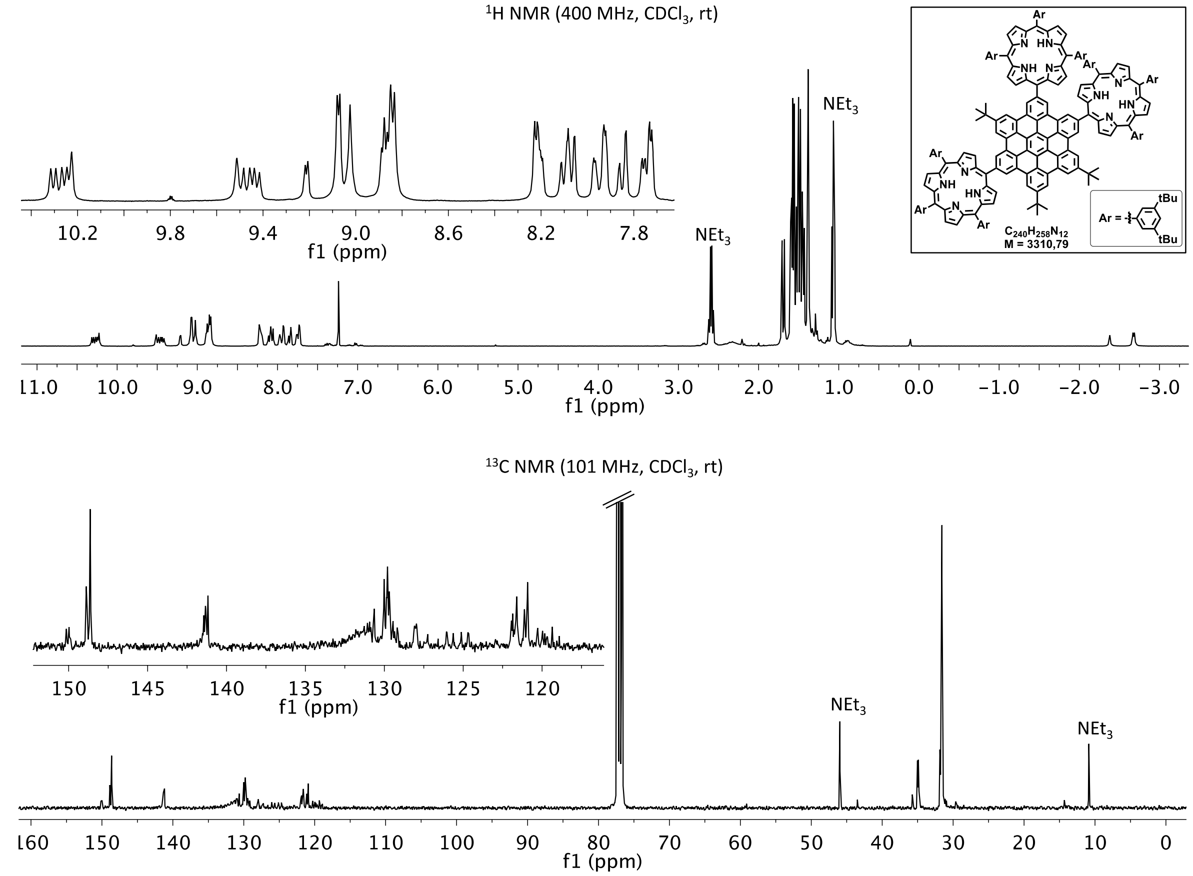

Figure S31. ^{1}H and ^{13}C NMR of A_2B_2AB-tri-porphyrin-HBC **8**.

HRMS (MALDI)

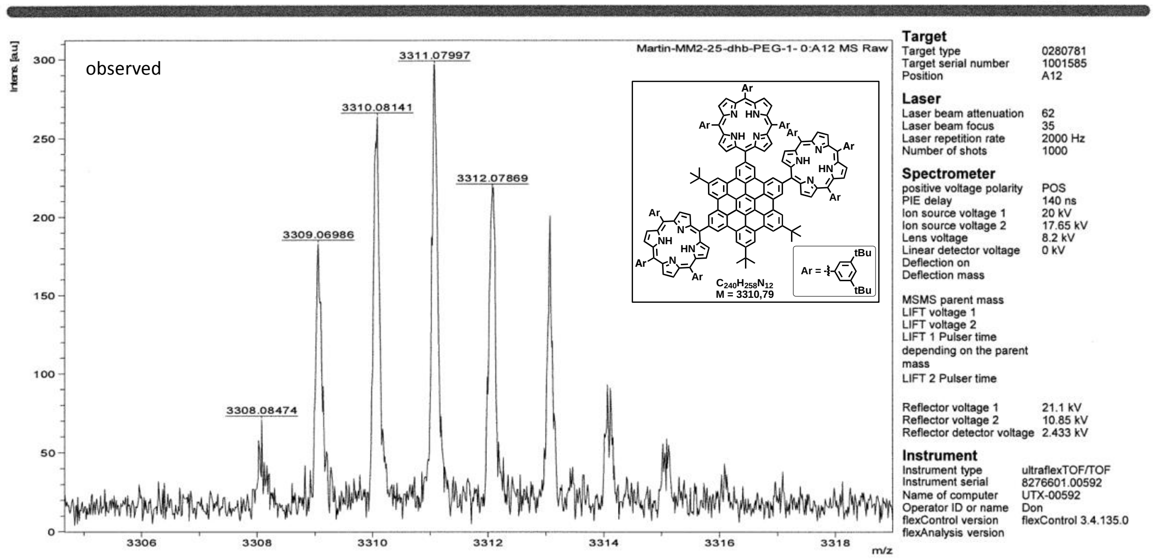

SmartFormula

Formula	Mass	Error	mSigma	DblEq	N rule	Electron Configuration
C 240 H 259 N 12	3,309.0630	2.0675	485.9238	117.50	ok	even

Figure S32. HRMS (MALDI) of A_2B_2AB-tri-porphyrin-HBC **8**.

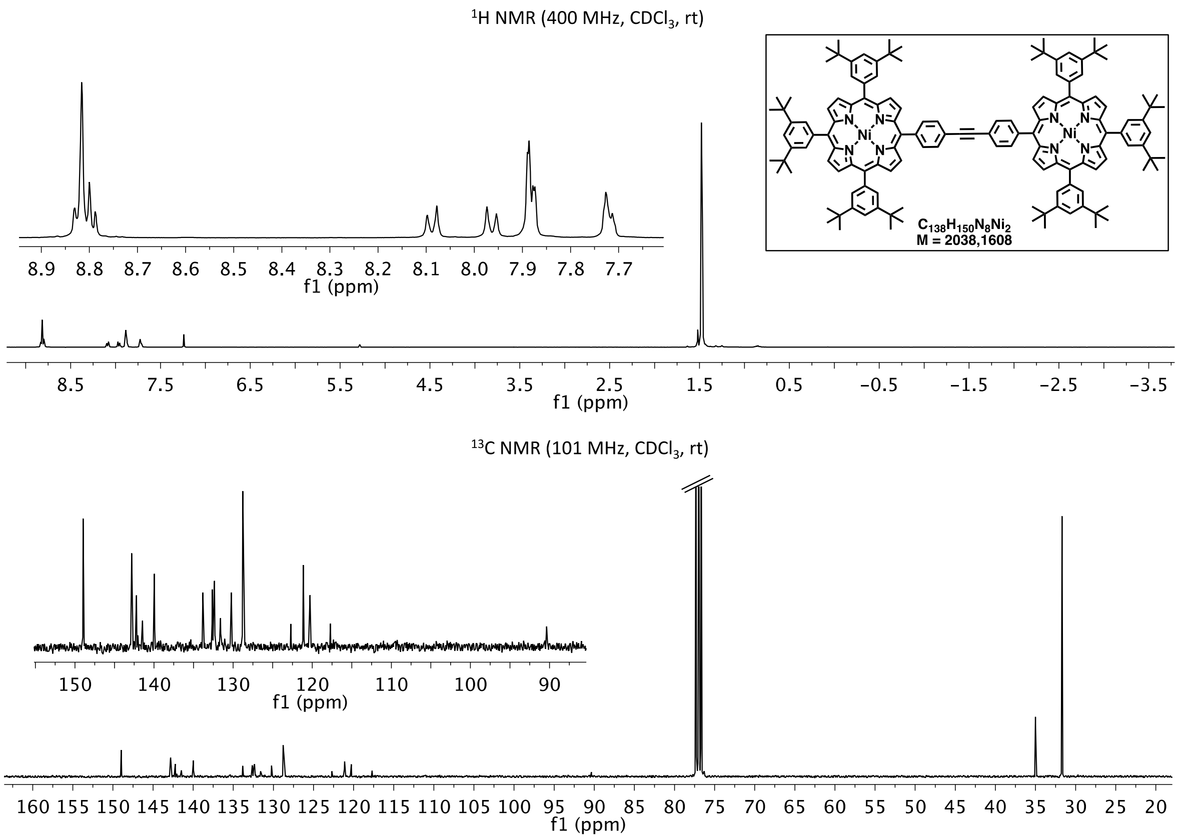

Figure S33. ^{1}H and ^{13}C NMR of nickel porphyrin dimer **9·Ni$_2$**.

HRMS (APPI)

Acquisition Parameter

Source Type	APPI	Ion Polarity	Positive	Set Nebulizer	2.5 Bar
Focus	Not active	Set Capillary	700 V	Set Dry Heater	200 °C
Scan Begin	300 m/z	Set End Plate Offset	-500 V	Set Dry Gas	1.5 l/min
Scan End	2900 m/z	Set Charging Voltage	0 V	Set Divert Valve	Waste
		Set Corona	0 nA	Set APCI Heater	450 °C

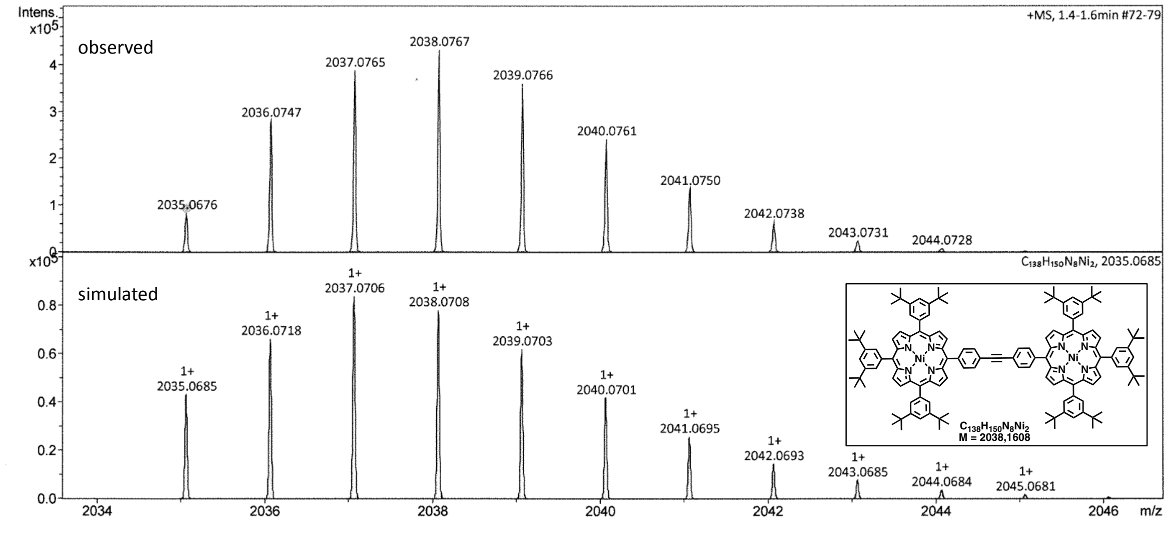

Meas. m/z	#	Ion Formula	m/z	err [ppm]	mSigma	# mSigma	Score	rdb	e⁻ Conf	N-Rule
2035.0676	1	C138H150N8Ni2	2035.0685	0.4	117.8	1	100.00	68.0	odd	ok

Figure S34. HRMS (APPI) of nickel porphyrin dimer **9·Ni$_2$**.

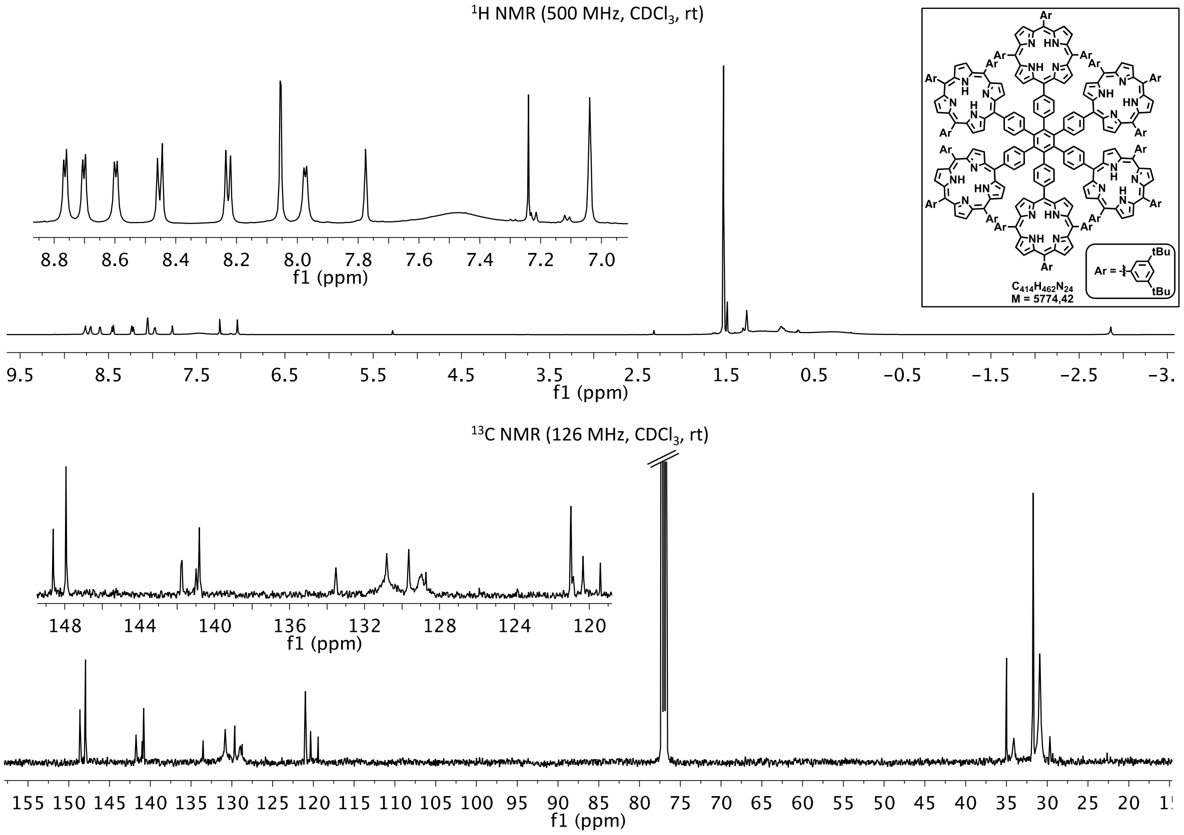

Figure S35. ^{1}H and ^{13}C NMR of hexa-porphyrin-HPB **10**.

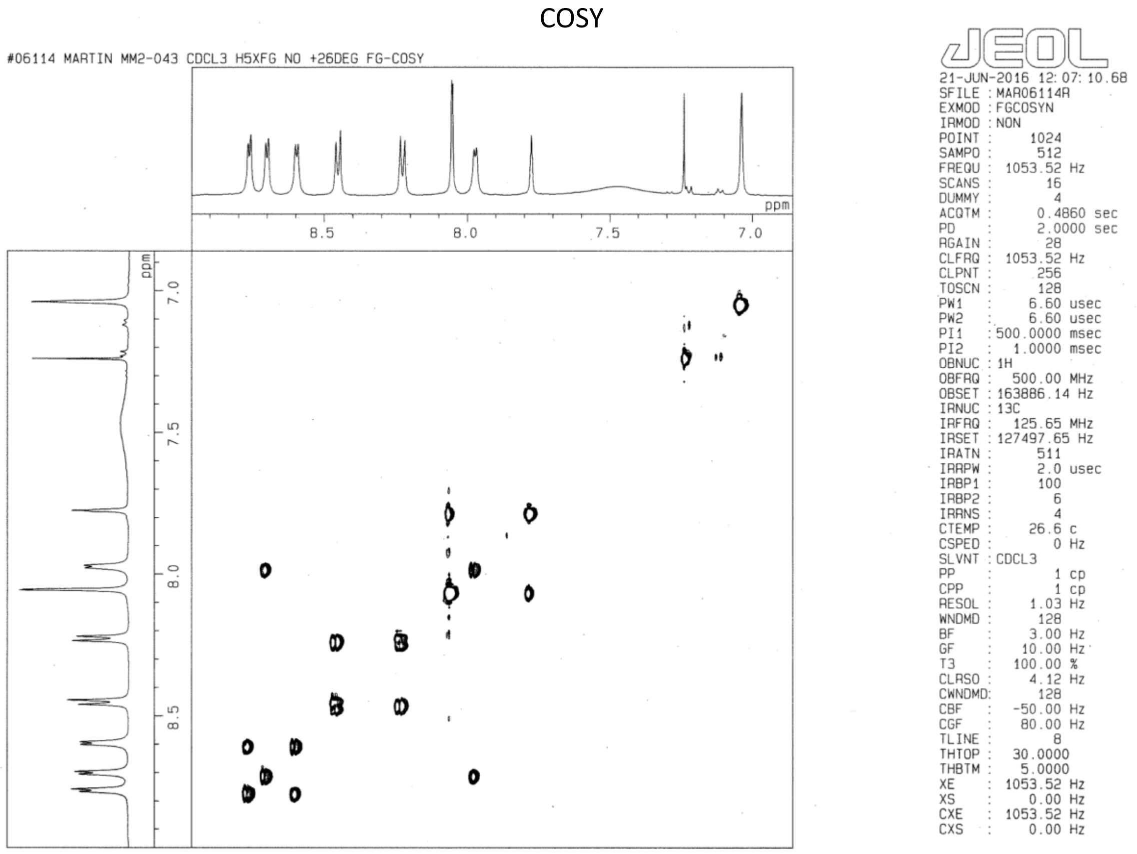

Figure S36. ^{1}H-^{1}H COSY of hexa-porphyrin-HPB **10**.

HSQC

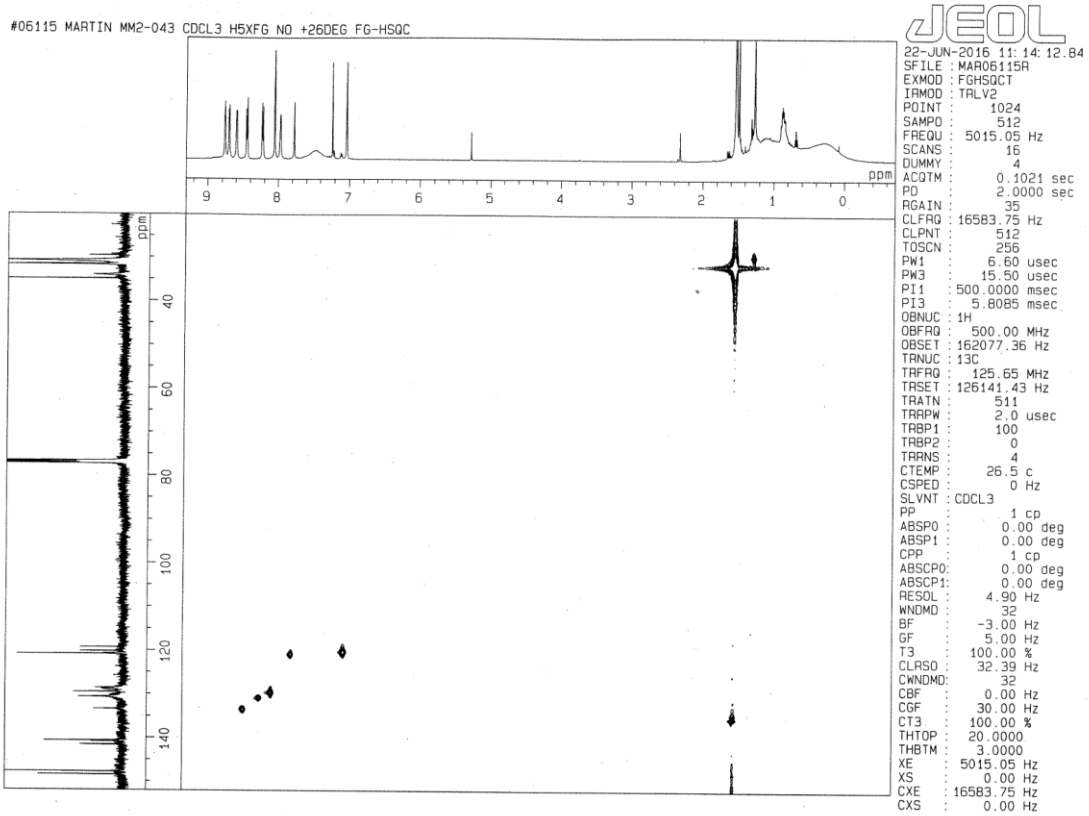

Figure S37. ^{1}H-^{13}C-HSQC of hexa-porphyrin-HPB **10**.

HMBC

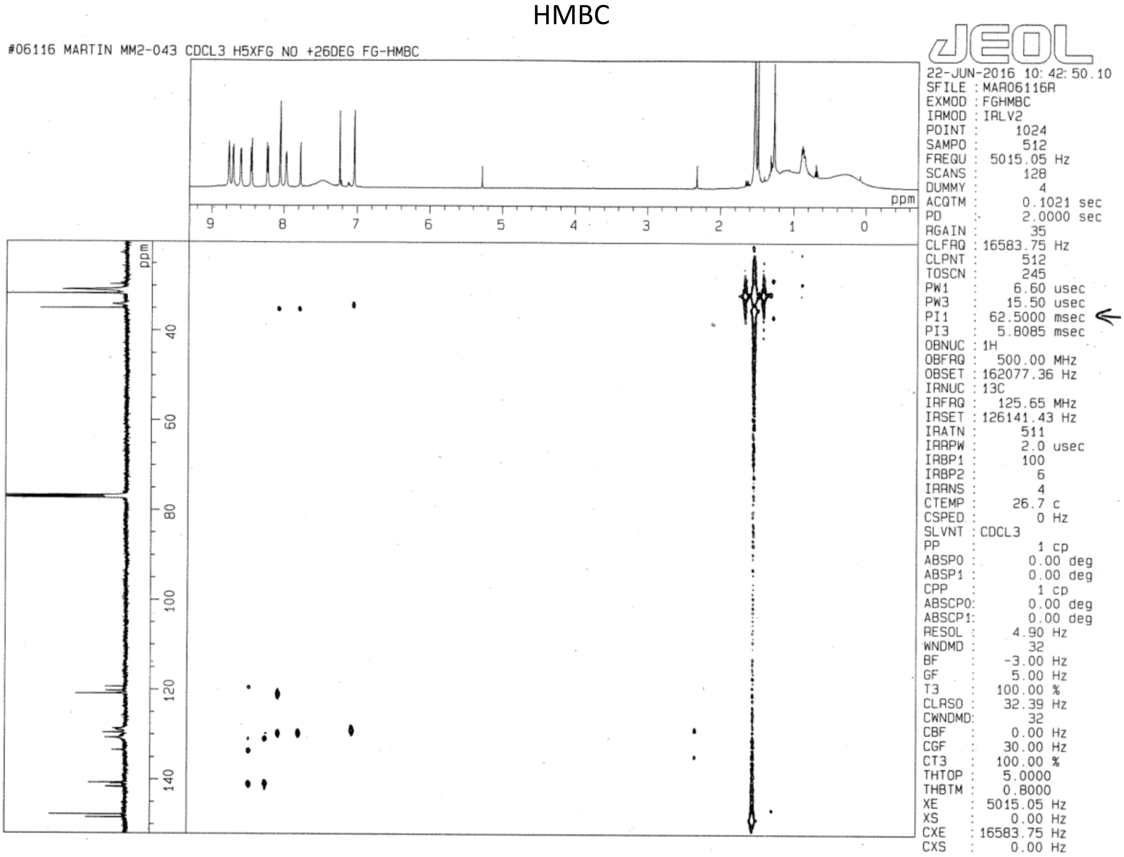

Figure S38. ^{1}H-^{13}C-HMBC of hexa-porphyrin-HPB **10**.

HRMS (ESI)

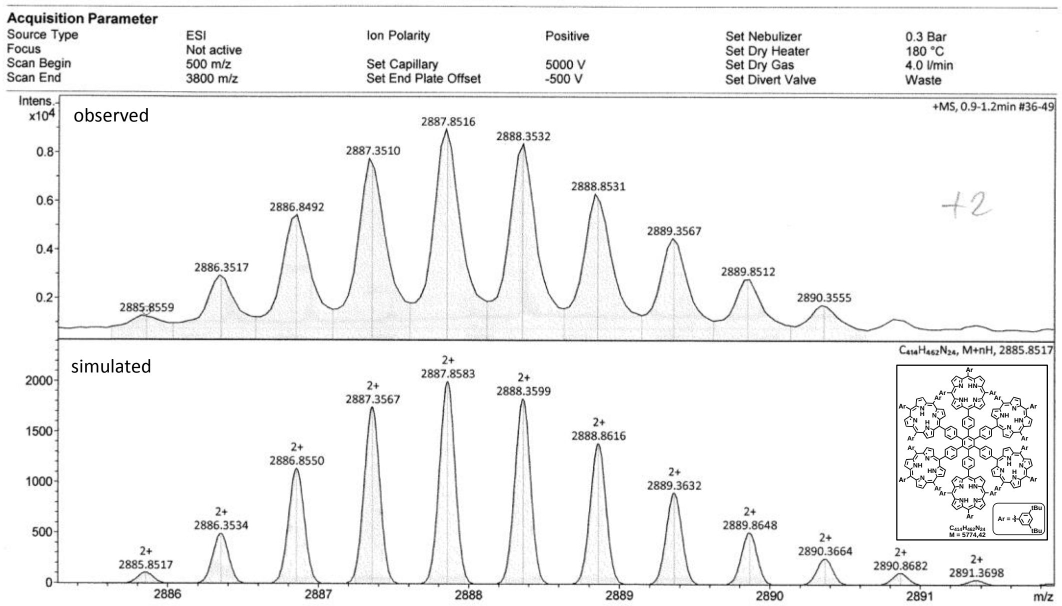

Meas. m/z	#	Ion Formula	m/z	err [ppm]	Mean err [ppm]	rdb	N-Rule	e⁻ Conf	mSigma	Std I	Std Mean m/z	Std I VarNorm	Std m/z Diff	Std Comb Dev
2885.855858	1	C414H464N24	2885.851747	-1.4	954.8	195.0	ok	even	222.0	166.6	n.a.	n.a.	n.a.	n.a.
	2	C415H466N23	2885.858035	0.8	957.1	194.5	ok	odd	222.9	167.1	n.a.	n.a.	n.a.	n.a.

Figure S39. HRMS (ESI) of hexa-porphyrin-HPB **10**.

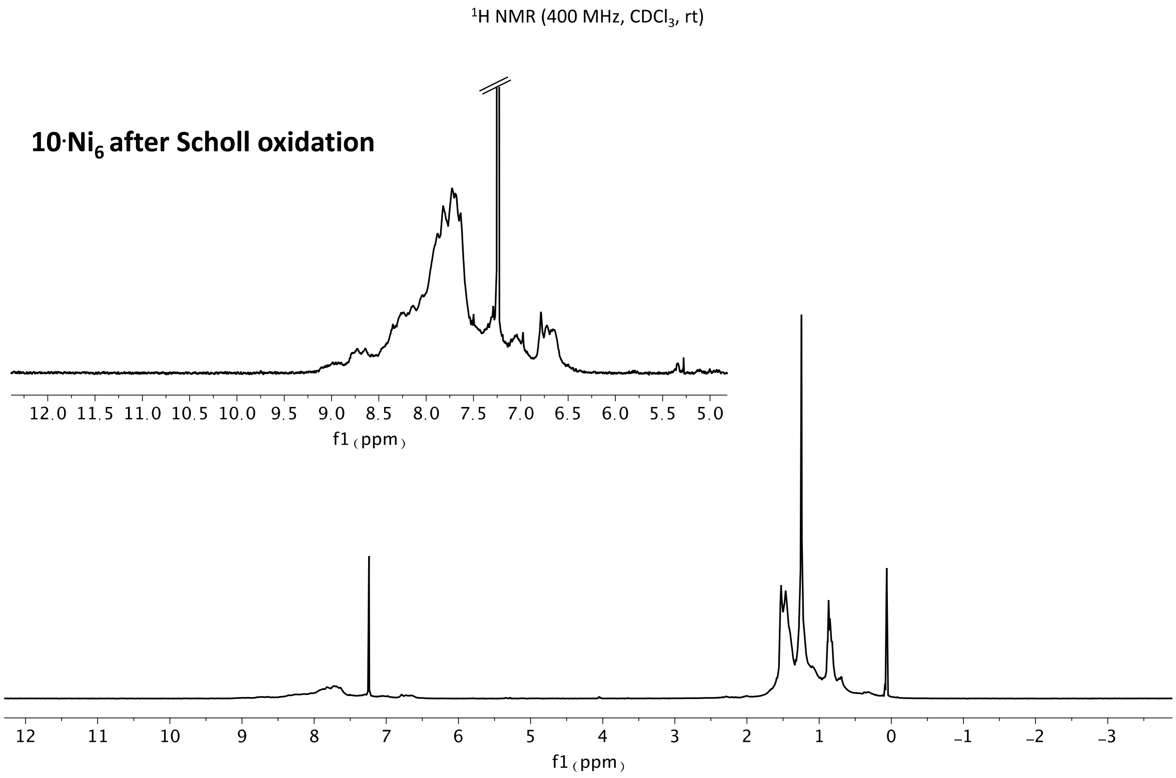

Figure S40. ¹H NMR spectrum of hexa-nickel-porphyrin HPB **10·Ni$_6$** after Scholl oxidation. Mixture of isomers.

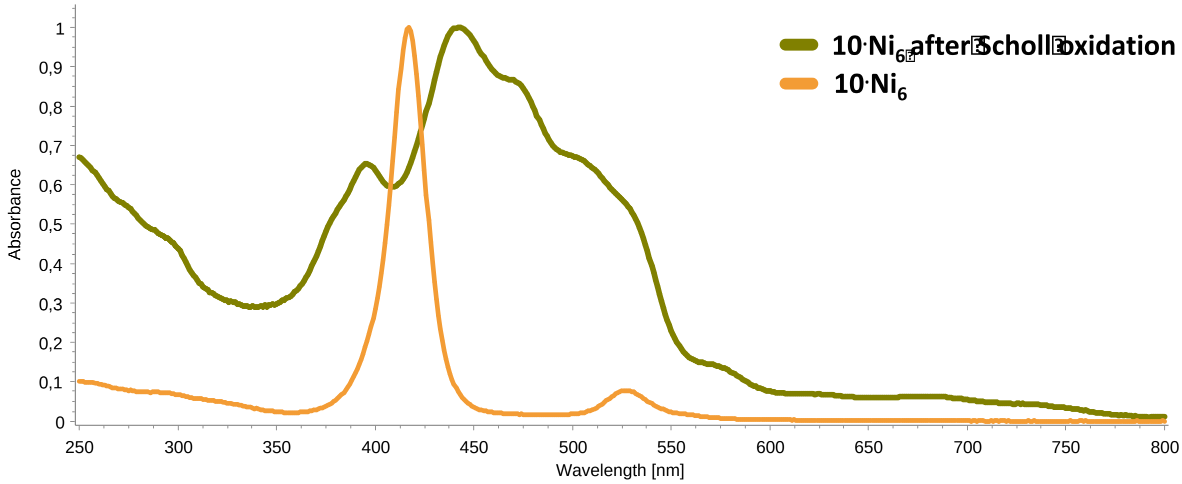

Figure S41. Normalized UV/Vis spectrum of hexa-nickel-porphyrin HPB **10·Ni$_6$** before (orange) and after (green) Scholl oxidation.

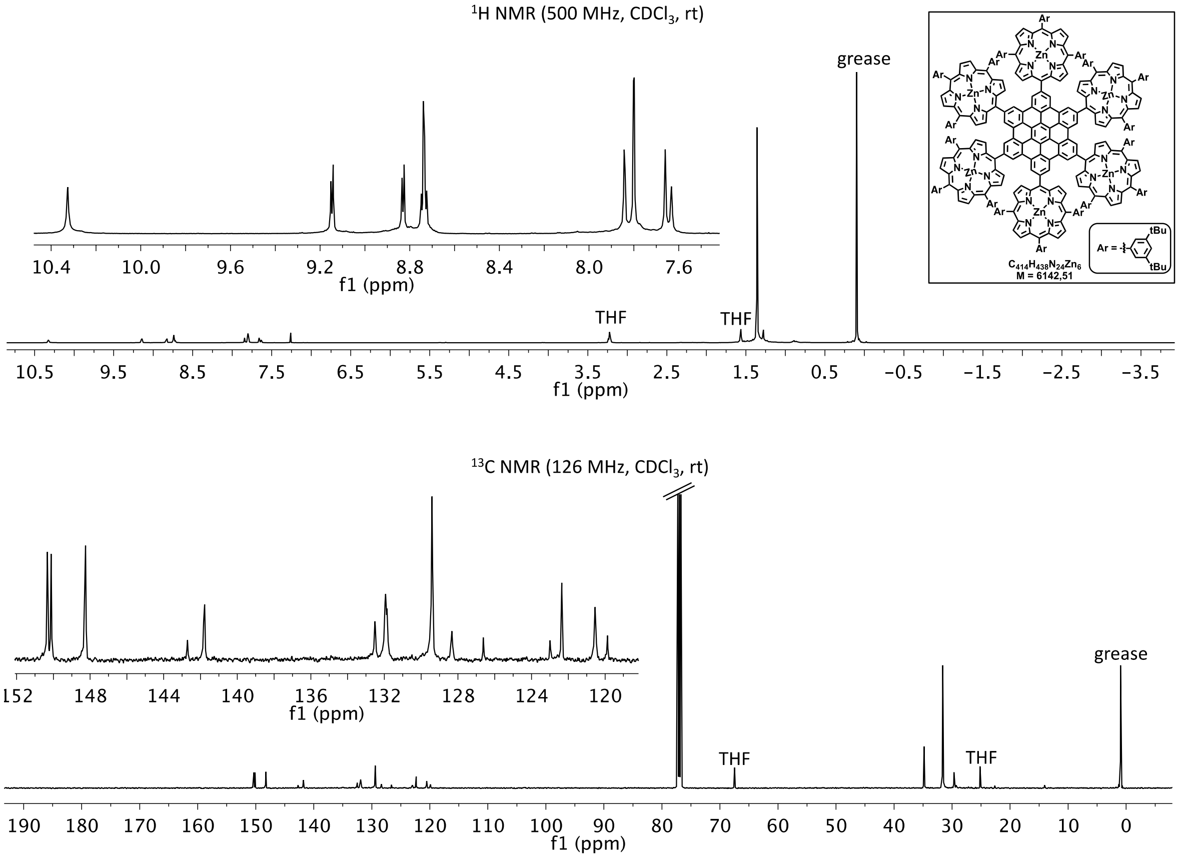

Figure S42. ^{1}H and ^{13}C NMR of hexa-zinc-porphyrin-HBC **11**$^{\cdot}$**Zn**$_6$.

COSY

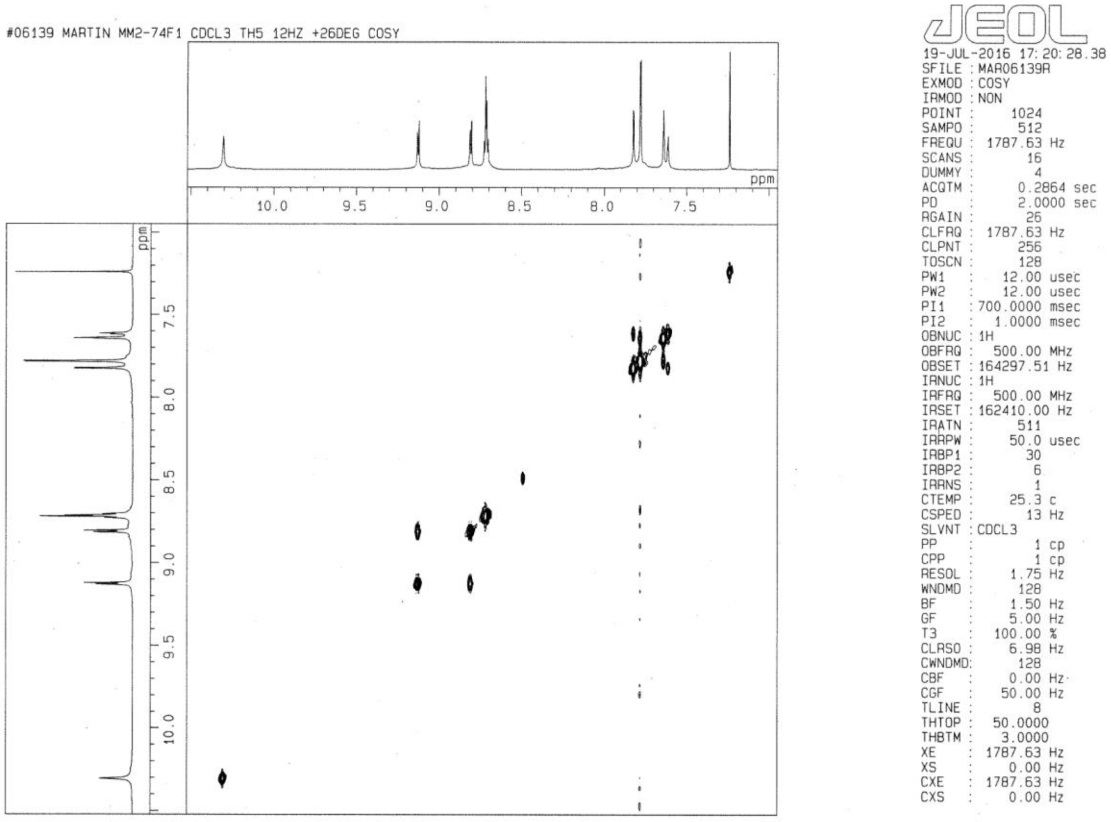

Figure S43. ^{1}H-^{1}H COSY of hexa-zinc-porphyrin-HBC **11**$^{\cdot}$**Zn**$_6$.

HSQC

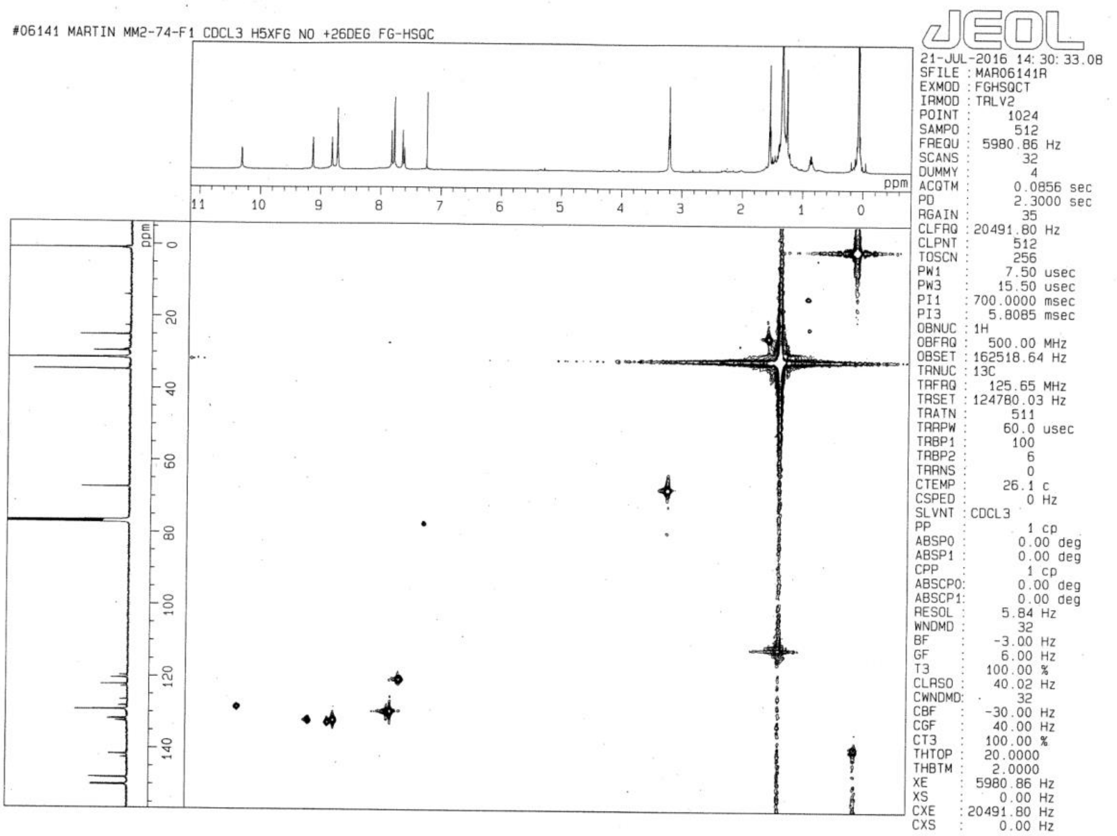

Figure S44. ^{1}H-^{13}C-HSQC of hexa-zinc-porphyrin-HBC **11$\cdot$Zn$_6$**.

HMBC

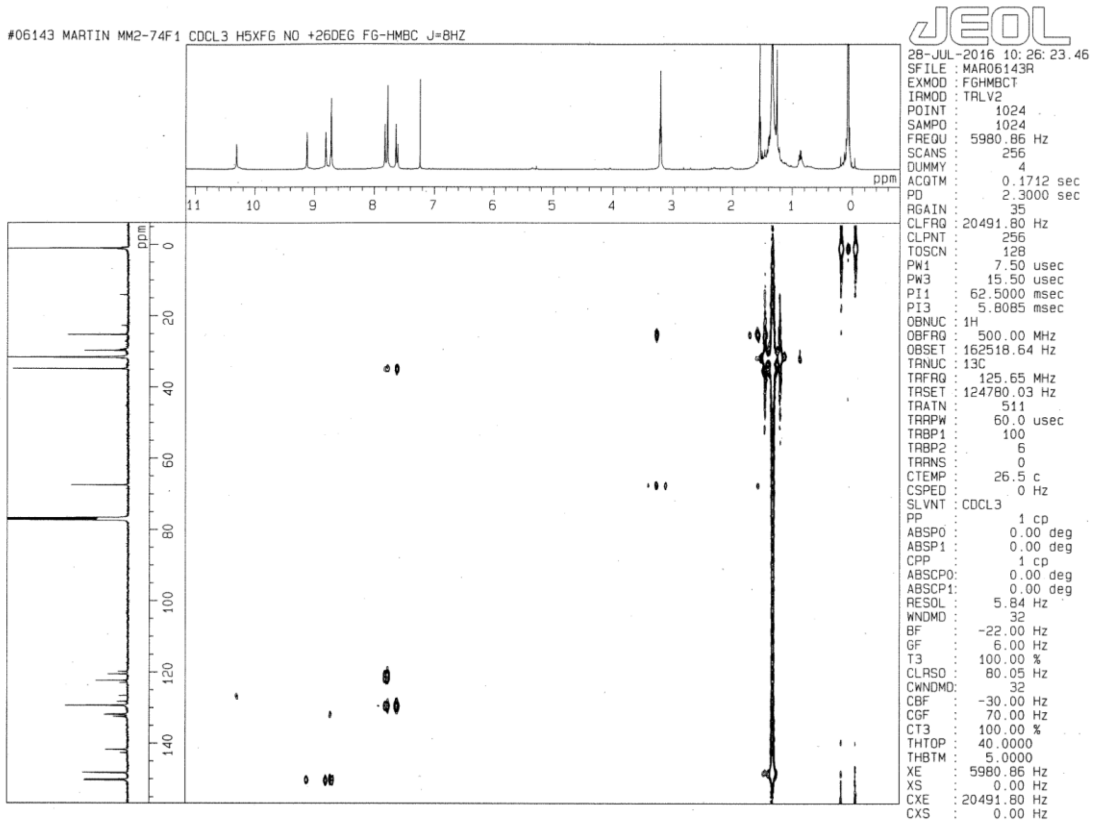

Figure S45. ^{1}H-^{13}C-HMBC of hexa-zinc-porphyrin-HBC **11$\cdot$Zn$_6$**.

HRMS (ESI)

Acquisition Parameter

Source Type	ESI	Ion Polarity	Positive	Set Nebulizer	0.3 Bar
Focus	Not active			Set Dry Heater	180 °C
Scan Begin	1000 m/z	Set Capillary	6000 V	Set Dry Gas	4.0 l/min
Scan End	3400 m/z	Set End Plate Offset	-500 V	Set Divert Valve	Waste

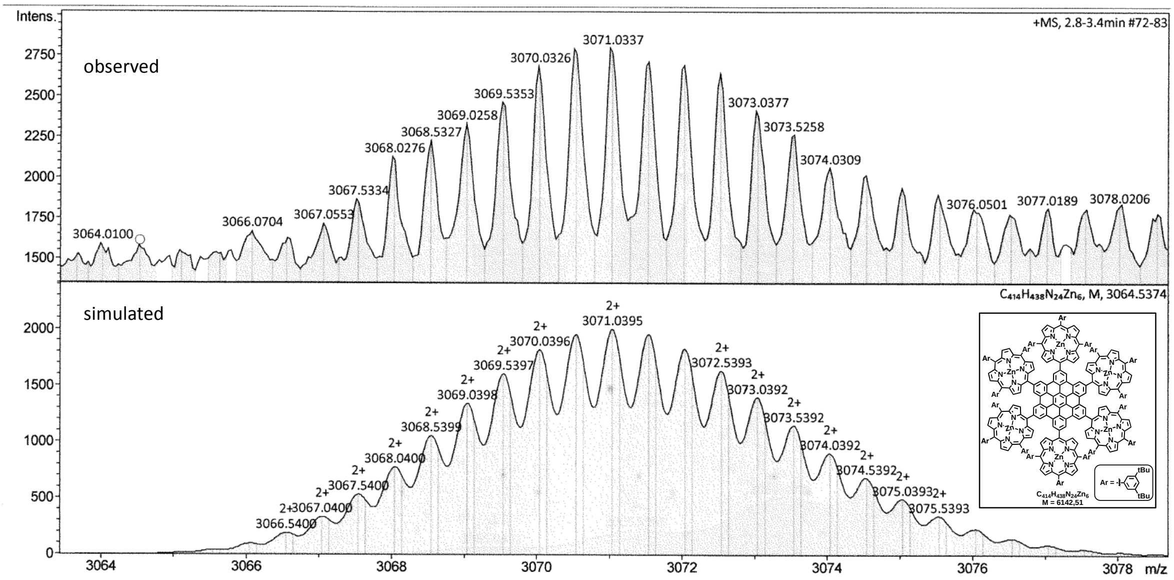

Meas. m/z	#	Ion Formula	m/z	err [ppm]	mSigma	# mSigma	Score	rdb	e⁻ Conf	N-Rule
3064.5344	1	C414H438N24Zn6	3064.5374	1.0	382.3	1	100.00	208.0	even	ok

Figure S46. HRMS (ESI) of hexa-zinc-porphyrin-HBC **11·Zn$_6$**.

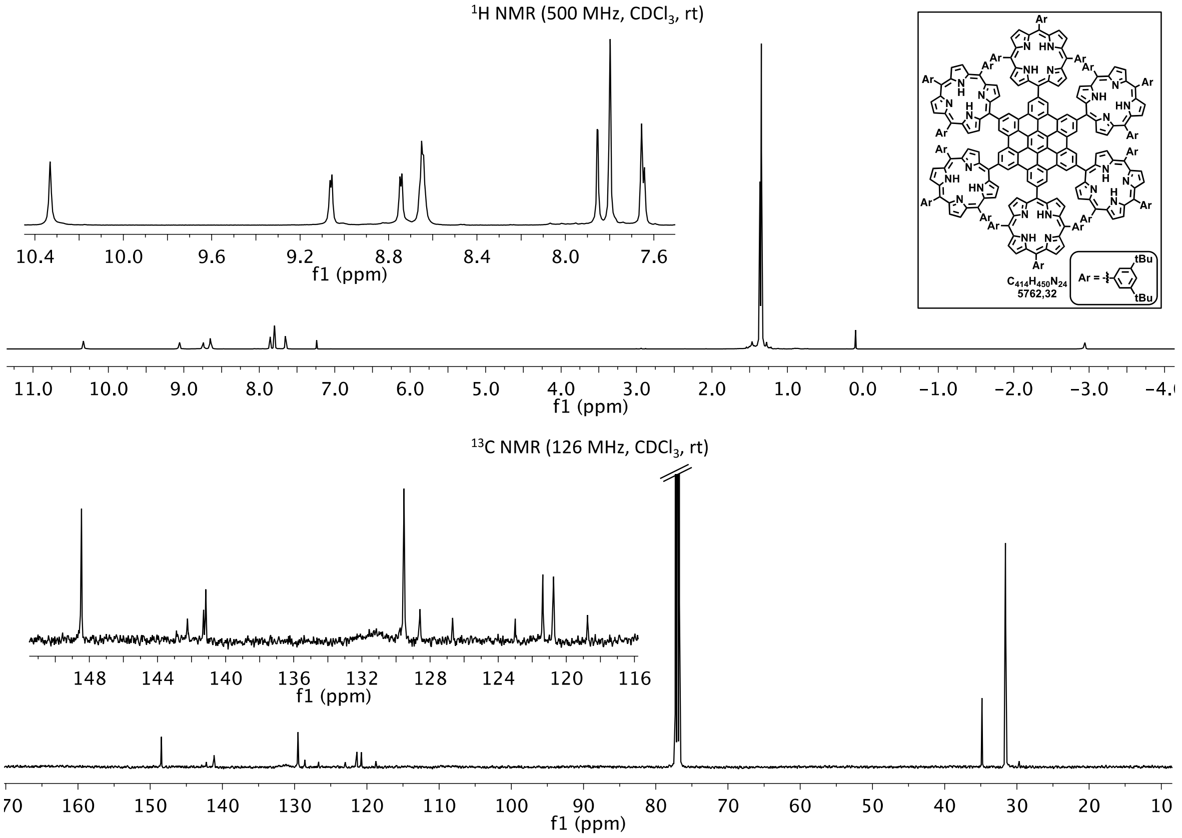

Figure S47. ^{1}H and ^{13}C NMR of hexa-free-base-porphyrin-HBC **11**.

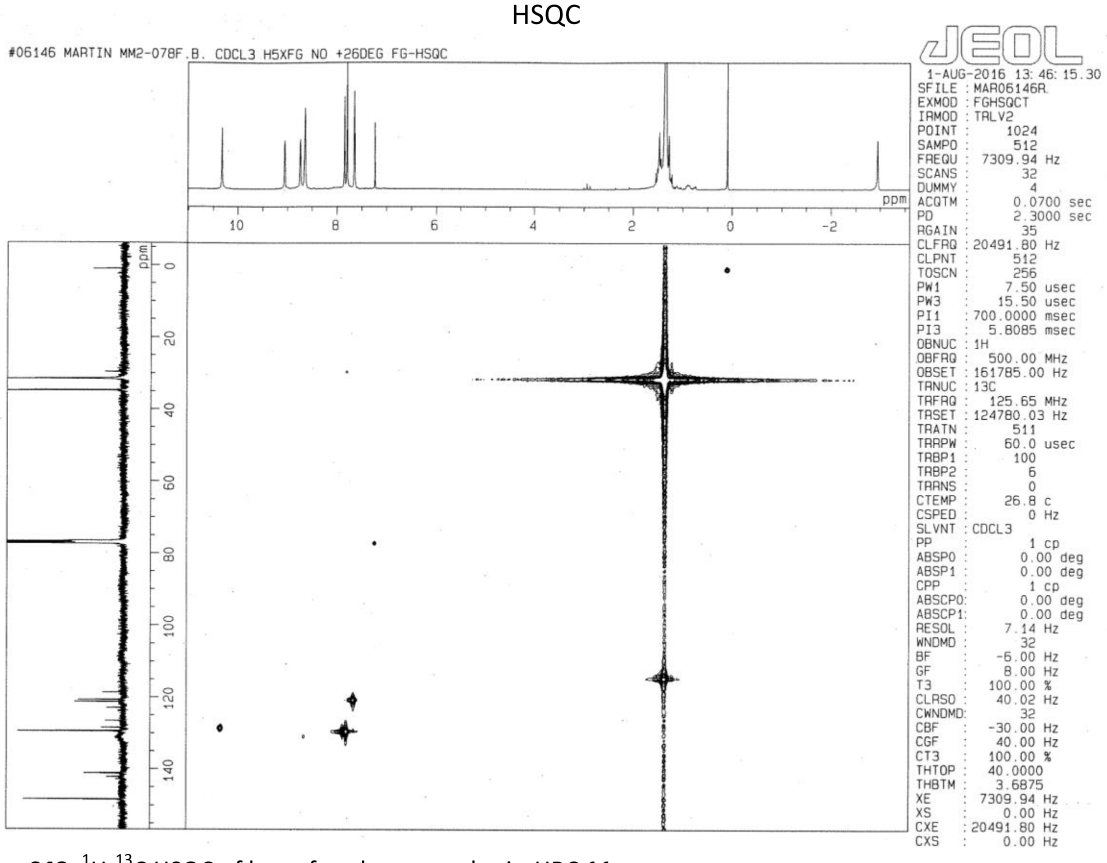

Figure S48. ^{1}H-^{13}C HSQC of hexa-free-base-porphyrin-HBC **11**.

HMBC

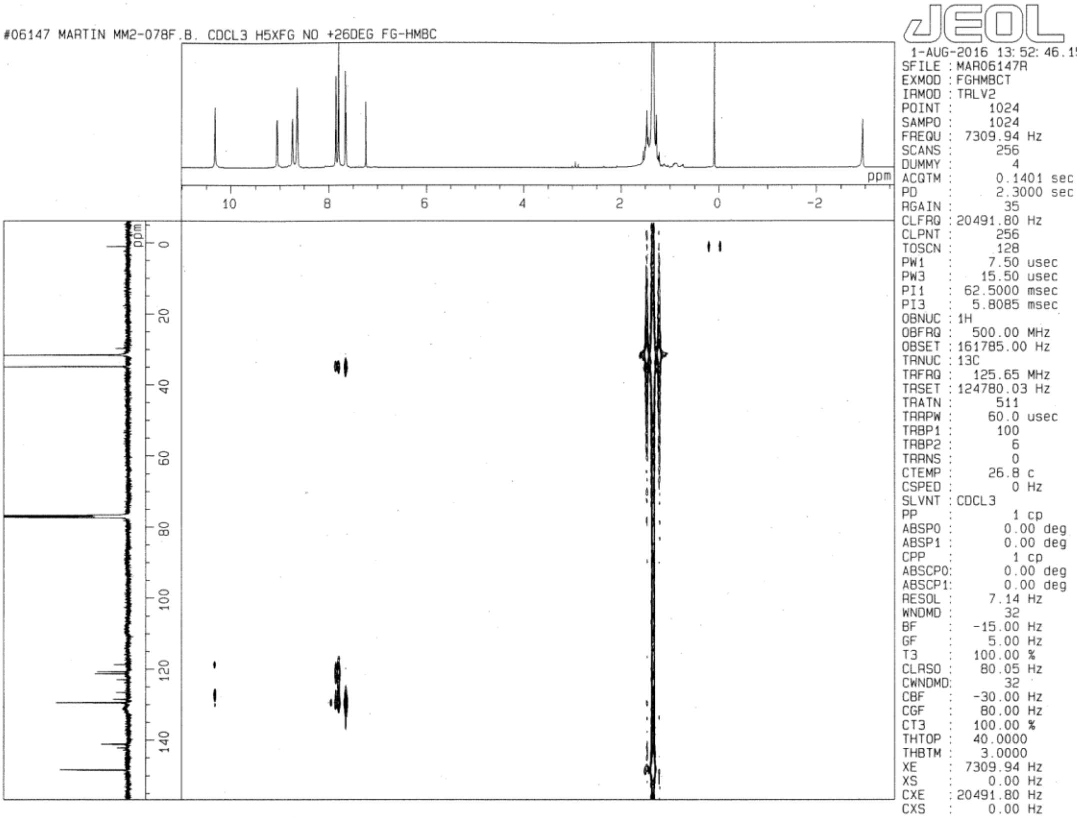

Figure S49. ^{1}H-^{13}C HMBC of hexa-free-base-porphyrin-HBC **11**.

HRMS (ESI)

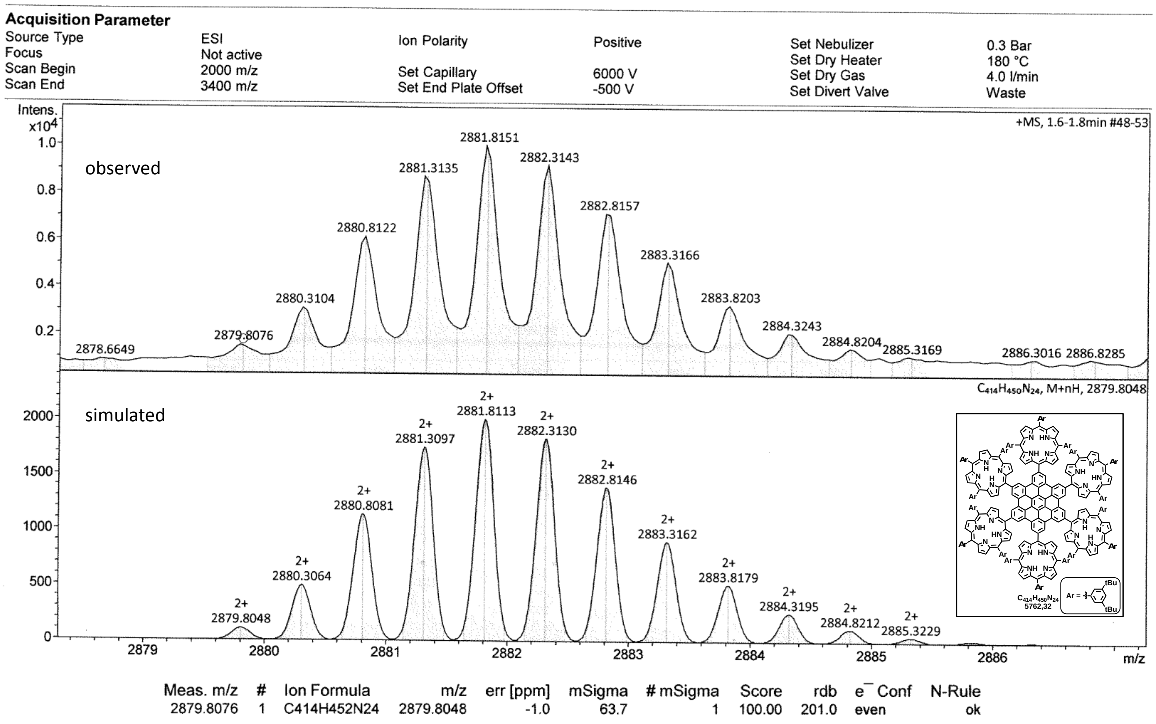

Figure S50. HRMS (ESI) of hexa-free-base-porphyrin-HBC **11**.

6 References

[1] M. M. Martin, M. Dill, J. Langer, N. Jux, *J. Org. Chem.* **2019**, *84*, 1489-1499.

[2] D. Reger, P. Haines, F. W. Heinemann, D. M. Guldi, N. Jux, *Angew. Chem. Int. Ed.* **2018**, *57*, 5938-5942.

[3] M. M. Martin, D. Lungerich, P. Haines, F. Hampel, N. Jux, *Angew. Chem. Int. Ed.* **2019**, *58*, 8932-8937.

[4] M. Balaz, H. A. Collins, E. Dahlstedt, H. L. Anderson, *Org. Biomol. Chem.* **2009**, *7*, 874-888.

[5] M. A. Fazio, A. Durandin, N. V. Tkachenko, M. Niemi, H. Lemmetyinen, D. I. Schuster, *Chem. Eur. J.* **2009**, *15*, 7698-7705.

[6] D. Lungerich, J. F. Hitzenberger, F. Hampel, T. Drewello, N. Jux, *Chem. Eur. J.* **2018**, *24*, 15818-15824.

[7] M. Takase, R. Ismael, R. Murakami, M. Ikeda, D. Kim, H. Shinmori, H. Furuta, A. Osuka, *Tetrahedron Lett.* **2002**, *43*, 5157-5159.

[8] Y. Terazono, G. Kodis, K. Bhushan, J. Zaks, C. Madden, A. L. Moore, T. A. Moore, G. R. Fleming, D. Gust, *J. Am. Chem. Soc.* **2011**, *133*, 2916-2922.

[9] D. J. Jones, B. Purushothaman, S. Ji, A. B. Holmes, W. W. H. Wong, *Chem. Commun.* **2012**, *48*, 8066-8068.

[10] K. Kobayashi, N. Kobayashi, M. Ikuta, B. Therrien, S. Sakamoto, K. Yamaguchi, *J. Org. Chem.* **2005**, *70*, 749-752.

[11] D. Mössinger, D. Chaudhuri, T. Kudernac, S. Lei, S. De Feyter, J. M. Lupton, S. Höger, *J. Am. Chem. Soc.* **2010**, *132*, 1410-1423.

[12] J. Wu, A. Fechtenkötter, J. Gauss, M. D. Watson, M. Kastler, C. Fechtenkötter, M. Wagner, K. Müllen, *J. Am. Chem. Soc.* **2004**, *126*, 11311-11321.

[13] O. V. Dolomanov, L. J. Bourhis, R. J. Gildea, J. A. K. Howard, H. Puschmann, *J. Appl. Crystallogr.* **2009**, *42*, 339-341.

[14] G. Sheldrick, *Acta Crystallographica Section A* **2015**, *71*, 3-8.

[15] G. Sheldrick, *Acta Crystallographica Section C* **2015**, *71*, 3-8.

[16] Rigaku Oxford Diffraction, 2015, CrysAlisPro Software system, version 1.171.38.46, Rigaku Corporation, Oxford, UK.

[17] A. Thorn, B. Dittrich, G. M. Sheldrick, *Acta Crystallographica Section A* **2012**, *68*, 448-451.

[18] P. Van Der Sluis, A. L. Spek, *Acta Crystallographica Section A* **1990**, *46*, 194-201.

[19] J.-S. Jiang, A. T. Brünger, *J. Mol. Biol.* **1994**, *243*, 100-115.

[20] D. Allan, H. Nowell, S. Barnett, M. Warren, A. Wilcox, J. Christensen, L. Saunders, A. Peach, M. Hooper, L. Zaja, S. Patel, L. Cahill, R. Marshall, S. Trimnell, A. Foster, T. Bates, S. Lay, M. Williams, P. Hathaway, G. Winter, M. Gerstel, R. Wooley, *Crystals* **2017**, *7*, 336.

[21] CrysalisPro, Agilent Technologies Yarton, Oxfordshire, UK, **2009-2016**.

[22] L. Farrugia, *J. Appl. Crystallogr.* **2012**, *45*, 849-854.

[23] Bruker-Nonius, *APEX, SAINT and XPREP*, Bruker AXS Inc., Madison, Wisconsin, USA, **2013**.

[24] G. Bricogne, E. Blanc, M. Brandle, C. Flensburg, P. Keller, W. Paciorek, P. Roversi, A. Sharff, O. S. Smart, C. Vonrhein, T. O. Womack, *BUSTER*, 2.11.2 ed., Global Phasing Ltd., Cambridge, United Kingdom, **2011**.

[25] O. S. Smart, T. O. Womack, *Grade Web Server*, Global Phasing Ltd. , **2014**.

[26] A. L. Spek, *PLATON: A Multipurpose Crystallographic Tool*, Utrecht University, Utrecht, The Netherlands, **2008**.
[27] I. Guzei, *J. Appl. Crystallogr.* **2014**, *47*, 806-809.
[28] Spartan'16 Parallel Suite (Version 2.0.7).
[29] F. Menges "Spectragryph - optical spectroscopy software", Version 1.0.3, **2017**, http://www.effemm2.de/spectragryph/.
